# Transition Metal‐Free Direct Electrochemical Carboxylation of Organic Halides Using a Sacrificial Magnesium Anode: Straightforward Synthesis of Carboxylic Acids

**DOI:** 10.1002/open.202400426

**Published:** 2025-01-28

**Authors:** Iryna Lesko, Stéphane Sengmany, Raphaël Beltran, Erwan Le Gall, Eric Léonel

**Affiliations:** ^1^ University Paris Est Creteil CNRS ICMPE, UMR 7182, 2 rue Henri Dunant, 94320 Thiais France; ^2^ Sanofi, 45 Chemin de Meteline 04200 Sisteron France

**Keywords:** Electrosynthesis, Sacrificial anode, Direct electrolysis, Organic bromides, Carboxylic acids

## Abstract

The direct electrochemical carboxylation of aryl, benzyl and alkyl halides by CO_2_ is described using a magnesium anode and a nickel foam cathode in an undivided cell. The process employs a sacrificial anode and does not require the additional use of a transition metal catalyst or demanding conditions, as the reactions are carried out under galvanostatic mode, at −10 °C and with commercial DMF. Under these operationally simple conditions, an important range of carboxylic acids are affordable. Mechanistic investigation account for the *in situ* generation of a carbanionic species that is not a simple organomagnesium halide.

## Graphical abstract

The direct electrochemical carboxylation of organic halides was carried out in an undivided cell fitted with a sacrificial magnesium anode and a nickel foam cathode. Mechanism investigations account for the in situ generation of an anionic species that is not a genuine Grignard reagent.

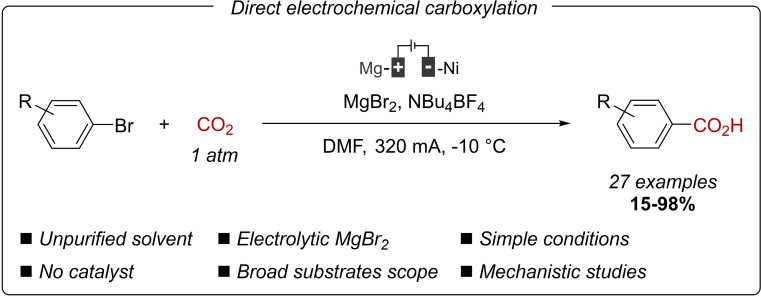

## Introduction

**Figure 1:**
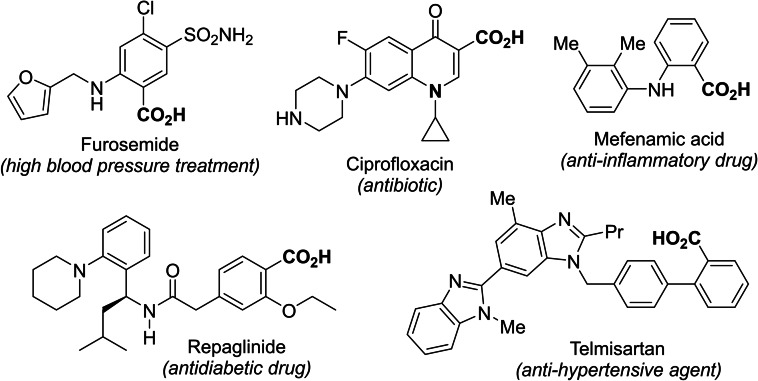
Some carboxylic acids displaying a biological activity.

Carboxylation is an extremely useful reaction in the pharmaceutical field because carboxylic acids represent important structural units that can be found in many natural and synthetic compounds displaying medicinal properties (Figure 1).^[1]^

Since the 1950s, carboxylic acids were frequently obtained on an industrial scale by the reaction between an organomagnesium reagent^2^, ^3^ and carbon dioxide. However, such processes are rather demanding, as they usually require anhydrous solvents, strict control of the temperature to obviate hazardous exothermic events, long reaction times and inert atmosphere.^[4]^ In addition, a magnesium pre‐activation step can be mandatory to remove the oxide layer formed on the metal surface^[5]^ and functional group tolerance is limited. These conditions are disadvantageous both in terms of reproducibility and from an environmental and economic point of view. In the mid‐1970s, Heck and coworkers developed a transition metal‐catalyzed carbonylation of aryl halides in the presence of carbon monoxide and various nucleophiles.^[6]^ This method, which has been widely exploited thereafter and especially in the 2000s,^[7]^ leads to carboxylic acids when the used nucleophile is water.^[8]^ However, this method also suffers some drawbacks, as carbon monoxide is a toxic, highly flammable, colorless and odorless gas, which can cause some severe hazard issues. Since the beginning of the 2010s, the direct use of carbon dioxide in replacement of carbon monoxide has attracted considerable attention. Indeed, carbon dioxide can be considered an ideal C1 synthon for its non‐toxicity, low cost and renewability. However, this unit is not easy to activate under mild conditions due to its thermodynamic stability and kinetic inertia.^[9]^ In this context, transition metal‐catalyzed methods for the direct carboxylation of aryl halides or pseudo‐halides are very attractive as they can overcome the poor reactivity of CO_2_.^10^, ^11^, ^12^, ^13^ However, they often require the additional presence over a stoichiometric amount of metallic reductants such as zinc or manganese to generate the active catalyst *in situ*,^[14]^ which represents a significant constraint for an industrial scale development.

**Scheme 1:**
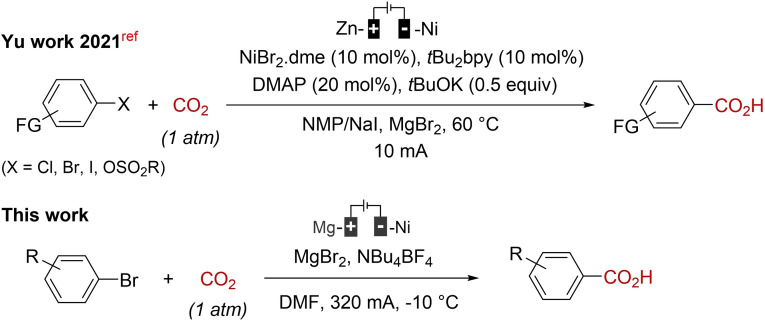
Electrochemical carboxylation reactions.

The past few years have witnessed a renewed and sustained interest in organic electrosynthesis as an alternative approach to more conventional chemical methods.^[15]^ Main advantages are selectivity and safety, especially on a large scale, since the reaction's rates can be mastered simply by acting on the current delivery. In this context, the electrochemical carboxylation of halides or pseudo‐halides is of current interest.^[16]^ However, some of these methods are difficult to implement, given the strict operating conditions (glovebox) and/or costly metal catalyst/ligand systems they feature. In the mid‐80s, Périchon et al. developed the electrochemical carboxylation of aryl halides by direct electrolysis using the consumable anode process.^[17]^ The method has the advantage of not using metal catalysts and is simple to carry out.^[18]^ However, yields of carboxylic acids were based on conversion ratios (determined by GC titration) of aryl halide substrates and the mechanism of the reaction was not discussed. Therefore, we decided to revisit the direct electrochemical carboxylation of organic halides using a sacrificial anode and give herein a deeper insight into the reaction mechanism (Scheme 1).

## Results and Discussion

**Scheme 2:**
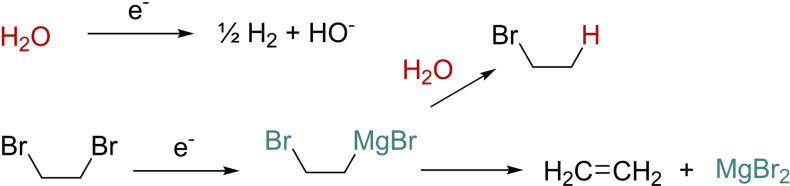
Principle of the pre‐electrolysis.

In order to determine the feasibility of this direct approach to carboxylic acids and especially the aromatic ones, a preliminary set of experiments was conducted on bromobenzene in DMF as the solvent (the initial choice of a bromide was motivated by the particularly good compromise between commercial availability, cost and electrochemical reactivity) using a magnesium anode and a nickel foam cathode as the electrode. These experiments revealed that NBu_4_BF_4_ was a suitable supporting electrolyte due to both important solubility and induced conductivity of the electrolysis medium. We could also observe that the presence of MgBr_2_ was mandatory to achieve satisfactory conversions. Due to reproducibility issues, we also evaluated the water amount in the reaction solvent using Karl Fischer experiments, which returned a 1.5 % water amount in freshly opened bottles of commercial DMF. For these reasons, we chose to systematically carry out a pre‐electrolysis of the reaction medium in the presence of 1,2‐dibromoethane before addition of the organic halide and CO_2_ bubbling. Such a preliminary step has multiple benefits as it allows both an initial drying of the medium as well as providing electrolytic MgBr_2_ for further chemical purposes (Scheme 2).

Indeed, depending on the reaction conditions and especially cathode material, water can be firstly reduced to dihydrogen and hydroxide at the electrode whereas 1,2‐dibromoethane is then reduced to an anionic species that associates to Mg^2+^ resulting from the anode oxidation to form an organomagnesium equivalent. The important basicity of this species allows trapping of the water residue of the solution. Both processes can take place concomitantly or in the reversed order. When water is no more present in the medium, the organomagnesium equivalent, which is continuously produced, decomposes to ethylene and magnesium bromide. It can be noted that depending on the water amount at the beginning, the production of MgBr_2_ may be variable.

With these preliminary results in hand, the reaction conditions were then optimized with 4‐bromoanisole, since this compound allows easier GC detection of the potential (by)products of the electrolysis (biaryl, anisole, acid etc…). Results are presented in Table 1.

**Table 1:** Optimization of the reaction conditions.^[a]^

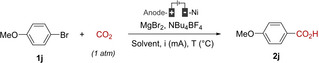							
Entry	T (°C)	I (mA)	Anode	Solvent	C (mol/L)	Time (h)	Yield (%)^[b]^
1	**20**	320	Mg	DMF	0.4	3.7	26
2	**0**	320	Mg	DMF	0.4	2.3	30
3	−10	320	Mg	DMF	0.4	3.3	46
4	−**20**	320	Mg	DMF	0.4	2.3	40
5	−**30**	320	Mg	DMF	0.4	3	31
6	−10	**640**	Mg	DMF	0.4	1.3	28
7	−10	**160**	Mg	DMF	0.4	6.5	27
8	−10	**50**	Mg	DMF	0.4	17	27
9	−10	**0**	Mg	DMF	0.4	24	n.r.
10	−10	320	**Al**	DMF	0.4	4.8	48
11	−10	320	**Zn**	DMF	0.4	4.3	n.r.
12	−10	320	Mg	**DMA**	0.4	2.7	11
13	−10	320	Mg	**NMP**	0.4	3	11
14	−10	320	Mg	**MeCN**	0.4	2.5	3
15	−10	320	Mg	DMF	**0.2**	3	50
16	−10	320	Mg	DMF	**0.6**	2.5	43

^[a]^ Reaction conditions: non‐compartmented cell fitted with a nickel foam cathode, using **1 j** (8 mmol) as the starting bromide, 20 mL solvent, and CO_2_ (g) at atmospheric pressure. Pre‐electrolysis: 30 min at 320 mA in the presence of 1,2‐dibromoethane (350 μL). ^[b]^ GC yield. In all cases, the hydro‐debromination product is the main side‐product observed.

The first point to note is that the reaction can be conducted at different temperatures (entries 1–4), although a temperature of −10 °C gave the best results (entry 3). The modification of the electric intensity applied under galvanostatic mode was increased (entry 6), then reduced progressively (entries 7 and 8) without improvement of the reaction yield. It can be noted that logically, the reaction does not occur in the absence of electric current (entry 9), thus indicating that we do not face an only chemical process. Metals other than magnesium were tested as the anode material (entries 10 and 11). In this case, whereas the use of a zinc rod anode did not furnish the desired product (entry 11), aluminum proved comparable to magnesium with a 48 % yield (entry 10). However, it can be noted that the faradic yield is decreased by ~50 %, thus indicating that the process is more energy‐consuming hence globally less efficient. Other solvents than DMF were then assessed in the electrochemical coupling (entries 12–14). However, none of these attempts were found sufficiently efficient to produce the corresponding carboxylic acid in descent yield. Finally, we explored the effect of the modification of the substrate concentration (entries 15 and 16). In this case, whereas an increase of the concentration by using 12 mmol of the starting substrate led to a slightly reduced yield (entry 16), we could observe that working at lower concentration can furnish increased amounts of the carboxylic acid (entry 15). However, in this case, the faradic yield dropped by half, thus corresponding to a globally less efficient chemical transformation.

**Figure 2:**
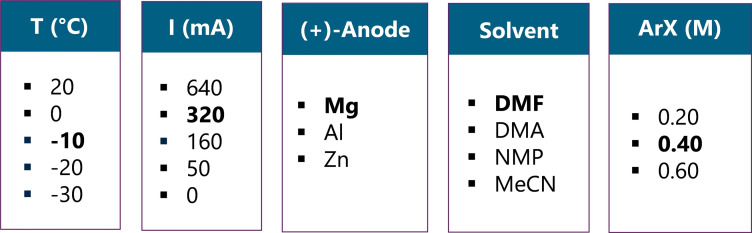
Summary of the optimized parameters.

Finally, the following parameters were kept for the rest of the study (Figure 2).

The scope of the reaction was then evaluated under these optimized conditions (Table 2).

**Table 2:**
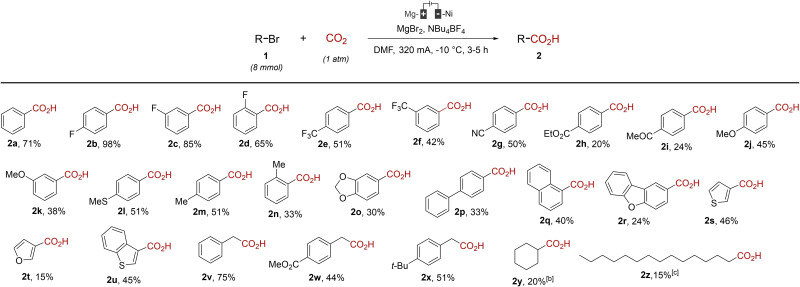
Scope of the reaction.^[a]^

^[a]^ Reaction conditions: Mg‐anode, Ni‐cathode, DMF (20 mL), NBu_4_BF_4_ (1 g), RBr (8 mmol, 0.4 M), CO_2_ (1 atm), 320 mA (constant current intensity), −10 °C. Pre‐electrolysis: 30 min at 320 mA in the presence of 1,2‐dibromoethane (350 μL). ^[b]^ Reaction carried out at 80 mA. ^[c]^ Reaction carried out at rt.

All the substrates gave the corresponding carboxylic acids although in highly variable yields (15–98 %). Bromobenzene **1 a** gave the coupling product **2 b** with good yield (70 %) whereas its *para*‐ and *meta*‐fluorinated counterparts **1 b** and **1 c** led to the final product with good and excellent yields, respectively. It can be noted that the *ortho*‐fluorinated bromobenzene **1 d** proved less efficient in the coupling (65 %), probably due to steric effects. When substituted with a trifluoromethyl group, bromobenzenes gave satisfactory yields (42 and 51 %) of the coupling products **2 e** and **2 f**. However, whereas the *para*‐cyanated bromobenzene **1 g** was able to undergo the reaction with a satisfactory result (yield = 50 %), the carbonylated bromides **1 h** and **1 i** gave the corresponding carboxylic acids in only modest yields (20 and 24 %). Bromobenzenes substituted by electrodonating groups **1 j**–**1 o** gave satisfactory yields of the coupling products, although the same steric effect than this observed with the fluorine substituent led to a decreased yield when a methyl substituent was at the *ortho* position (product **2 n**, 33 %). The corresponding coupling products were also obtained with 4‐bromobiphenyl (**1 p**), 1‐bromonaphthalene (**1 q**) and 2‐bromodibenzofuran **1 r** in low to modest yields. Some heterocyclic bromides **1 s‐**‐**1 u** also furnished the coupling products **2 s‐**‐**2 u** but in variable yields. It can be noted that bromothiophene derivatives **1 s** and **1 u** provided satisfactory yields of acids **2 s** (46 %) and **2 u** (45 %) whereas the reaction of 3‐bromofuran (**1 t**) was inefficient with an only low yield (15 %) being obtained. Benzylic bromides **1 v**–**1 x** were also assessed in the reaction and furnished the corresponding carboxylic acids in satisfactory to good yields. Aliphatic bromides **1 y** and **1 z** also underwent the reaction but in very limited yields.

**Figure 3:**
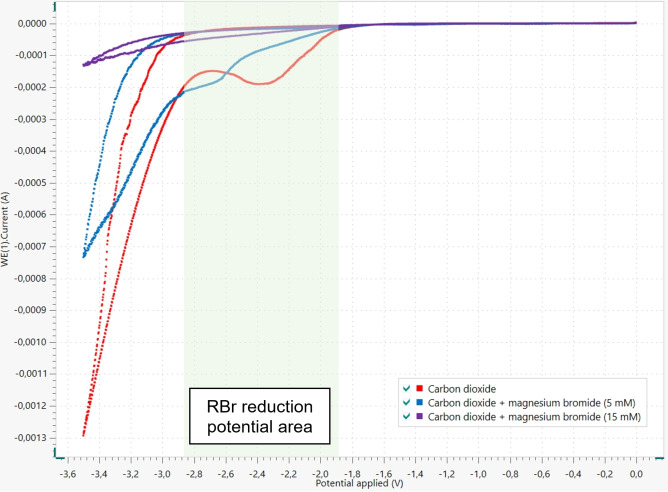
Cyclic voltamperograms of the reaction medium with increasing amounts of MgBr_2_.^[a]^ [a] Experimental conditions: Au‐working electrode, Au‐counter electrode, AgCl/Ag‐reference electrode, commercial MgBr_2_, NBu_4_BF_4_ (0.3 M), scan rate=100 mV/s.

At this stage, due to the above‐mentioned improved results observed in the presence of MgBr_2_, we tried to rationalize the role played by this salt in the process. Commonly, multiple roles can be attributed to MgBr_2_. When exposed to both nucleophilic and electrophilic species, it may not solely act as a Lewis acid, but should also promote the formation of ate complexes.^[19]^ In addition, under such electrochemical conditions, one can expect the increasing of the medium conductivity. To gather some additional information, we explored the electrochemical behavior of carbon dioxide in the presence of variable amounts of this salt. Therefore, cyclic voltamperograms of the reaction medium were recorded in the absence of MgBr_2_ than in the presence of MgBr_2_ 5 mM then 15 mM (Figure 3).

These experiments revealed that in the absence of MgBr_2_, the reduction wave of CO_2_ is observed near −2.4 V vs AgCl/Ag. However, in the presence of increasing amounts of MgBr_2_, a notable cathodic shift of the reduction potential occurs. Consequently, it can be postulated that in the presence of MgBr_2_, carbon dioxide is more difficult to reduce. As most of the bromides used in this study are reducible between −1.9 V and −2.9 V vs AgCl/Ag (see SI for details), one can likely imagine that CO_2_ reduction could interfere with RBr reduction in the absence of MgBr_2_ whereas RBr is reduced first when MgBr_2_ is present.

A potential central role of MgBr_2_ was thus considered for the notable improvement of the reaction condition. Indeed, given the significant importance of this salt, we speculated that possible anion exchanges would be deleterious for the electrochemical coupling. Therefore, we considered that the supporting electrolyte that was used before, NBu_4_BF_4_, should be advantageously replaced with NBu_4_Br, which may preserve the integrity of MgBr_2_ during the course of the reaction. Therefore, a set of electrochemical couplings were reproduced in the presence of NBu_4_Br, instead of NBu_4_BF_4_ (Table 3).

**Table 3:**
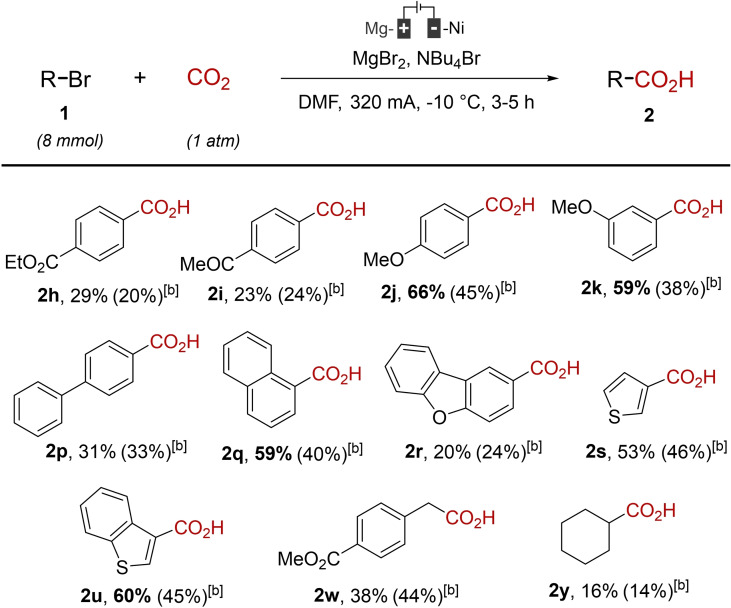
Additional assays with NBu_4_Br as the supporting electrolyte.^[a]^

^[a]^ Reaction conditions: Mg‐anode, Ni‐cathode, DMF (20 mL), NBu_4_Br (1 g), RBr (8 mmol, 0.4 M), CO_2_ (1 atm), 320 mA (constant current intensity), −10 °C. Pre‐electrolysis: 30 min at 320 mA in the presence of 1,2‐dibromoethane (350 μL). ^[b]^ Yield obtained using NBu_4_BF_4_ (reminder).

These additional experiments gave very interesting results. Indeed, it was found that the simple exchange between NBu_4_BF_4_ and NBu_4_Br was able to provide some significant improvements of the electrochemical coupling. It was notably the case for the formation of carboxylic acids **2 j**, **2 k**, **2 q** and **2 u**, for which an increasing of the reaction yield by c.a. 20 % is observed. However, surprisingly, the other models were less or not impacted at all by the salt exchange.

### Elements of Reaction Mechanism

**Scheme 3:**
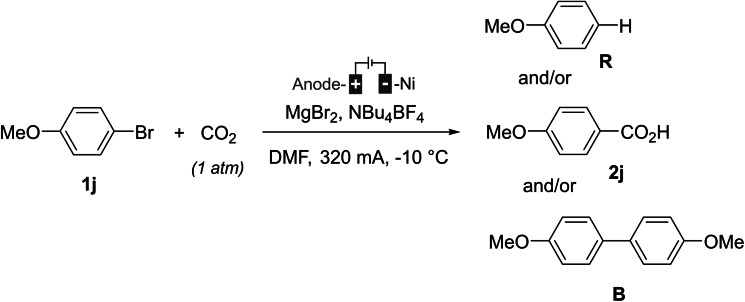
The model reaction and possible products.

Given the lack of insights into the mechanism of this direct (non‐catalyzed) carboxylation reaction, a mechanistic study was conducted on bromoanisole **1 j**. Plausible reaction products, the hydro‐dehalogenation product **R**, the carboxylic acid **2 j**, and the biaryl **B** are depicted in Scheme 3.

**Figure 4:**
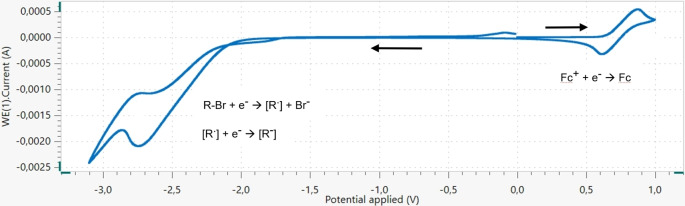
Cyclic voltamperogram of the reaction medium in the presence of 4‐bromoanisole and a stoichiometric amount of ferrocene.^[a]^ [a] Scan conditions: DMF (20 mL), vitreous carbon‐working cathode, Au‐counter electrode, AgCl/Ag‐reference electrode, 4‐bromoanisole (0.05 M), ferrocene 0.05 M), NBu_4_BF_4_ (0.3 M), scan rate =50 mV/s.

We first tried to determine whether the electrochemical reduction proceeds through a rapid two‐electron transfer to furnish an anionic species or not. In this purpose, cyclic voltamperograms of the typical electrolysis solution containing 4‐bromoanisole (**1 j**) were recorded in the presence of a stoichiometric amount of ferrocene (Figure 4).

The cyclic voltamperogram profile allowed us to determine that a two‐electron transfer rapidly occurs, as a single wave reduction of apparent intensity double that of the reduction of ferricinium (i1) is observed at 50 mV/s scan rate. It can be noted that the beginning of the scan is dedicated to the *in situ* generation of ferricenium from ferrocene by one‐electron oxidation. Then, at 1 V vs AgCl/Ag, the scan direction is reversed hence the cathodic scan begins by the one‐electron reduction of ferricenium, as this (stable) species is still present at the electrode. Therefore, one can imagine that a first electron is transferred to 4‐bromoanisole giving rise to a radical intermediate [R ⋅] that quickly undergoes a second electron transfer to form an anionic species [R−].

**Figure 5:**
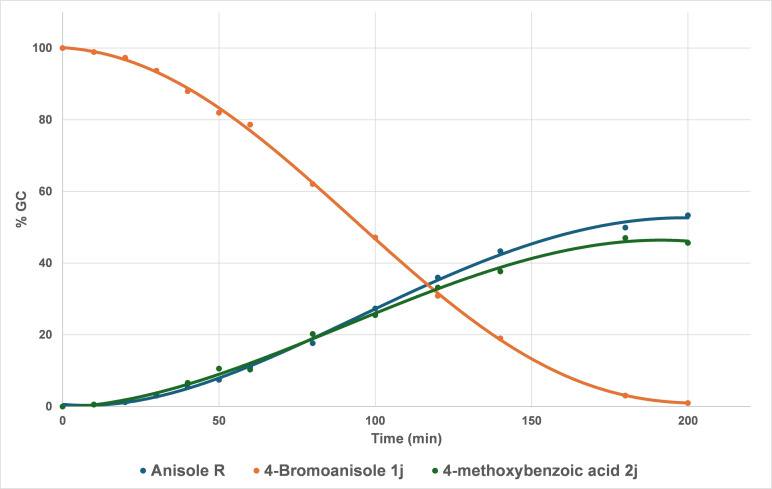
GC profile of the model reaction.

We next evaluated the reaction profile for the electrochemical carboxylation of anisole by conducting regular GC controls of aliquots during the course of the reaction (Figure 5).

The first point to note is that the conversion of the starting substrate is total after 200 min (~40 % faradic yield^[20]^). No important latency time is observable, as the consumption of bromoanisole **1 j** starts quickly after the beginning of the electrolysis. The profiles for the formation of the desired product, carboxylic acid **2 j**, and the hydro‐dehalogenation byproduct **R** are very similar. However, at this stage, it cannot be determined with accuracy whether the formation of **R** occurs *in situ* or during aliquot hydrolysis, even if the profile of the carboxylic acid formation rather accounts for negligeable production of this compound at the end of the electrolysis (no more anionic species available at the end of the electrolysis). It can also be noted that no biaryl **B** formation is observed thus accounting for an ionic mechanism rather than a radical one. More insights into the reaction mechanism were obtained in the following part of this work.

**Scheme 4:**
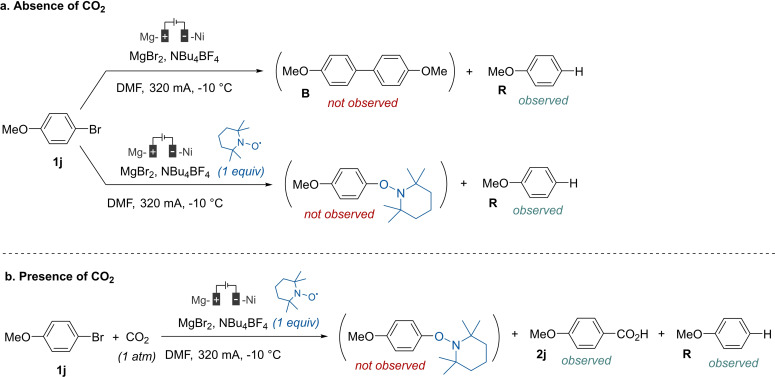
Verification of the anionic nature of the reaction.

We first tried to determine the nature of the reactive species (Scheme 4). Therefore, preparative electrolyses were conducted in the absence (Scheme 4a) or in the presence of carbon dioxide (Scheme 4b). Tempo was also added to evaluate its influence in the electrochemical coupling.

The first electrolysis was carried out without CO_2_ (Scheme 4a). In this case, it was assumed that if an aryl radical was formed, some traces of the corresponding biaryl would have been detectable by GC. However, only the hydro‐dehalogenation product **R** was observed. In the presence of the radical scavenger 2,2,6,6‐tetramethylpiperidin‐1‐yl)oxy (Tempo), no radical coupling adduct was observed. Product **R** was again the only observable compound by GC analysis. In the presence of CO_2_ (Scheme 4b), the fate of the reaction remained unchanged, the carboxylic acid **2 j** and the hydro‐dehalogenation **R** product being the sole species observed by GC. These results confirmed the rather anionic nature of the reactive species, hence electrogenerated from **1 j** by two‐electron transfer.

**Scheme 5:**
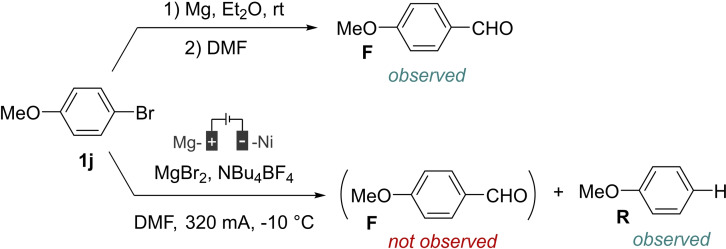
Reactivity of an organomagnesium and the electrogenerated anion with DMF.

We then tried to determine if the electrogenerated anionic species was an organomagnesium reagent or another anionic intermediate (Scheme 5).

**Scheme 6:**
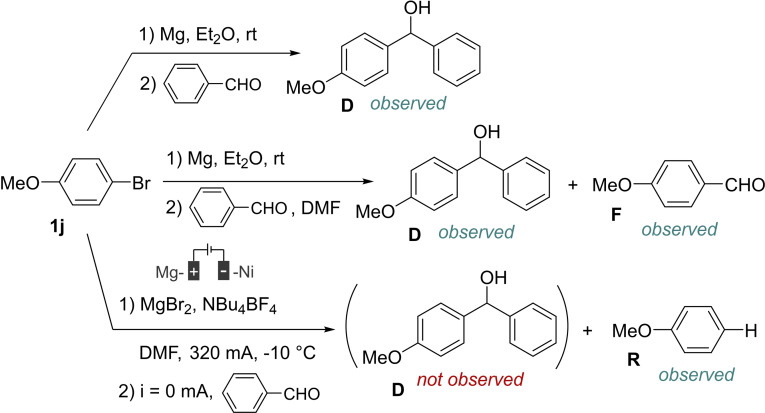
Reactivity of an organomagnesium and the electrogenerated anion with benzaldehyde.

Given the important nucleophilic character of an organomagnesium species, a formylation reaction would be expected with DMF. Therefore, we prepared by a chemical approach an organomagnesium reagent from **1 j** in diethyl ether prior to then addition of DMF. In this case, the formylation product **F** is mainly observed. However, no formylation product is observed from an electrochemical reduction in DMF, in the absence of CO_2_. The results account for the electrogeneration of an anionic species that is not an organomagnesium reagent. However, the conditions employed in both experiments are rather different since it would not be possible to electrogenerate the anionic species in diethyl ether then add DMF. Therefore, we confirmed the difference of reactivity between an organomagnesium reagent and the electrogenerated anionic species by additional experiments involving sequential formation of the nucleophilic species then addition of an electrophile, benzaldehyde (Scheme 6).

In a first experiment, the organomagnesium species arising from **1 j** was allowed to react with benzaldehyde, furnishing the expected diarylmethanol **D** as the major product. When an equimolar mixture of benzaldehyde and DMF was added to the organomagnesium reagent, we could observe the formation of a mixture of the products **D** and **F** resulting of the nucleophilic attack on the carbonyl of benzaldehyde, hence indicating that nucleophilic addition on both species can take place concomitantly. However, when the hypothetical nucleophilic species resulting from the electrolysis of **1 j**, and theoretically accumulated in the reaction medium at the end of the electrolysis, was allowed to react with benzaldehyde, no coupling product was observed, and the major product of the reaction was still the hydro‐dehalogenation product **R**, thus indicating that the storage of the anionic species is not possible in our standard experimental conditions. Unfortunately, given the reduction potentials involved, it was not possible to assess the electroreduction of **1 j** in the presence of benzaldehyde. However, these experiments gave interesting hints. As DMF and benzaldehyde react with an organomagnesium reagent at comparable reaction speeds (the formylation may be a sufficiently rapid process), it is probable that the electrogenerated anionic species is not an organomagnesium reagent. Indeed, in this case, considering that the reaction rate with carbon dioxide and DMF are also comparable, the formylation product would probably be observed in the reaction medium beside the carboxylic acid. We also found that it is not possible to store the anionic species during the course of the electrolysis. This account for the probable *in situ* protolysis of the anionic species or the formation of an anionic species that is inert toward benzaldehyde. In this latter case, the anionic species would present the selective ability to react with CO_2_ but not with DMF or benzaldehyde.

**Scheme 7:**
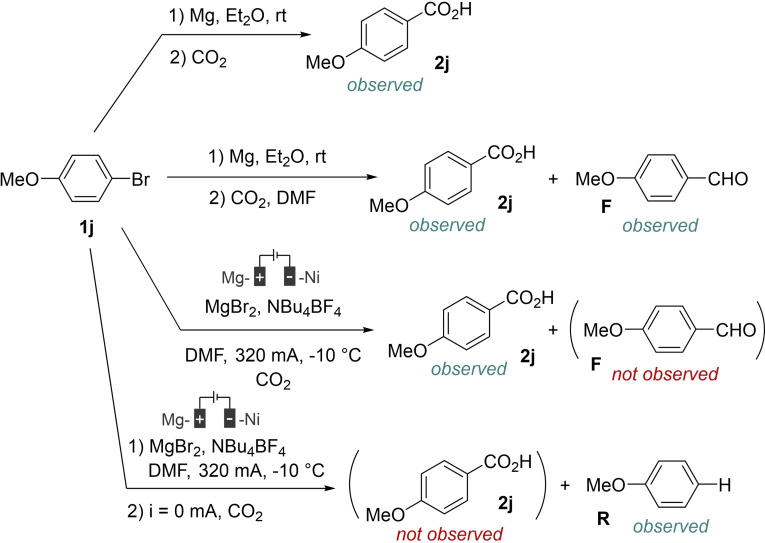
Reactivity of an organomagnesium and the electrogenerated anion with carbon dioxide.

The behavior of both nucleophilic species with carbon dioxide was next examined (Scheme 7).

We first verified that an organomagnesium reagent reacts with carbon dioxide to produce the corresponding acid. Then we again compared the relative rates of reaction of an organomagnesium reagent with carbon dioxide and DMF. This experiment revealed that reaction rates are again comparable, a mixture of the carboxylic acid and the aldehyde being detected in the reaction medium. We then tried to verify whether the electrogenerated anion can react stepwise with CO_2_. Unfortunately, contrary to what is observed in standard conditions (CO_2_ present in the reaction medium at the beginning of the electrolysis), no carboxylic acid is detected in the reaction medium and as with benzaldehyde as the electrophilic species, only the hydro‐dehalogenation product is observed. These observations account for the probable necessity to continuously trap the electrogenerated anionic species during the electrolysis. Indeed, all attempts to generate this species prior to the addition of an electrophile resulted to failures. Again, it can be postulated that the anionic species exhibits selective reactivity, as the trapping of CO_2_ appears possible even in the presence of important amounts of DMF (solvent). At this stage, considering the importance of MgBr_2_ in the electrochemical process and the relative inertness of the anionic species, we envisage that a stabilized organometallic species close to an organomagnesium is generated *in situ*. This species would be possibly an organomagnesiate equivalent RMgBr.MgBr_2_.^[21]^

**Scheme 8:**
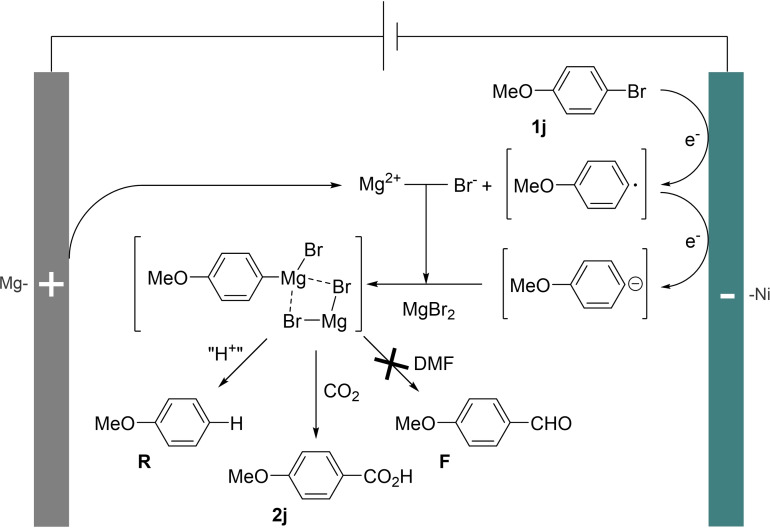
Possible reaction mechanism.

Taken together, these data allowed us to propose a potential reaction mechanism that is depicted in Scheme 8.

The organic bromide **1 j** would be reduced to an anionic species following a two‐electron transfer. This species would be sufficiently stabilized, maybe under the form of an organomagnesiate, to obviate nucleophilic addition to DMF and to exhibit a selective reactivity towards carbon dioxide, hence furnishing the corresponding carboxylic acid **2 j**. Concomitantly, due to the important basicity of the anionic species and the potential presence of proton sources in the reaction medium, the hydro‐dehalogenation product **R** would be formed by protolysis.

### Synthetic Application

**Scheme 9:**
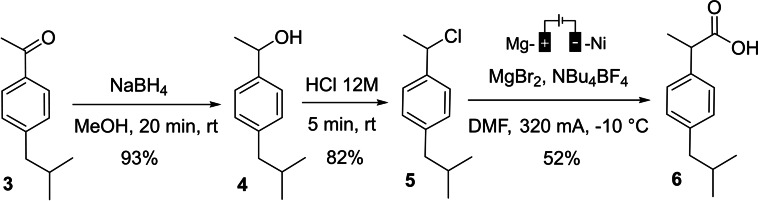
Application of the electrochemical reduction to the synthesis of ibuprofen.

The synthesis of the well‐known anti‐inflammatory drug ibuprofen employing electroreduction as the key step was carried out from commercial 4’‐isobutylacetophenone **3** (Scheme 9).^[22]^

Therefore, starting from ketone **3**, the corresponding alcohol **4** was obtained in 93 % yield by reduction using NaBH_4_ in MeOH. This alcohol was chlorinated by concentrated HCl to furnish compound **5**, which was subjected to an electrochemical carboxylation under our above‐defined conditions. The final product (±)‐ibuprofen **6** was obtained in 52 % yield.

## Conclusions

In conclusion, we show that the direct electrochemical carboxylation of organic halides by the magnesium sacrificial anode process constitutes a reliable and efficient alternative to more classical chemical processes. These conditions indeed present some advantages like the use of a safe carbonyl source (CO_2_), no need for a catalyst, no necessity to use a metal reductant, mild and useful reaction, short reaction times and access to a broad panel of carboxylic acids. Insights into the reaction mechanism allowed us to determine that the reaction proceeds via an anionic route. Comparative experiments indicated that the nucleophilic species is not a classic organomagnesium reagent but an anionic species with selective reactivity, potentially a kind of organomagnesiate. The synthetic potential of our procedure was demonstrated by preparation of the anti‐inflammatory drug ibuprofen from 4’‐isobutylacetophenone using the electrochemical carboxylation as the key step.

## Experimental Section

### Materials and Methods for the Synthesis

Solvents and reagents were purchased from commercial suppliers and used without further purification. Melting points (mp) were measured on a Büchi B‐545 apparatus. ^1^H, ^13^C NMR and ^19^ F NMR spectra were recorded on a Brüker Avance II 400 spectrometer (^1^H: 400 MHz, ^13^C: 100 MHz, ^19^F: 376 MHz). Chemical shifts (δ) for ^1^H, ^13^C and ^19^F NMR spectra are reported in parts per million (ppm) relative to the residual solvent signal. Coupling constant values (*J*) are given in Hertz (Hz) and refer to apparent multiplicities, indicated as follows: s (singlet), d (doublet), dd (doublet of doublet), dt (doublet of triplet), dq (doublet of quartet), t (triplet), td (triplet of doublet), q (quartet), m (multiplet), hept (heptuplet). Compounds that have been previously described in the literature are linked to the corresponding bibliographic reference and their CAS registry number.

### General Procedure for Electrochemical Carboxylation of Aryl, Alkyl and Benzylic halides with CO_2_

To a 25 mL undivided electrochemical cell, fitted by a magnesium anode (ø: 8 mm) surrounded by a nickel foam as the cathode (area: 28 cm2, Goodfellow, porosity 500 μm) were added N,N’‐dimethylformamide (20 mL), tetrabutylammonium tetrafluoroborate (1.1 g) and 1,2‐dibromoethane (350 μL, 4 mmol). Then the mixture was taken degassed under vacuum and backfilled with argon. First, the mixture is electrolyzed at a constant current intensity of 0.32 A for 30 minutes at −10 °C. Then the electric current is stopped and was added the substrate (8 mmol). One more time, the mixture was degassed under vacuum and backfilled with CO_2_ gas twice (each lasts for 1 min). The solution is electrolyzed under continuous CO_2_ bubbling at 0.32 A for 2 to 5 h. A 3 M HCl aqueous solution (50 mL) was added to the mixture and the resulting solution extracted with ethyl acetate (3×50 mL). The combined organic layers were washed with H_2_O (2 x 100 mL), then with a saturated NaCl aqueous solution (100 mL), dried over Na_2_SO_4_, filtered and evaporated under vacuum. The crude residue was purified by silica gel column chromatography by using dichloromethane/EtOAc as the mixture of eluents to afford the carboxylic acid.

### Synthesis of Ibuprofen by Electrochemical Carboxylation with CO_2_

#### 1‐Chloro‐1‐(4‐isobutylphenyl)ethane (5)

In a 50‐mL round bottomed flask was dissolved *p*‐isobutylacetophenone (4.13 mL) in of methanol (12 mL). NaBH_4_ (1.04 g) was then added and the mixture stirred for 40 min at room temperature. 10 % HCl aqueous solution was then added to the mixture. The product was extracted from the solution using petroleum ether (3 x 20 mL), the combined organic layers dried over Na2SO4 and evaporated under vacuum to give the crude 1‐(4‐isobutylphenyl)ethanol as an oil.

To 1‐(4‐isobutylphenyl)ethanol previously obtained was added a 12 M HCl solution (40 mL) and the solution stirred for 8 min. The product was extracted from the mixture with petroleum ether (3 x 20 mL), the combined organic layers dried over Na2SO4 and evaporated under vacuum to give 1‐chloro‐1‐(4‐isobutylphenyl)ethane **5** as an oil.

#### 2‐(4‐Isobutylphenyl)propanoic acid (6)

To a 25 mL undivided electrochemical cell, fitted by a magnesium anode (ø: 8 mm) surrounded by a nickel foam as the cathode (area: 28 cm^2^, Goodfellow, porosity 500 μm) were added N,N’‐dimethylformamide (20 mL), tetrabutylammonium tetrafluoroborate (1.1 g) and 1,2‐dibromoethane (350 μL, 4 mmol). Then the mixture was degassed under vacuum and backfilled with argon. The mixture is first electrolyzed at a constant current intensity of 0.32 A for 30 minutes at −10 °C. Then the current is stopped and 1‐chloro‐1‐(4‐isobutylphenyl)ethane (1.61 mL, 8 mmol) was added. The mixture was degassed under vacuum and backfilled with CO_2_ gas twice (each lasts for 1 min) and the solution electrolyzed under continuous CO_2_ bubbling at 0.32 A for 200 min. A 3 M HCl aqueous solution (50 mL) was added to the mixture and the resulting solution extracted with ethyl acetate (3 x 50 mL). The combined organic layers were washed with H_2_O (2 x 100 mL), then with a saturated NaCl aqueous solution (100 mL), dried over Na_2_SO_4_, filtered and evaporated under vacuum. The crude residue was purified by silica gel column chromatography by using petroleum ether/EtOAc (100 % petroleum ether then 90/10) as the mixture of eluents to afford 2‐(4‐isobutylphenyl)propanoic acid **6**.

### Cyclic Voltamperometry

Cyclic voltammetries were carried out on a Metrohm Autolab PGSTAT204 workstation. In a 30 mL three‐necked electrochemical cell fitted with a vitreous carbon (VC) disk working electrode (Ø=3 mm), gold wire counter electrode and Ag/AgCl reference electrode were added DMF (20 mL) and NBu_4_BF_4_ (0.3 M) as supporting electrolyte. The solution was deoxygenated by bubbling with argon for 5 minutes under stirring before the addition of the substrates (0.05 M). Measurements were carried out at room temperature without stirring at a scan rate of 50 mV/s using an applied potential ranging from 0 to 2.5 V (oxidative scans) or from 0 to −3.5 V (reductive scans). Neutral atmosphere is maintained by letting argon above the solution during measurements. After each measurement, the working electrode was polished with an abrasive disk (type M, Ø=200 mm, P2400).

## Supporting Information Summary

See the Supporting Information for general procedures of the electrochemical syntheses and cyclic voltamperometry, as well as characterization of compounds data and NMR spectra. Additional references have been cited within the Supporting Information.

## Conflict of Interests

The authors declare no conflict of interest.

## Supporting information

As a service to our authors and readers, this journal provides supporting information supplied by the authors. Such materials are peer reviewed and may be re‐organized for online delivery, but are not copy‐edited or typeset. Technical support issues arising from supporting information (other than missing files) should be addressed to the authors.

Supporting Information

## Data Availability

The data that support the findings of this study are available in the supplementary material of this article.
