# Supplementary material for: Transition Metal‐Free Direct Electrochemical Carboxylation of Organic Halides Using a Sacrificial Magnesium Anode: Straightforward Synthesis of Carboxylic Acids
Source: ChemistryOpen. 2025 Jan 28;14(7):e202400426. doi: 10.1002/open.202400426 (PMC13042565; doi:10.1002/open.202400426)
Supplement: Supplementary file 1 — Supporting Information [file OPEN-14-e202400426-s001.pdf]

ChemistryOpen

Supporting Information

Transition Metal-Free Direct Electrochemical Carboxylation of Organic Halides Using a Sacrificial Magnesium Anode: Straightforward Synthesis of Carboxylic Acids

Iryna Lesko, Stéphane Sengmany,* Raphaël Beltran, Erwan Le Gall, and Eric Léonel*

Transition metal-free Direct Electrochemical Carboxylation of Organic Halides Using a Sacrificial Magnesium Anode: Straightforward Synthesis of Carboxylic Acids

Iryna Lesko,[a] Stéphane Sengmany,*[a] Raphaël Beltran,[b] Erwan Le Gall,[a] Eric Léonel*[a]

[a] I. Lesko, Dr. S. Sengmany, Prof. E. Le Gall, Prof. E. Léonel. Univ Paris Est Creteil, CNRS, ICMPE, UMR 7182, 2 rue Henri Dunant, 94320 Thiais, France

*E-mail: stephane.sengmany@cnrs.fr; eric.leonel@u-pec.fr

[b] Dr. R. Beltran, Sanofi, 45 Chemin de Meteline, 04200 Sisteron, France

Contents

Materials and methods for the synthesis……………….…………………..…….3

1. **General procedure for electrochemical carboxylation of aryl, alkyl and benzylic halides with CO_2……………………………………………………………..…3-4**
2. **Synthesis of ibuprofen by electrochemical carboxylation with CO_2…………….5-6**
3. **Characterization of compounds 2a-z ….……………………………………....7-12**
4. **Characterization of compounds 4 and 6……………………………….……...13**
5. **Bibliographic references list……….……………………………………………..14**
6. **^{1}H, ^{13}C and ^{19}F-NMR spectra…………………………..………..………15-75**

Cyclic voltamperometry ………..…...……………………………...….76-78

Materials and methods for the synthesis

Solvents and reagents were purchased from commercial suppliers and used without further purification. Melting points (mp) were measured on a Büchi B-545 apparatus. ^{1}H, ^{13}C NMR and ^{19}F NMR spectra were recorded on a Brüker Avance II 400 spectrometer (^{1}H: 400 MHz, ^{13}C: 100 MHz, ^{19}F: 376 MHz). Chemical shifts (δ) for ^{1}H, ^{13}C and ^{19}F NMR spectra are reported in parts per million (ppm) relative to the residual solvent signal. Coupling constant values (*J*) are given in Hertz (Hz) and refer to apparent multiplicities, indicated as follows: s (singlet), d (doublet), dd (doublet of doublet), dt (doublet of triplet), dq (doublet of quartet), t (triplet), td (triplet of doublet), q (quartet), m (multiplet), hept (heptuplet). Compounds that have been previously described in the literature are linked to the corresponding bibliographic reference and their CAS registry number.

1. General procedure for electrochemical carboxylation of aryl, alkyl and benzylic halides with CO_2

To a 25 mL undivided electrochemical cell, fitted by a magnesium anode (ø: 8 mm) surrounded by a nickel foam as the cathode (area: 28 cm^2, Goodfellow, porosity 500 µm) were added N,N'-dimethylformamide (20 mL), tetrabutylammonium tetrafluoroborate (1.1 g) and 1,2-dibromoethane (350 µL, 4 mmol). Then the mixture was taken degassed under vacuum and backfilled with argon. First, the mixture is electrolyzed at a constant current intensity of 0.32 A for 30 minutes at -10 °C. Then the electric current is stopped and was added the substrate (8 mmol). One more time, the mixture was degassed under vacuum and backfilled with CO_2 gas twice (each lasts for 1 min). The solution is electrolyzed under continuous CO_2 bubbling at 0.32 A for 2 to 5 h. A 3 M HCl aqueous solution (50 mL) was added to the mixture and the resulting solution extracted with ethyl acetate (3 x 50 mL). The combined organic layers were washed with H_2O (2 x 100 mL), then with a saturated NaCl aqueous solution (100 mL), dried over Na_2SO_4, filtered and evaporated under vacuum. The crude residue was purified by silica gel column chromatography by using dichloromethane/EtOAc as the mixture of eluents to afford the carboxylic acid.

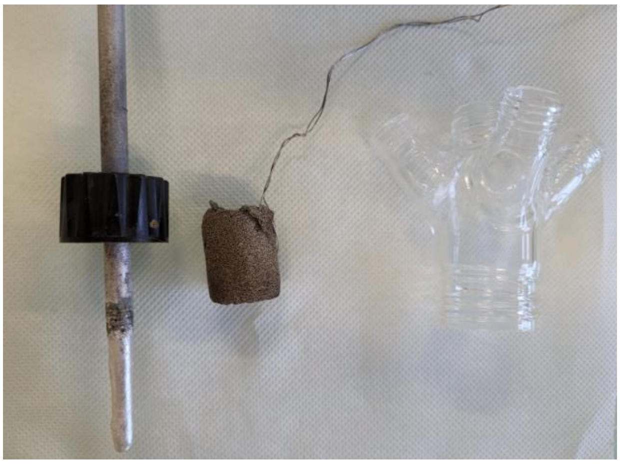

Figure 1. Electrochemical materials composed of the anode, the cathode and the cell (from left to right)

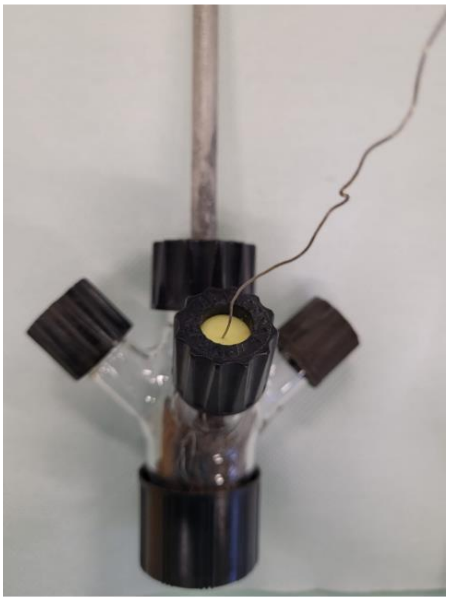

Figure 2. Assembled electrochemical undivided electrochemical cell fitted with a magnesium anode surrounded by a nickel

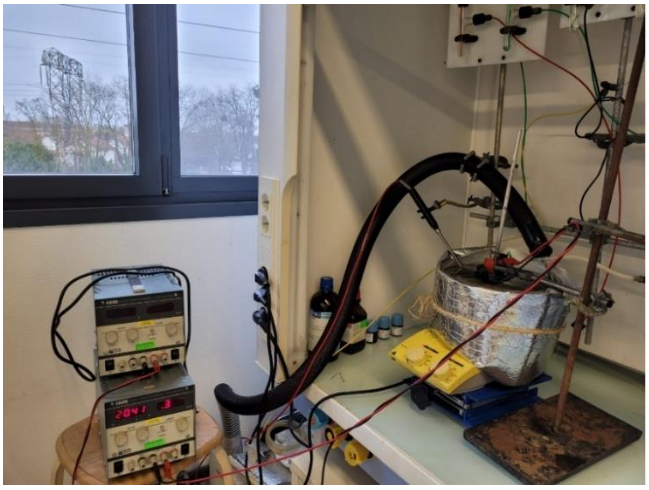

Figure 3. Electrochemical assembly

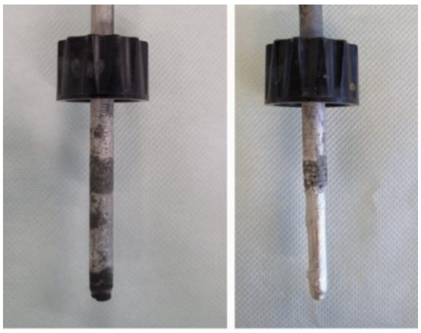

Figure 4. Sacrificial magnesium anode: before reaction (at the left) and after reaction (at the right)

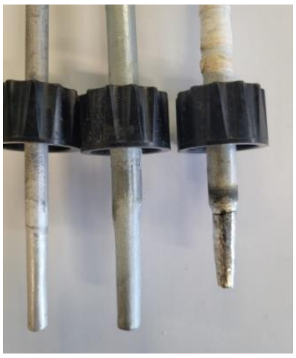

Figure 5. various sacrificial anode: Mg, Zn and Al (from left to right)

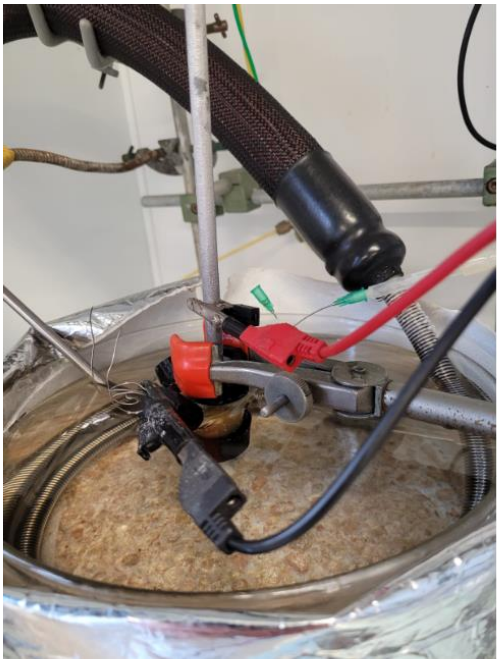

Figure 6. Electrochemical assembly: undivided electrochemical cell with continuous CO_2 bubbling immersed in ethanol cooled bath at -10 °C

2. Synthesis of ibuprofen by electrochemical carboxylation with CO_2

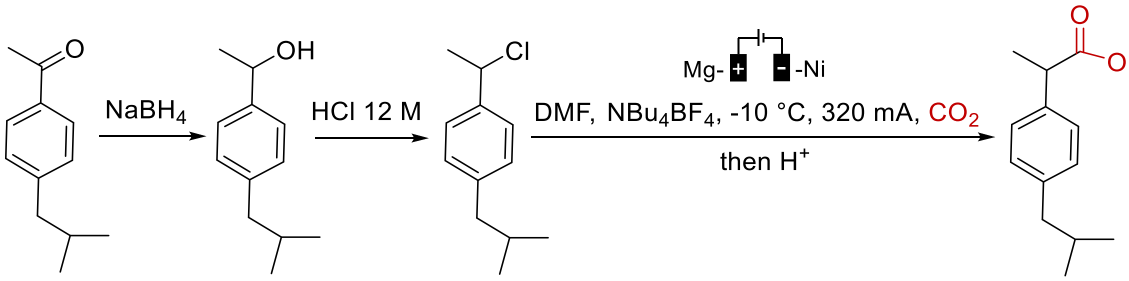

3 4 5 6

1-Chloro-1-(4-isobutylphenyl)ethane (**5**). In a 50-mL round bottomed flask was dissolved *p*-isobutylacetophenone (4.13 mL) in of methanol (12 mL). $NaBH_4$ (1.04 g) was then added and the mixture stirred for 40 min at room temperature. 10% HCl aqueous solution was then added to the mixture. The product was extracted from the solution using petroleum ether (3 x 20 mL), the combined organic layers dried over Na_2SO_4 and evaporated under vacuum to give the crude 1-(4-isobutylphenyl)ethanol as an oil.

To 1-(4-isobutylphenyl)ethanol previously obtained was added a 12 M HCl solution (40 mL) and the solution stirred for 8 min. The product was extracted from the mixture with petroleum ether (3 x 20 mL), the combined organic layers dried over Na_2SO_4 and evaporated under vacuum to give 1-chloro-1-(4-isobutylphenyl)ethane **5** as an oil.

2-(4-Isobutylphenyl)propanoic acid (6). To a 25 mL undivided electrochemical cell, fitted by a magnesium anode (ø: 8 mm) surrounded by a nickel foam as the cathode (area: 28 cm^2, Goodfellow, porosity 500 µm) were added N,N'-dimethylformamide (20 mL), tetrabutylammonium tetrafluoroborate (1.1 g) and 1,2-dibromoethane (350 µL, 4 mmol). Then the mixture was degassed under vacuum and backfilled with argon. The mixture is first electrolyzed at a constant current intensity of 0.32 A for 30 minutes at -10 °C. Then the current is stopped and 1-chloro-1-(4-isobutylphenyl)ethane (1.61 mL, 8 mmol) was added. The mixture was degassed under vacuum and backfilled with CO_2 gas twice (each lasts for 1 min) and the solution electrolyzed under continuous CO_2 bubbling at 0.32 A for 200 min. A 3 M HCl aqueous solution (50 mL) was added to the mixture and the resulting solution extracted with ethyl acetate (3 x 50 mL). The combined organic layers were washed with H_2O (2 x 100 mL), then with a saturated NaCl aqueous solution (100 mL), dried over Na_2SO_4, filtered and evaporated under

vacuum. The crude residue was purified by silica gel column chromatography by using petroleum ether/EtOAc (100% petroleum ether then 90/10) as the mixture of eluents to afford 2-(4-isobutylphenyl)propanoic acid **6**.

3. Characterization of compounds 2a-z

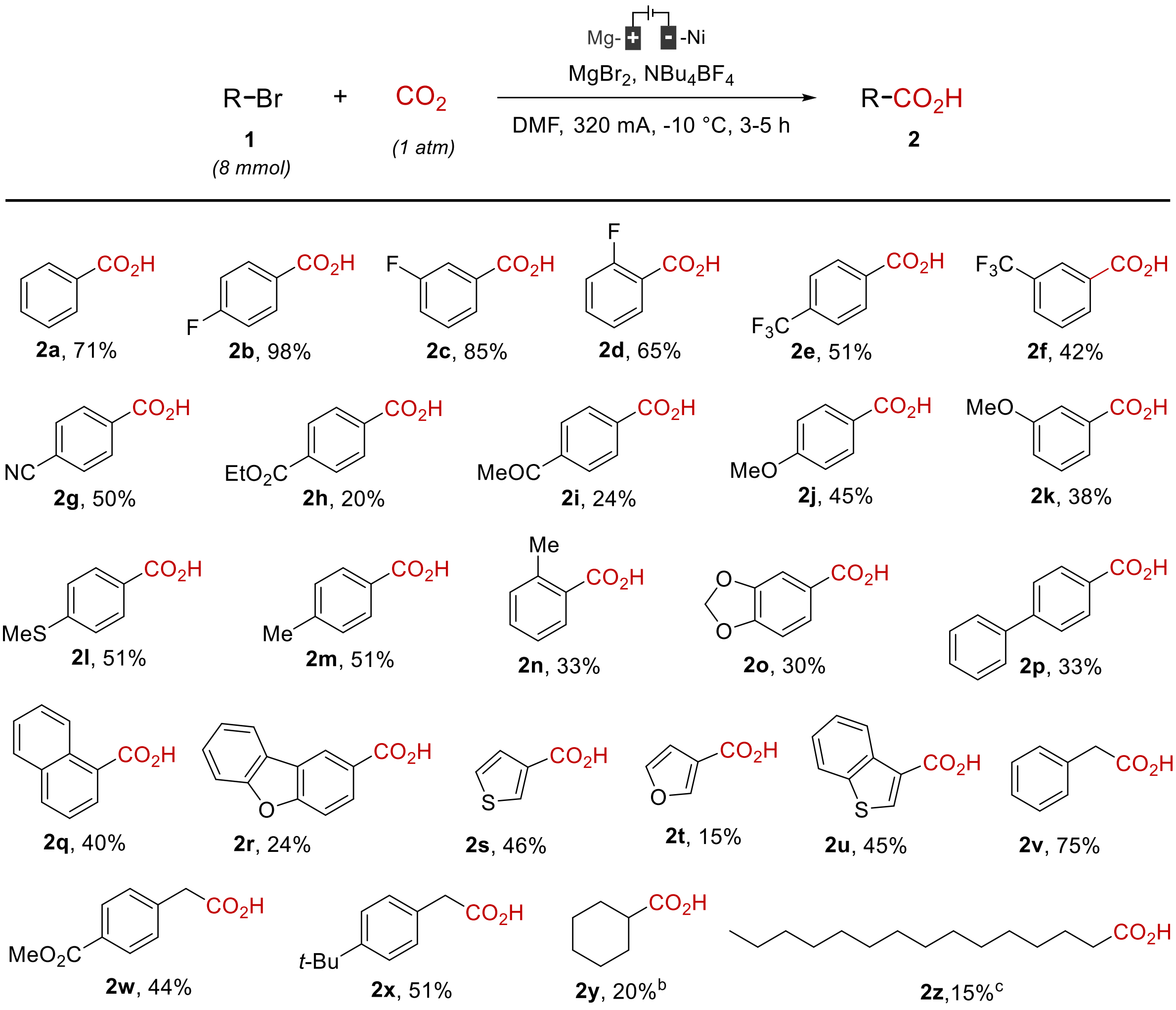

Benzoic acid (2a); CAS RN: 65-85-0[1]

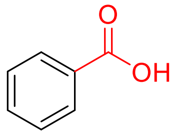

White solid. Yield = 71% (m = 0.690 g)

Melting point: 121 °C

^{1}H NMR (400 MHz, $CDCl_3$): δ 8.13 (d, *J* = 7.9 Hz, 2H), 7.63 (t, *J* = 7.4 Hz, 1H), 7.49 (t, *J* = 6.9 Hz, 2H).

^{13}C NMR (100 MHz, $CDCl_3$): δ 172.3, 133.9, 130.3, 129.3, 128.5.

4-Fluorobenzoic acid (2b); CAS RN: 456-22-4[2]

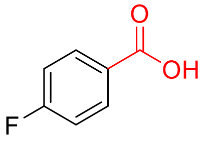

White solid. Yield = 98% (m = 1.098 g)

Melting point: 182 °C

^{1}H NMR (400 MHz, DMSO-d_6): δ 13.04 (s, 1H), 8.00 (t, *J* = 6.4 Hz, 2H), 7.32 (t, *J* = 8.6 Hz, 2H).
^{13}C NMR (100 MHz, DMSO-d_6): δ 166.9, 165.4 (d, *J* = 250.6 Hz), 132.6 (d, *J* = 9.5 Hz), 127.8 (d, *J* = 2.7 Hz), 116.1 (d, *J* = 22.1 Hz).

^{19}F NMR (376 MHz, DMSO): δ -106.89.

3-Fluorobenzoic acid (2c); CAS RN: 455-38-9[2]

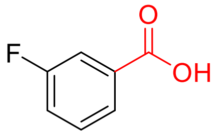

White solid. Yield = 85% (m = 0.950 g)

Melting point: 112 °C

^{1}H NMR (400 MHz, DMSO-d_6): δ 13.29 (s, 1H), 7.78 (d, *J* = 6.8 Hz, 1H), 7.65 (d, *J* = 10.6 Hz, 1H), 7.58-7.53 (m, 1H), 7.50-7.46 (m, 1H).

^{13}C NMR (100 MHz, DMSO-d_6): δ 166.7 (d, *J* = 2.9 Hz), 162.4 (d, *J* = 244.7 Hz), 133.7 (d, *J* = 7.2 Hz), 131.3 (d, *J* = 8.0 Hz), 125.9 (d, *J* = 2.9 Hz), 120.3 (d, *J* = 21.2 Hz), 116.2 (d, *J* = 22.6 Hz).

^{19}F NMR (376 MHz, DMSO-d_6): δ -112.58.

2-Fluorobenzoic acid (2d); CAS RN: 445-29-4[3]

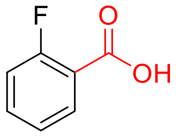

White solid. Yield = 65% (m = 0.728 g)

Melting point: 123 °C

^{1}H NMR (400 MHz, DMSO-d_6): δ 13.26 (s, 1H), 7.87 (t, *J* = 7.8 Hz, 1H), 7.64 (q, *J* = 7.2 Hz, 1H), 7.45–7.20 (m, 2H).

^{13}C NMR (100 MHz, DMSO-d_6): δ 165.5 (d, *J* = 3.0 Hz), 161.6 (d, *J* = 256.8 Hz), 135.2 (d, *J* = 9.0 Hz), 132.4, 124.9 (d, *J* = 3.9 Hz), 119.8 (d, *J* = 10.0 Hz), 117.4 (d, *J* = 22.2 Hz).

^{19}F NMR (376 MHz, DMSO-d_6): δ -110.62.

4-(Trifluoromethyl)benzoic acid (2e); CAS RN: 455-24-3[4]

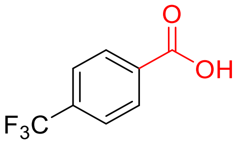

White solid. Yield = 51% (m = 0.769 g)

Melting point: 212 °C

^{1}H NMR (400 MHz, DMSO-d_6): δ 13.49 (s, 1H), 8.11 (d, *J* = 7.9 Hz, 2H), 7.80 (d, *J* = 8.0 Hz, 2H).

^{13}C NMR (100 MHz, DMSO-d_6): δ 166.7, 135.1, 133.0 (q, *J* = 31.9 Hz), 130.6, 126.1 (q, *J* = 4.5, 3.9 Hz), 124.3 (q, *J* = 273.1 Hz).

^{19}F NMR (376 MHz, $CDCl_3$): δ -61.80.

3-(Trifluoromethyl)benzoic acid (2f); CAS RN: 454-92-2[4]

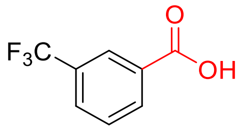

White solid. Yield = 42% (m = 0.630 g)

Melting point: 100 °C

^{1}H NMR (400 MHz, DMSO-d_6): δ 13.52 (s, 1H), 8.22 (d, J = 7.8 Hz, 2H), 8.17 (s, 2H), 7.98 (d, J = 7.8 Hz, 2H), 7.75 (t, J = 7.8 Hz, 2H).

^{13}C NMR (100 MHz, DMSO-d_6): δ 166.5, 133.6, 132.4, 130.4, 130.6–130.5(m) 129.9 (q, J = 32.0 Hz), 126.0 (q, J = 4.0 Hz), 124.2 (q, J = 272.3 Hz).

^{19}F NMR (376 MHz, $CDCl_3$): δ -61.55.

4-Cyanobenzoic acid (2g); CAS RN: 619-65-8[2]

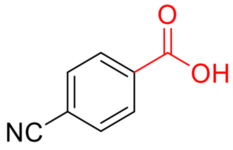

White solid. Yield = 50% (m = 0.603 g)

Melting point: 212 °C

^{1}H NMR (400 MHz, DMSO-d_6): δ 13.60 (s, 1H), 8.08 (d, J = 6.9 Hz, 2H), 7.98 (d, J = 6.9 Hz, 2H).

^{13}C NMR (100 MHz, DMSO-d_6): δ 166.6, 135.4, 133.2 (2C), 130.40 (2C), 118.68, 115.52.

4-Ethoxycarbonyl)benzoic acid (2h); CAS RN: 713-57-5[1]

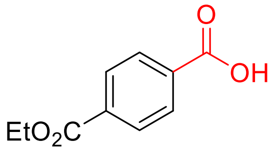

White solid. Yield = 20% (m = 0.310 g)

Melting point: 169 °C

^{1}H NMR (400 MHz, $CDCl_3$): δ 11.92 (s, 1H), 8.21 (d, J = 8.0 Hz, 2H), 8.18 (d, J = 7.7 Hz, 2H), 4.45 (q, J = 7.1 Hz, 2H), 1.45 (t, J = 6.9 Hz, 3H).

^{13}C NMR (100 MHz, $CDCl_3$): δ 170.9, 165.7, 135.1, 132.8, 130.2, 129.7, 61.6, 14.3.

4-Acetylbenzoic acid (2i); CAS RN: 586-89-0[2]

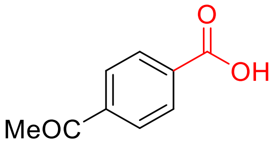

White solid. Yield = 24% (m = 0.387 g)

Melting point: 209 °C

^{1}H NMR (400 MHz, DMSO-d_6): δ 13.38 (s, 1H), 8.09–8.04 (m, 4H), 2.64 (s, 3H).

^{13}C NMR (100 MHz, DMSO-d_6): δ 198.2, 167.1, 140.3, 135.0, 130.0 (2C), 128.8 (2C), 27.5.

4-Methoxybenzoic acid (2j); CAS RN: 100-09-4[1]

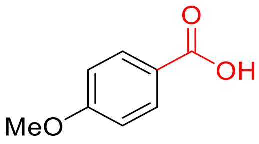

White solid. Yield = 45% (m = 0.637 g)

Melting point:183 °C

^{1}H NMR (400 MHz, DMSO-d_6): δ 12.66 (s, 1H), 7.91 (d, J = 8.3 Hz, 2H), 7.02 (d, J = 8.3 Hz, 2H), 3.83 (s, 3H).

^{13}C NMR (100 MHz, DMSO-d_6): δ 167.5, 163.3, 131.8 (2C), 123.4, 114.2 (2C), 55.8.

4-Methoxybenzoic acid (2k); CAS RN: 586-38-9[1]

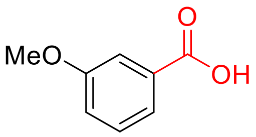

White solid. Yield = 38% (m = 0.452 g)

Melting point: 103 °C

^{1}H NMR (400 MHz, $CDCl_3$): δ 11.74 (s, 1H), 7.76 (d, *J* = 7.6 Hz, 1H), 7.66 (s, 1H), 7.42 (t, *J* = 7.9 Hz, 1H), 7.19 (d, *J* = 8.1 Hz, 1H), 3.90 (s, 3H).

^{13}C NMR (100 MHz, $CDCl_3$): δ 172.4, 159.6, 130.6, 129.6, 122.7, 120.5, 114.4, 55.4.

4-(Methylthio)benzoic acid (2l); CAS RN: 13205-48-6[1]

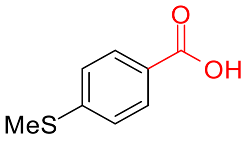

White solid. Yield = 51% (m = 0.680 g)

Melting point: 187 °C

^{1}H NMR (400 MHz, DMSO-d_6): δ 12.87 (s, 1H), 7.87 (d, *J* = 8.0 Hz, 2H), 7.31 (d, *J* = 8.1 Hz, 2H), 2.50 (s, 3H).

^{13}C NMR (100 MHz, DMSO-d_6): δ 167.6, 145.3, 130.2 (2C), 127.1, 125.30(2C), 14.4.

4-Methylbenzoic acid (2m); CAS RN: 99-94-5[4]

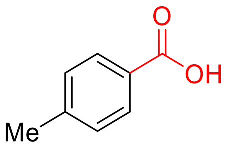

White solid. Yield = 51% (m = 0.677 g)

Melting point: 170 °C

^{1}H NMR (400 MHz, $CDCl_3$): δ 10.77 (s, 1H), 8.05 (d, *J* = 7.8 Hz, 2H), 7.31 (d, *J* = 7.9 Hz, 2H), 2.47 (s, 3H).

^{13}C NMR (100 MHz, $CDCl_3$): δ 172.5, 144.7, 130.3, 129.3, 126.6, 21.8.

2-Methylbenzoic acid (2n); CAS RN: 118-90-1[4]

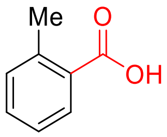

White solid. Yield = 33% (m = 0.360 g)

Melting point: 169 °C

^{1}H NMR (400 MHz, DMSO-d_6): δ 12.83 (s, 1H), 7.84 (d, *J* = 7.7 Hz, 1H), 7.46 (t, *J* = 7.5 Hz, 1H), 7.31 (d, *J* = 8.0 Hz, 2H), 2.54 (s, 3H).

^{13}C NMR (100 MHz, DMSO-d_6): δ 169.1, 139.5, 132.2, 132.0, 130.9, 130.6, 126.3, 21.7.

Benzo[*d*][1,3]dioxole-5-carboxylic acid (2o); CAS RN: 94-53-1[4]

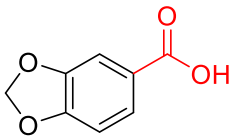

White solid. Yield = 30% (m = 0.396 g)

Melting point: 225 °C

^{1}H NMR (400 MHz, DMSO-d_6): δ 12.82 (s, 1H), 7.54 (d, *J* = 8.1 Hz, 1H), 7.35 (s, 1H), 7.00 (d, *J* = 8.1 Hz, 1H), 6.12 (s, 2H).

^{13}C NMR (100 MHz, DMSO-d_6): δ 167.1, 151.6, 147.9, 125.4, 125.1, 109.3, 108.5, 102.4.

[1,1'-Biphenyl]-4-carboxylic acid (2p); CAS RN: 92-92-2[1]

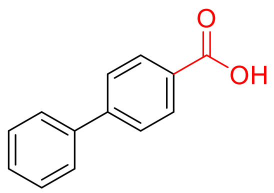

White solid. Yield = 33% (m = 0.522 g)

Melting point: 224 °C

^{1}H NMR (400 MHz, DMSO-d_6): δ 13.02 (s, 1H), 8.08–8.01 (m, 2H), 7.81 (d, J = 8.1 Hz, 2H), 7.78–7.72 (m, 2H), 7.52 (t, J = 7.8 Hz, 2H), 7.44 (t, J = 7.4 Hz, 1H).

^{13}C NMR (100 MHz, DMSO-d_6): δ 167.6, 144.8, 139.5, 130.4 (2C), 130.1, 129.6 (2C), 128.8, 127.4 (2C), 127.3 (2C).

1-Naphthoic acid (2q) ; CAS RN: 86-55-5[2]

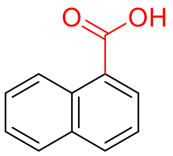

White solid. Yield = 40% (m = 0.550 g)

Melting point: 153 °C

^{1}H NMR (400 MHz, $CDCl_3$): δ 12.45 (s, 1H), 9.11 (d, J = 8.7 Hz, 1H), 8.44 (d, J = 7.3 Hz, 1H), 8.11 (d, J = 8.0 Hz, 1H), 7.93 (d, J = 8.1 Hz, 1H), 7.68 (t, J = 7.7 Hz, 1H), 7.57 (q, J = 7.2 Hz, 2H).

^{13}C NMR (100 MHz, $CDCl_3$): δ 173.6, 134.7, 134.0, 132.0, 131.7, 128.8, 128.2, 126.0, 125.6, 124.6.

Dibenzo[*b*,*d*]furan-2-carboxylic acid (2r); CAS RN: 22439-48-1[1]

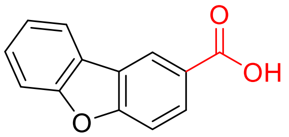

White solid. Yield = 24% (m = 0.400 g)

Melting point: 247 °C

^{1}H NMR (400 MHz, DMSO-d_6): δ 13.08 (d, J = 7.8 Hz, 1H), 8.79 (s, 1H), 8.31 (d, J = 7.5 Hz, 1H), 8.13 (d, J = 8.6 Hz, 1H), 7.78 (dd, J = 17.7, 8.5 Hz, 2H), 7.59 (t, J = 7.8 Hz, 1H), 7.46 (t, J = 7.5 Hz, 1H).

^{13}C NMR (100 MHz, DMSO-*d*6): δ 167.7, 158.4, 156.5, 129.5, 128.7, 126.5, 124.3, 124.1, 123.6 (2C), 122.2, 112.3, 112.1.

Thiophene-3-carboxylic acid (2s); CAS RN: 88-13-1[2]

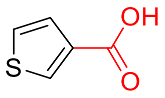

White solid. Yield = 46% (m = 0.472 g)

Melting point: 131°C

^{1}H NMR (400 MHz, DMSO-d_6): δ 12.75 (s, 1H), 8.26 (s, 1H), 7.60 (dd, J = 4.8, 2.9 Hz, 1H), 7.47–7.41 (m, 1H).

^{13}C NMR (100 MHz, DMSO-d_6): δ 164.1, 134.8, 133.8, 128.2, 127.7.

Furan-3-carboxylic acid (2t); CAS RN: 488-93-7[2]

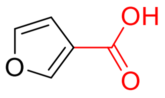

White solid. Yield = 15% (m = 0.137 g)

Melting Point: 118 °C

^{1}H NMR (400 MHz, DMSO-d_6): δ 12.68 (s, 1H), 8.30 (s, 1H), 7.78 (s, 1H), 6.74 (s, 1H).

^{13}C NMR (100 MHz, DMSO-d_6): δ 164.4, 148.6, 145.1, 120.2, 110.3.

Benzo[*b*]thiophene-3-carboxylic acid (2u); CAS RN: 5381-25-9[5]

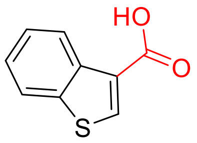

White solid. Yield = 45% (m = 0.641 g)

Melting point: 174 °C

^{1}H NMR (400 MHz, DMSO-d_6): δ 8.67 (d, *J* = 2.2 Hz, 1H), 8.53 (d, *J* = 7.9 Hz, 1H), 8.11 (d, *J* = 8.0 Hz, 1H), 7.50 (dt, *J* = 22.6, 7.4 Hz, 2H).

^{13}C NMR (100 MHz, DMSO-d_6): δ 164.3, 140.2, 138.5, 127.7, 125.8 (2C), 125.4, 124.7, 123.6.

2-Phenylacetic acid (2v); CAS RN: 103-82-2[6]

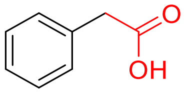

White solid. Yield = 75% (m = 0.817 g)

Melting point: 77 °C

^{1}H NMR (400 MHz, $CDCl_3$): δ 7.38–7.27 (m, 5H), 3.67 (s, 2H).

^{13}C NMR (100 MHz, $CDCl_3$): δ 177.9, 133.3, 129.4 (2C), 128.7 (2C), 127.4, 41.1.

2-(4-(Methoxycarbonyl)phenyl)acetic acid (2w); CAS RN: 22744-12-3[6]

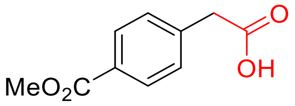

White solid. Yield = 44% (m = 0.682 g)

Melting point: 109 °C

^{1}H NMR (400 MHz, $CDCl_3$): δ 10.56 (s, 1H), 8.03 (d, *J* = 7.3 Hz, 2H), 7.38 (d, *J* = 7.5 Hz, 2H), 3.94 (s, 3H), 3.74 (s, 2H).

^{13}C NMR (100 MHz, $CDCl_3$): δ 177.0, 166.9, 138.3, 130.0 (2C), 129.5 (2C), 129.3, 52.2, 41.0.

2-(4-(*Tert*-butyl)phenyl)acetic acid (2x); CAS RN: 32857-63-9[6]

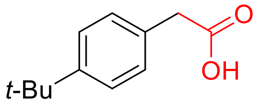

White solid. Yield = 51% (m = 0.782 g)

Melting point: 78 °C

^{1}H NMR (400 MHz, $CDCl_3$): δ 11.90 (s, 1H), 7.44 (d, *J* = 7.2 Hz, 2H), 7.30 (d, *J* = 7.7 Hz, 2H), 3.69 (s, 2H), 1.39 (s, 9H).

^{13}C NMR (100 MHz, $CDCl_3$): δ 178.7, 150.3, 130.3, 129.1 (2C), 125.7 (2C), 40.7, 34.6, 31.4 (3C).

Cyclohexanecarboxylic acid (2y); CAS RN: 98-89-5 [8]

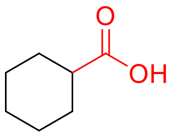

Colorless oil. Yield = 14% (m = 0.145 g)

^{1}H NMR (400 MHz, $CDCl_3$): δ 11.18 (s, 1H), 2.26 (dt, *J* = 11.2, 5.5 Hz, 1H), 1.86 (d, *J* = 12.6 Hz, 2H), 1.70-1.68 (m, 2H), 1.58 (d, *J* = 8.5 Hz, 1H), 1.38 (q, *J* = 12.1, 2H), 1.25–1.06 (m, 3H).

^{13}C NMR (100 MHz, $CDCl_3$): δ 182.9, 43.0, 28.8 (2C), 25.7, 25.3 (2C).

Pentadecanoic acid (2z); CAS RN: 1002-84-2[9]

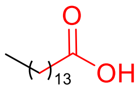

White solid. Yield = 15% (m = 0.283 g)

Melting point: 50 °C

^{1}H NMR (400 MHz, $CDCl_3$): δ 11.24 (s, 1H), 2.34 (t, *J* = 7.3 Hz, 2H), 1.66–1.59 (m, 2H), 1.25 (m, 22H), 0.87 (m, *J* = 6.9, 2.0 Hz, 3H).

^{13}C NMR (100 MHz, $CDCl_3$): δ 180.5, 34.2, 32.0, 29.7 (4C), 29.6, 29.5, 29.4, 29.3, 29.1, 24.7, 22.7, 14.1.

4. Characterization of compounds 5 and 6

1-Chloro-1-(4-isobutylphenyl)ethane (5) CAS RN: 62049-65-4[10]

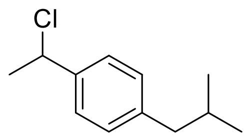

Colourless liquid. Yield = 82% (m = 2.293 g)

^{1}H NMR (400 MHz, $CDCl_3$): δ 7.46 (d, *J* = 8.0 Hz, 2H), 7.26 (d, *J* = 7.9 Hz, 2H), 5.22 (q, *J* = 6.8 Hz, 1H), 2.61 (d, *J* = 7.2 Hz, 2H), 2.02-1.96 (m, 4H), 1.05 (d, *J* = 6.6 Hz, 6H).

^{13}C NMR (100 MHz, $CDCl_3$): δ 142.0, 140.3, 129.5, 126.4, 59.0, 45.3, 30.4, 26.7, 22.6.

2-(4-Isobutylphenyl)propanoic acid (6); CAS RN: 15687-27-1[10]

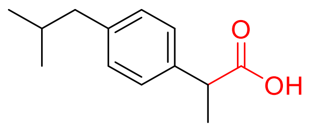

White solid. Yield = 52% (m = 0.861 g)

Melting point: 71 °C

^{1}H NMR (400 MHz, DMSO-d_6): δ 12.27 (s, 1H), 7.21 (d, *J* = 7.8 Hz, 2H), 7.13 (d, *J* = 7.5 Hz, 2H), 3.65 (q, *J* = 7.1 Hz, 1H), 2.44 (d, *J* = 7.2 Hz, 2H), 1.83 (hept, *J* = 6.7 Hz, 1H), 1.37 (d, *J* = 7.0 Hz, 3H), 0.88 (d, *J* = 6.3 Hz, 6H).

^{13}C NMR (100 MHz, DMSO-d_6): δ 176.0, 140.1, 139.0, 129.5, 127.6, 44.8, 44.7, 30.1, 22.7, 19.1.

5. Bibliographic references list

[1] Guo-Quan Sun *et al*., *Nat Commun*. **2021**, *12*, 7086-7095.

[2] Xianjin Zhu *et al.*, *Green Chem.***2020**, *22*, 4357-4363.

[3] Masayuki Kirihara *et al.*, *Synlett.*, **2022**, *33*, 1670-1674.

[4] Yanwei Wang *et al.*, *Chem. Commun.* **2020**, *56*, 14416-14419.

[5] Bence S. Nagy *et al.*, *Adv. Synth. Catal.***2022**, *364*, 1998-2008.

[6] Masachi Ohkoshi *et al.*, *Tetrahedron* **2010**, *66*, 7732-7737.

[7] Abir Sarbajna *et al.*, *ACS Catal.***2017**, *7*, 2786-2790.

[8] Hongwei Shi *et al.*, *Green Chem.* **2022**, *24*, 5835-5841.

[9] Rui Zhu *et al.*, *ACS Catal*. **2017**, *7*, 7520-7528.

[10] Albrecht Metzger *et al.*, *Angew. Chem. Int. Ed*. **2010**, *49*, 4665-4668.

^{1}H NMR

2a

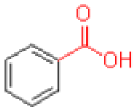

benzoic acid

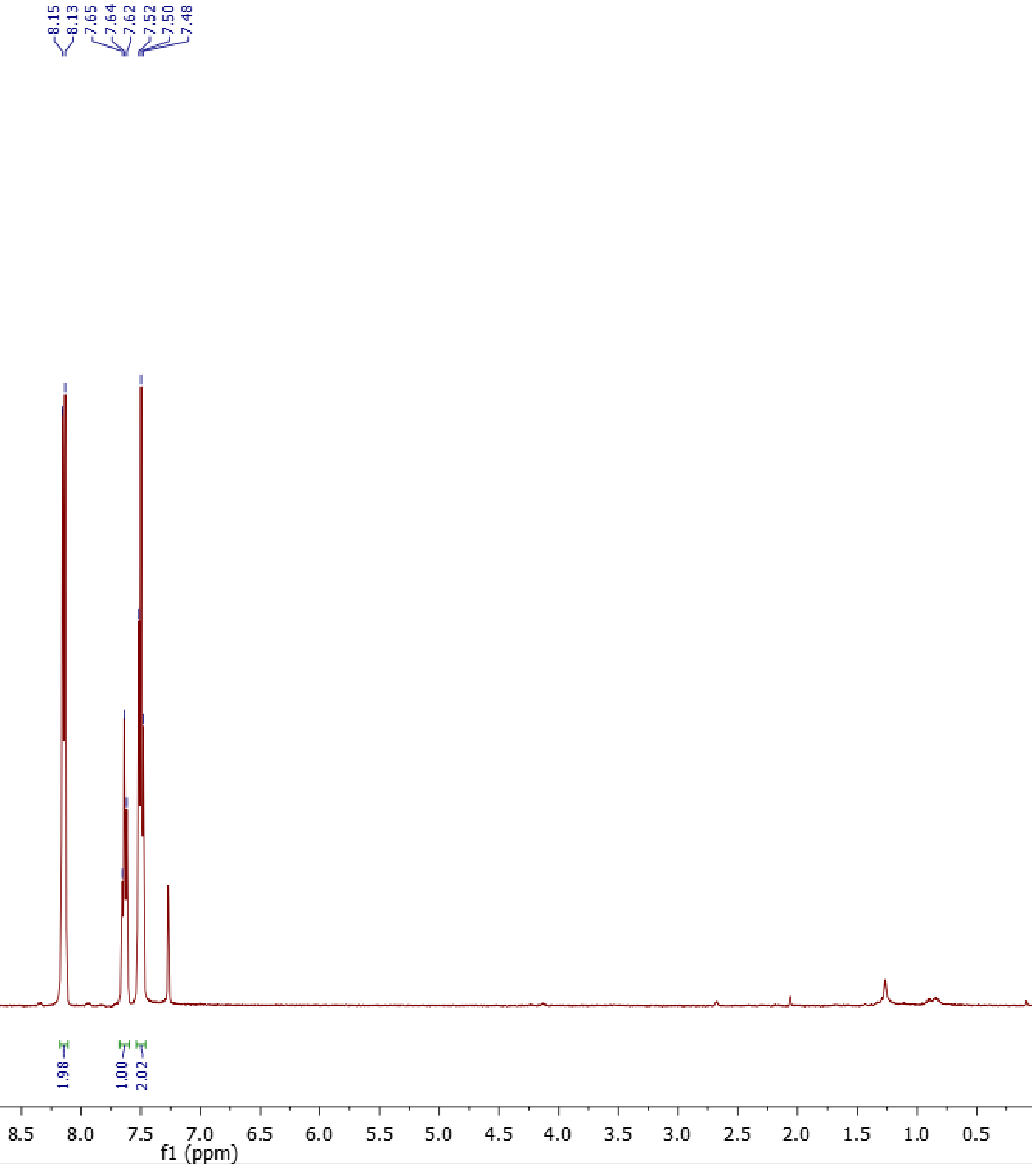

^{13}C NMR

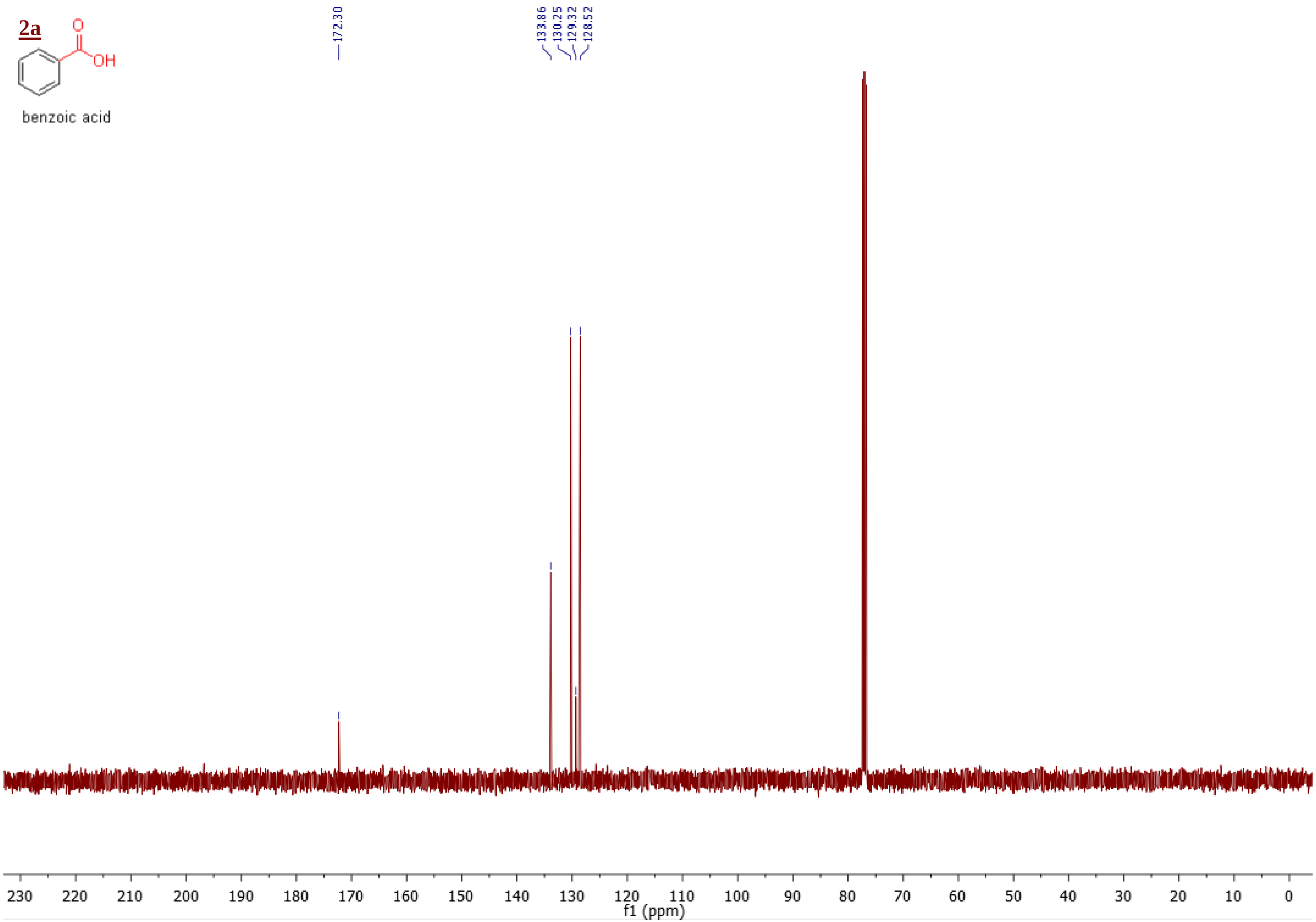
2a
OH
benzoic acid
172.30
133.86
130.25
129.32
128.52
230
220
210
200
190
180
170
160
150
140
130
120
110
100
90
80
70
60
50
40
30
20
10
0
f1 (ppm)

^{1}H NMR

2b

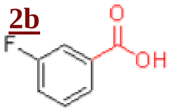

3-fluorobenzoic acid

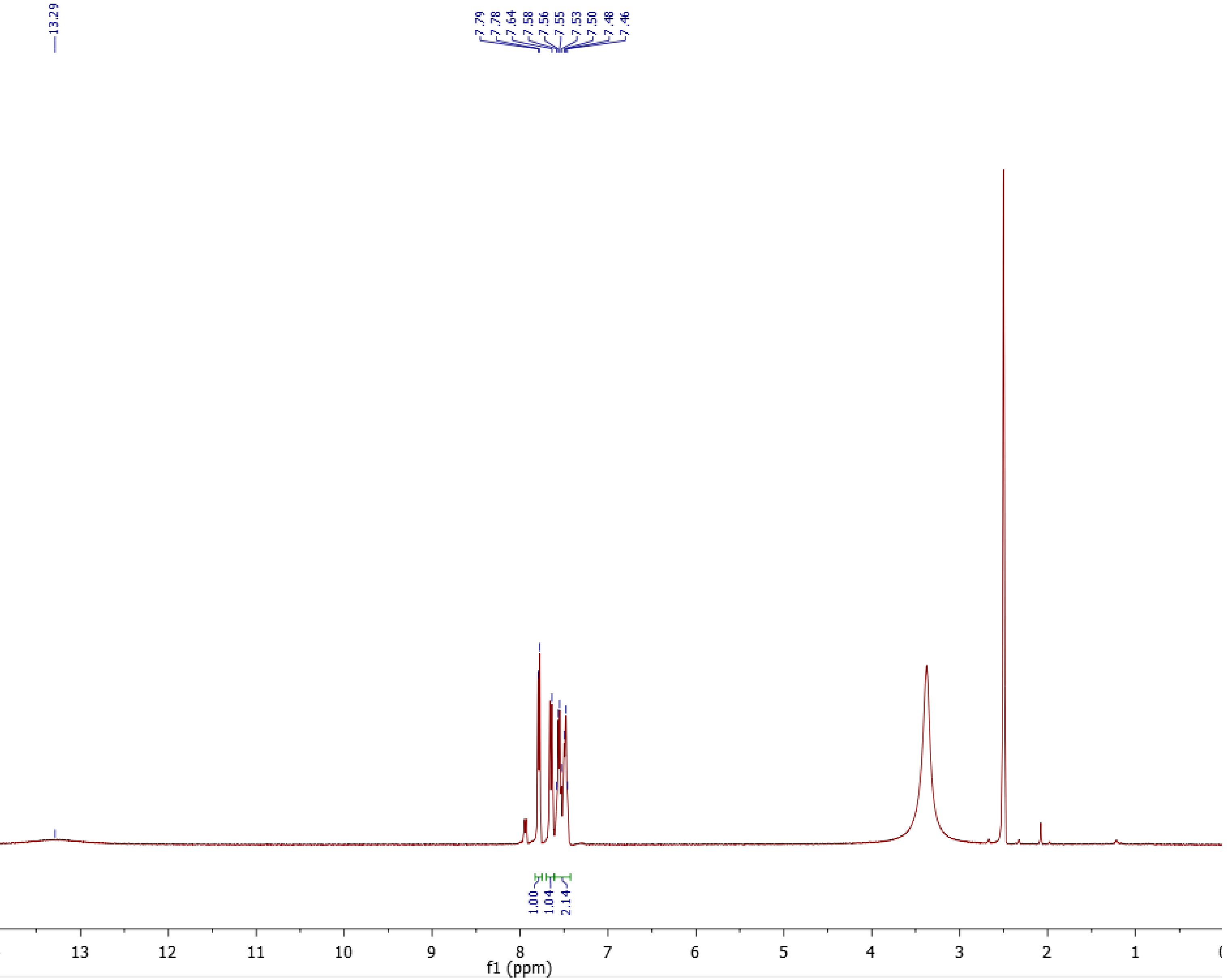

^{13}C NMR

2b

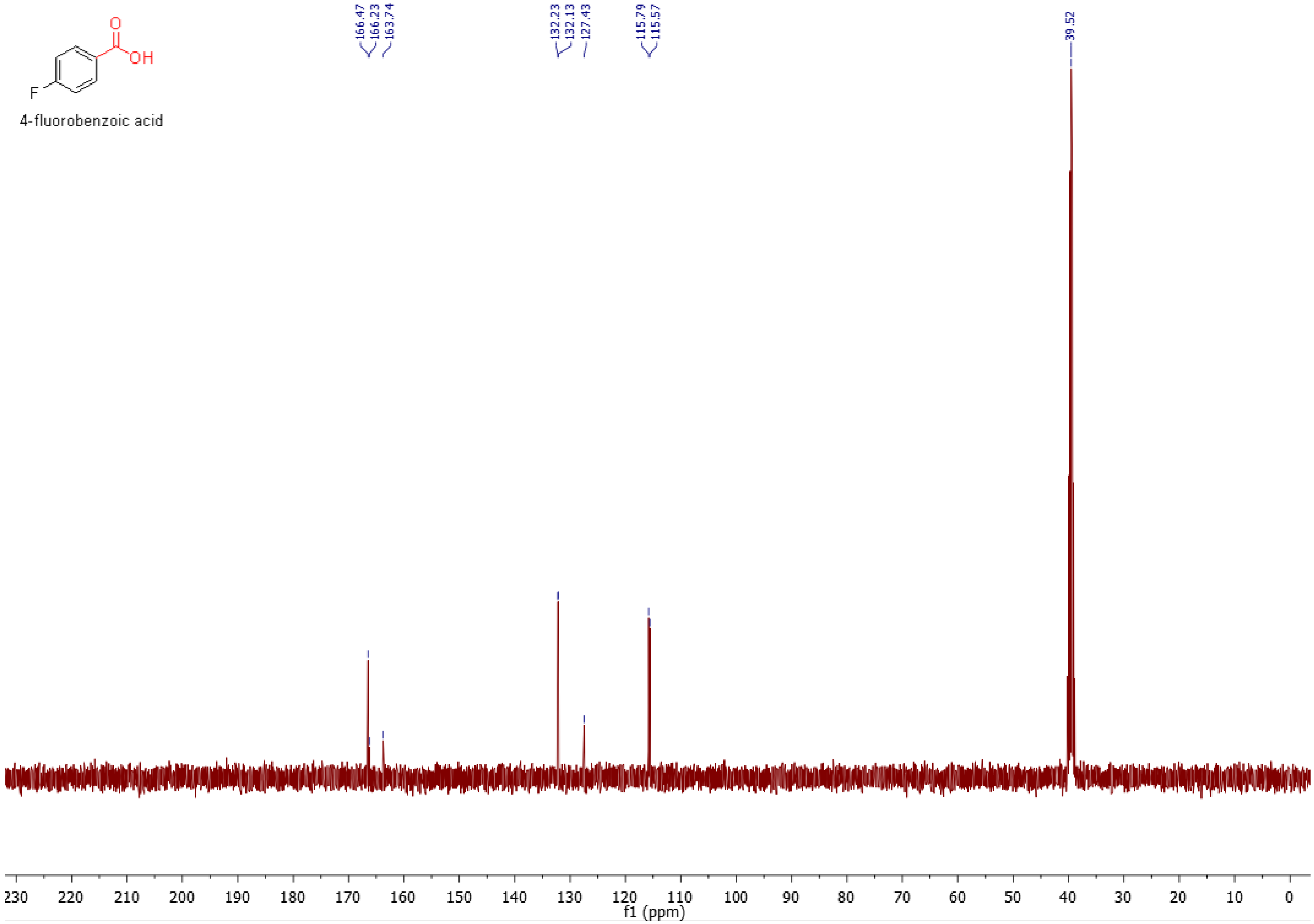
O
OH
F
4-fluorobenzoic acid
166.47
166.23
163.74
132.23
132.13
127.43
115.79
115.57
39.52
230
220
210
200
190
180
170
160
150
140
130
120
110
100
90
80
70
60
50
40
30
20
10
0
f1 (ppm)

^{19}F NMR

2b

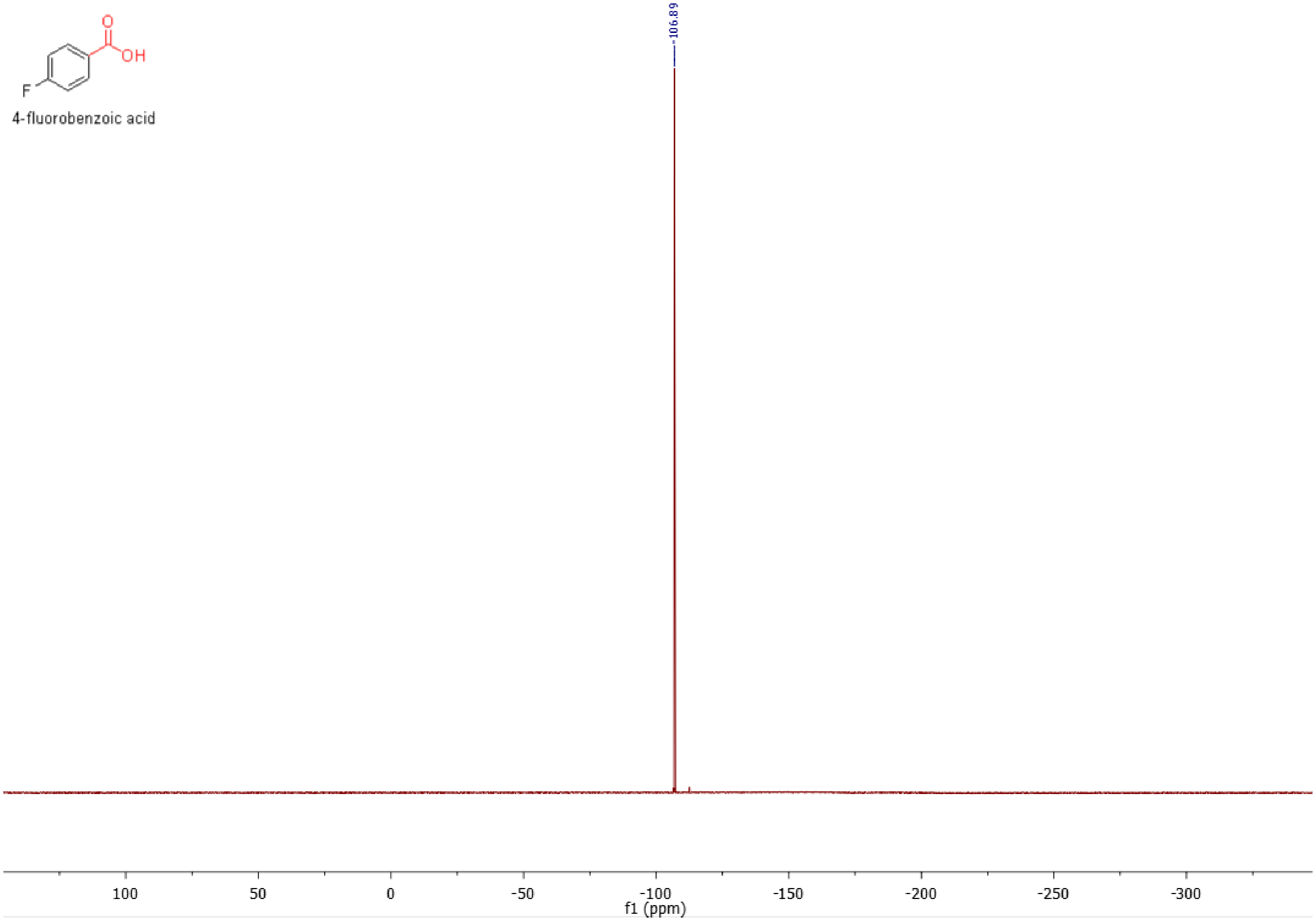

O
OH
F
4-fluorobenzoic acid
-106.89
100
50
0
-50
-100
-150
-200
-250
-300
f1 (ppm)

^{1}H NMR

2c

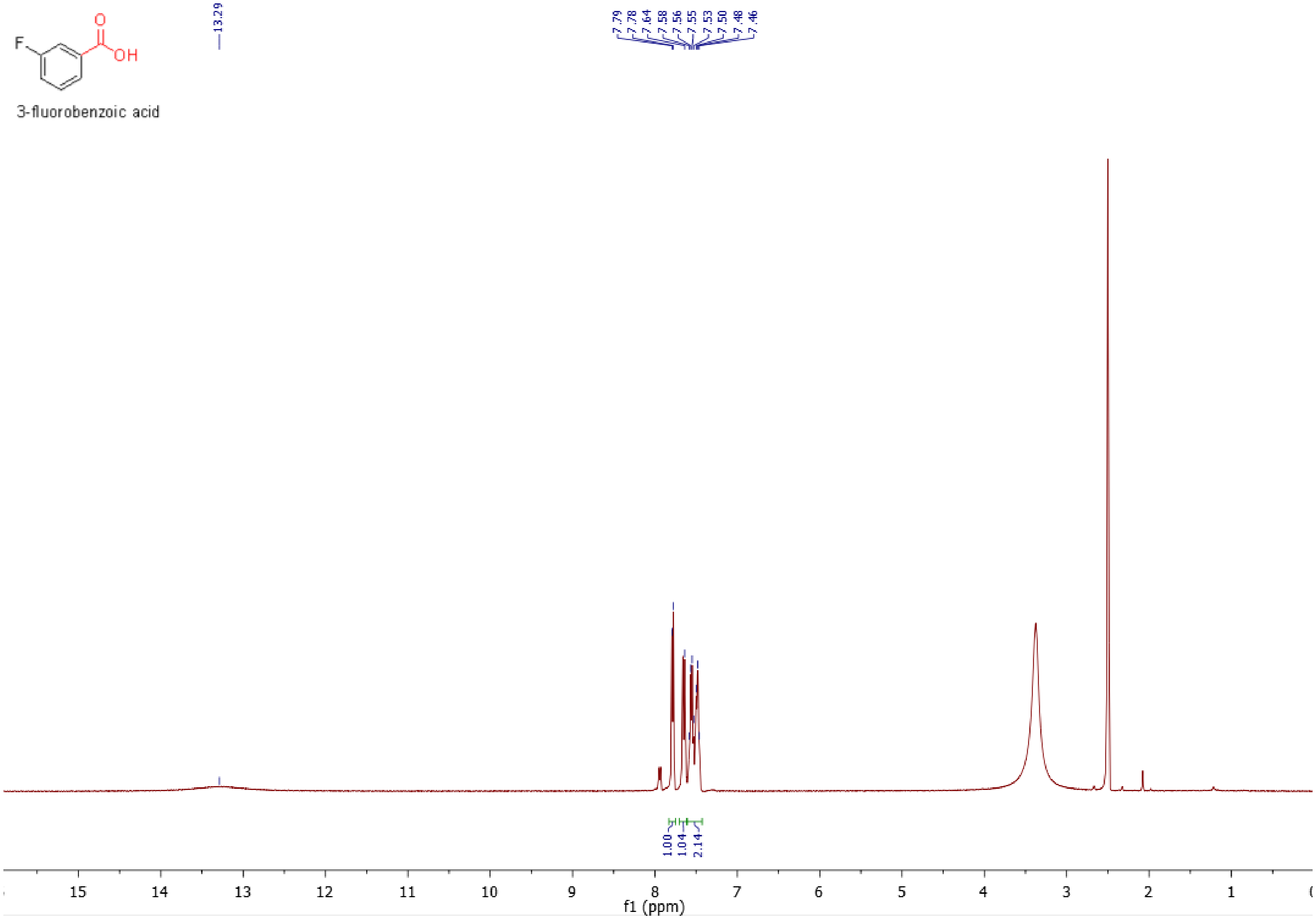
F
O
OH
3-fluorobenzoic acid
13.29
7.79
7.78
7.64
7.58
7.56
7.55
7.53
7.50
7.48
7.46
1.00
1.04
2.14
15
14
13
12
11
10
9
8
7
6
5
4
3
2
1
f1 (ppm)

^{13}C NMR

2c

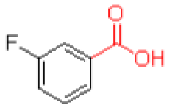
F
O
OH
3-fluorobenzoic acid

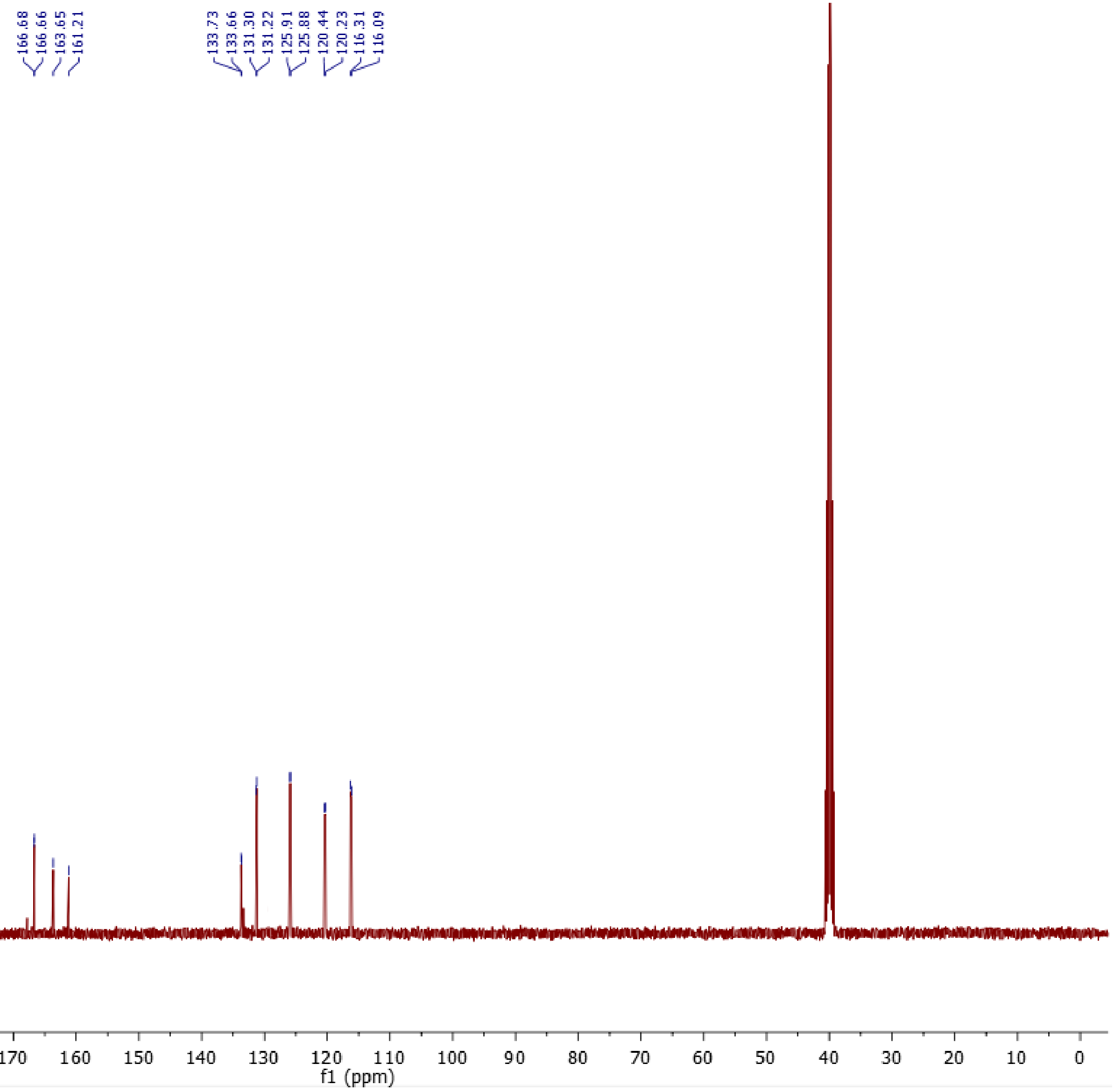
166.68
166.66
163.65
161.21
133.73
133.66
131.30
131.22
125.91
125.88
120.44
120.23
116.31
116.09
230 220 210 200 190 180 170 160 150 140 130 120 110 100 90 80 70 60 50 40 30 20 10 0
f1 (ppm)

^{19}F NMR

2c

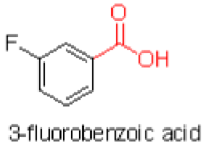
F
O
OH
3-fluorobenzoic acid

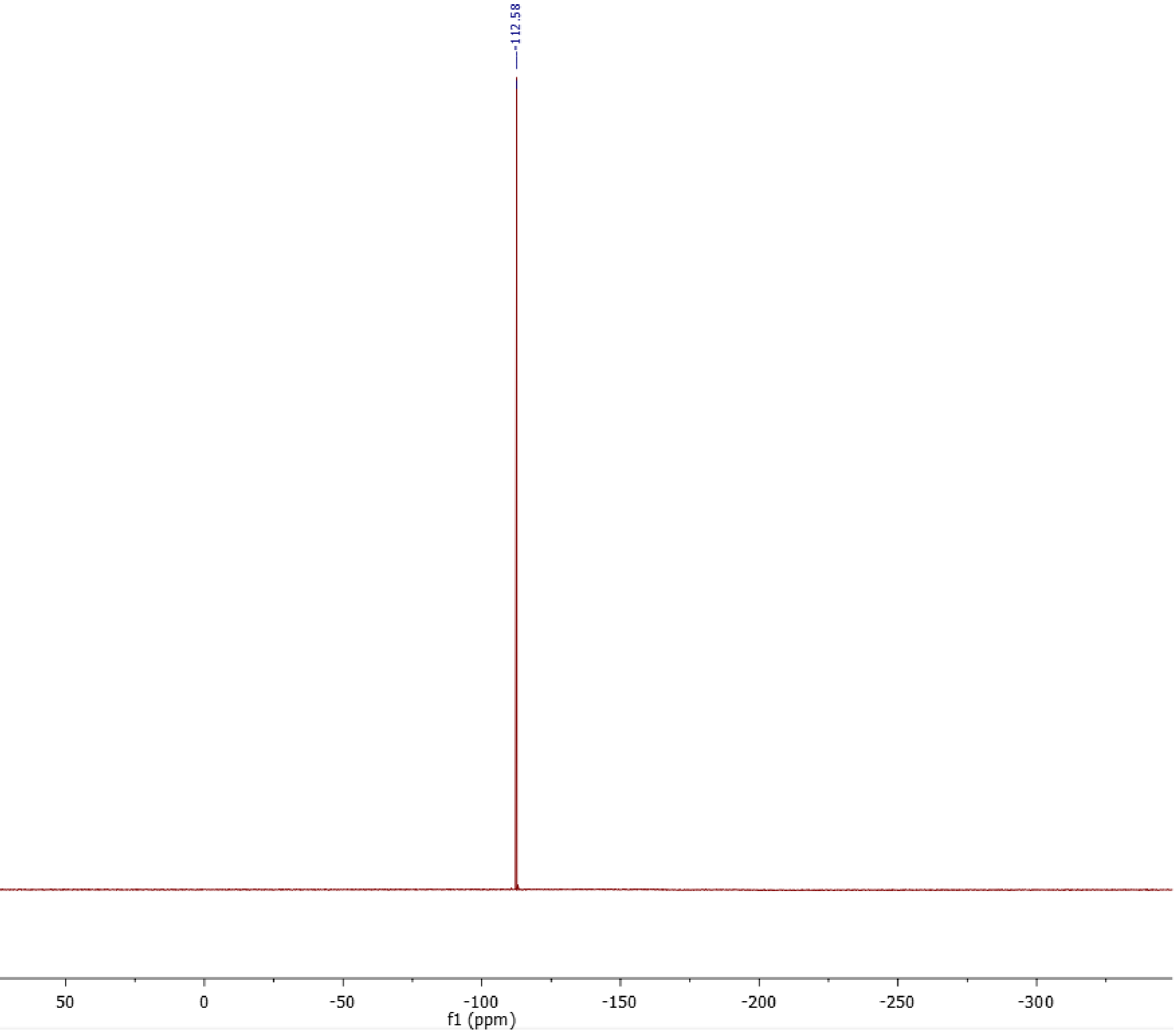
-112.58
100
50
0
-50
-100
-150
-200
-250
-300
f1 (ppm)

^{1}H NMR

2d

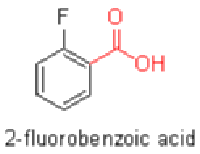
F
O
OH
2-fluorobenzoic acid

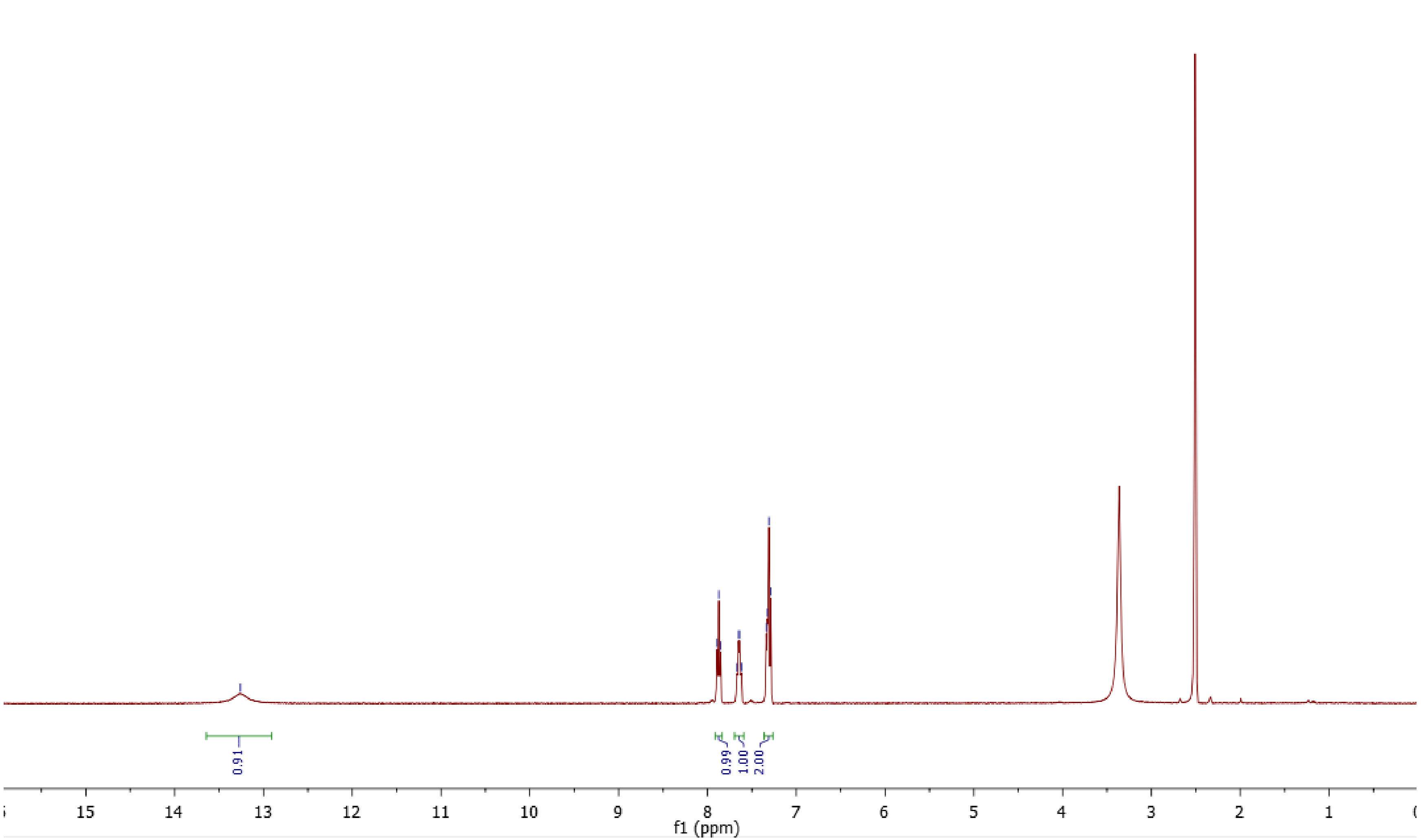
13.26
7.89
7.87
7.85
7.67
7.65
7.64
7.62
7.34
7.33
7.31
7.29
0.91
0.99
1.00
2.00
15
14
13
12
11
10
9
8
7
6
5
4
3
2
1
f1 (ppm)

^{13}C NMR

2d

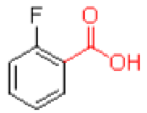

2-fluorobenzoic acid

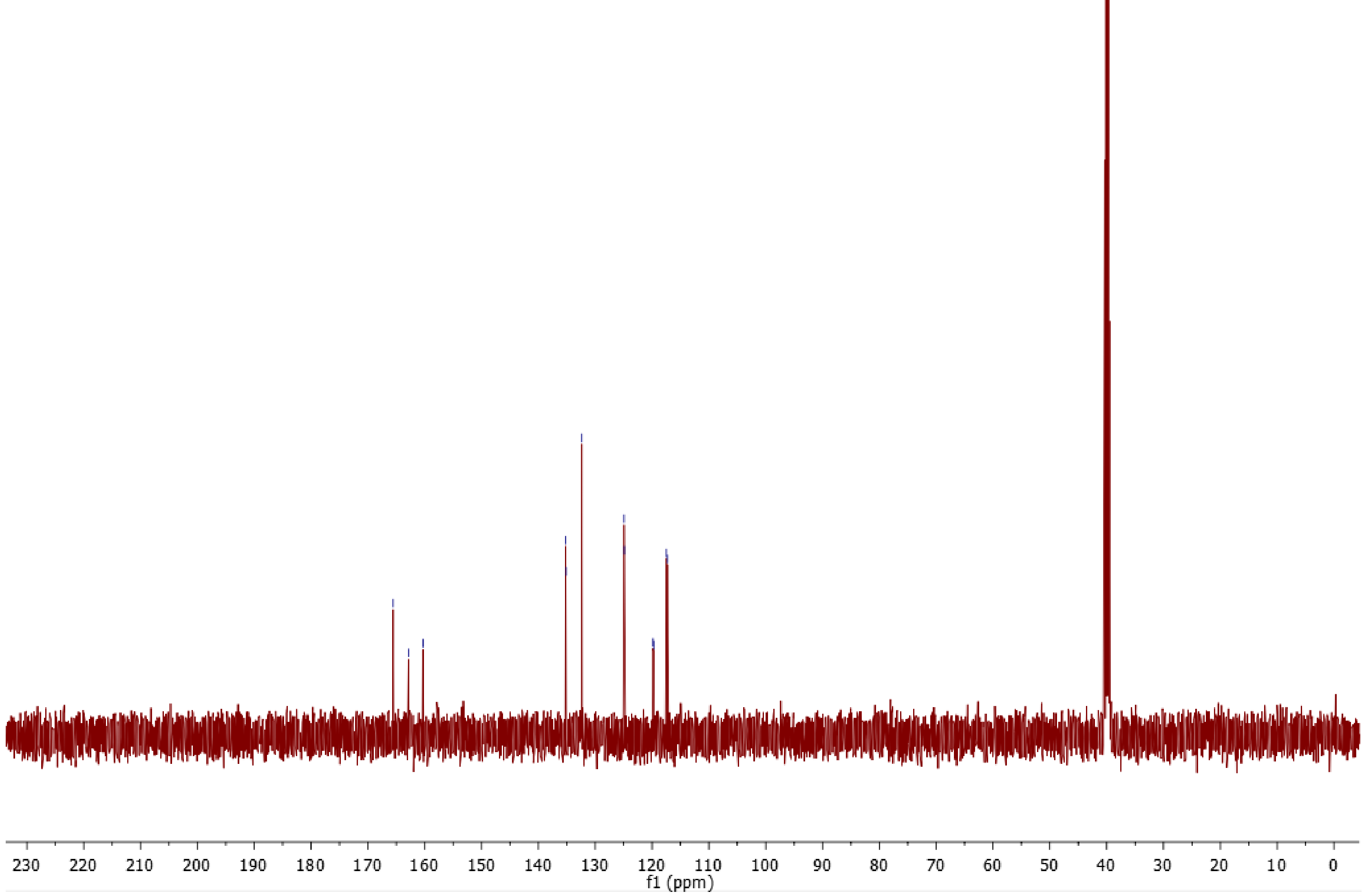

^{19}F NMR

2d

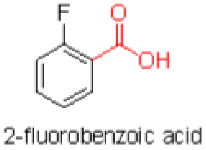
F
O
OH
2-fluorobenzoic acid

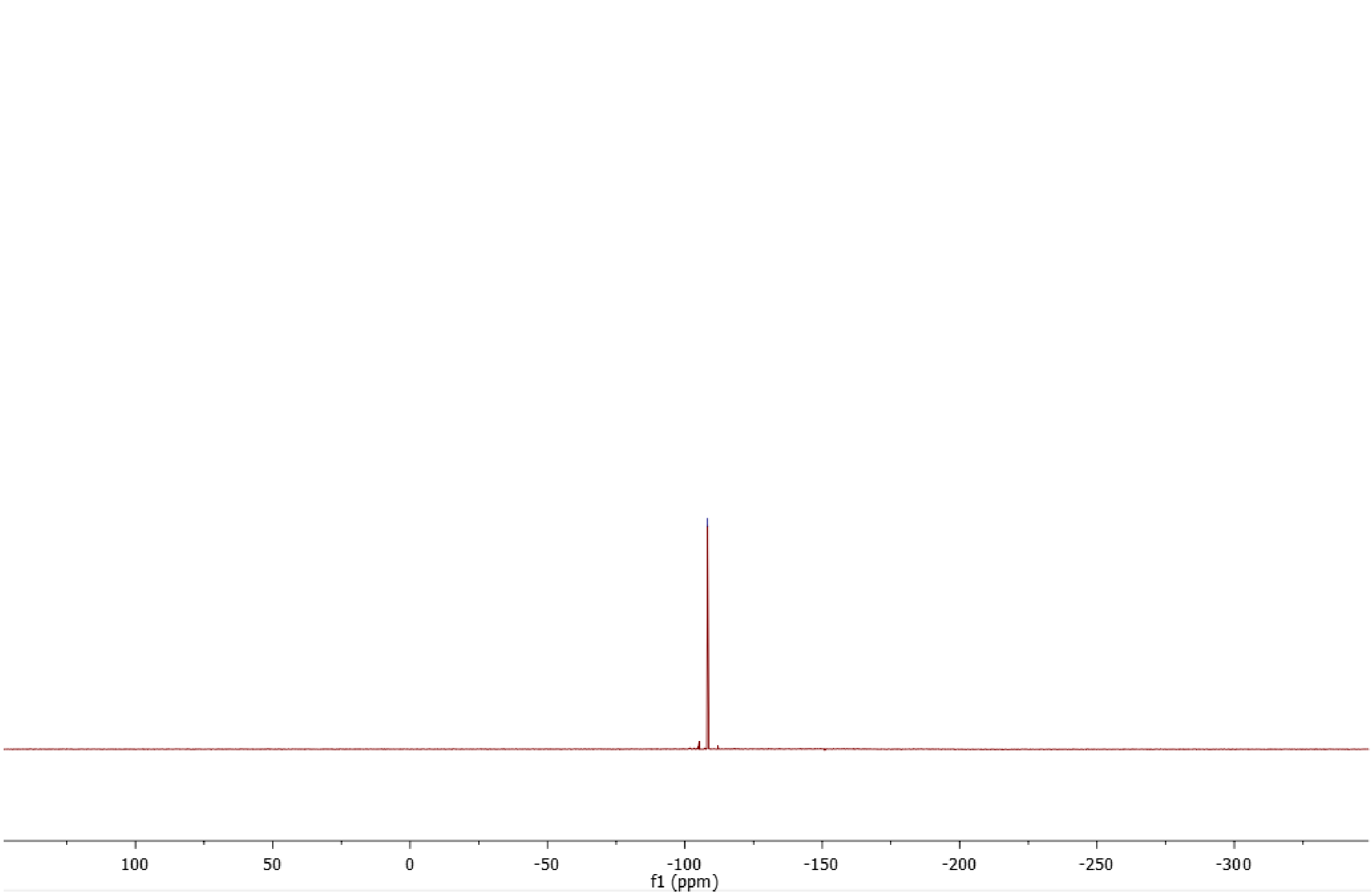
-108.25
100
50
0
-50
-100
-150
-200
-250
-300
f1 (ppm)

^{1}H NMR

2e

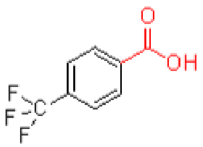

4-(trifluoromethyl)benzoic acid

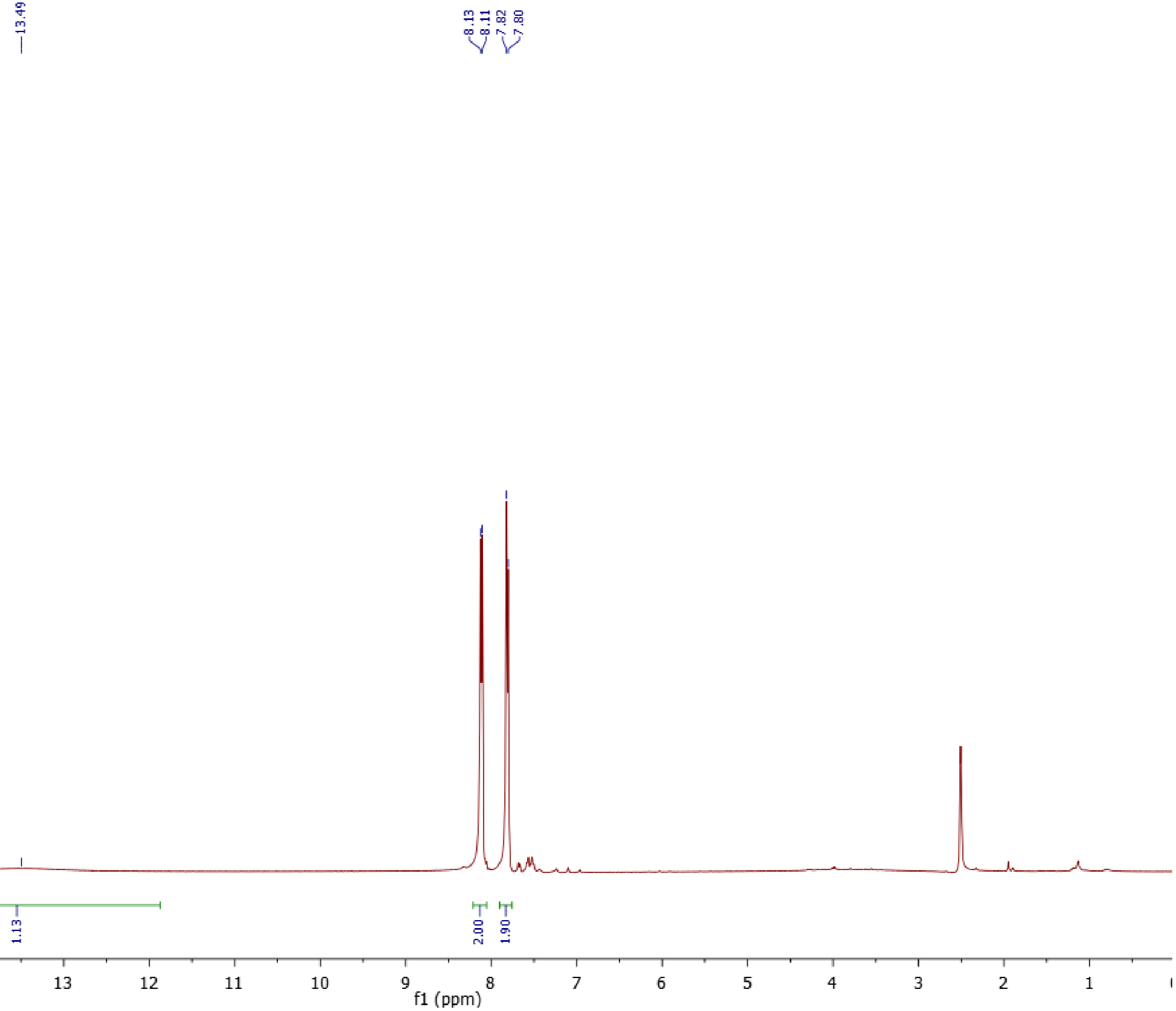

^{13}C NMR

2e

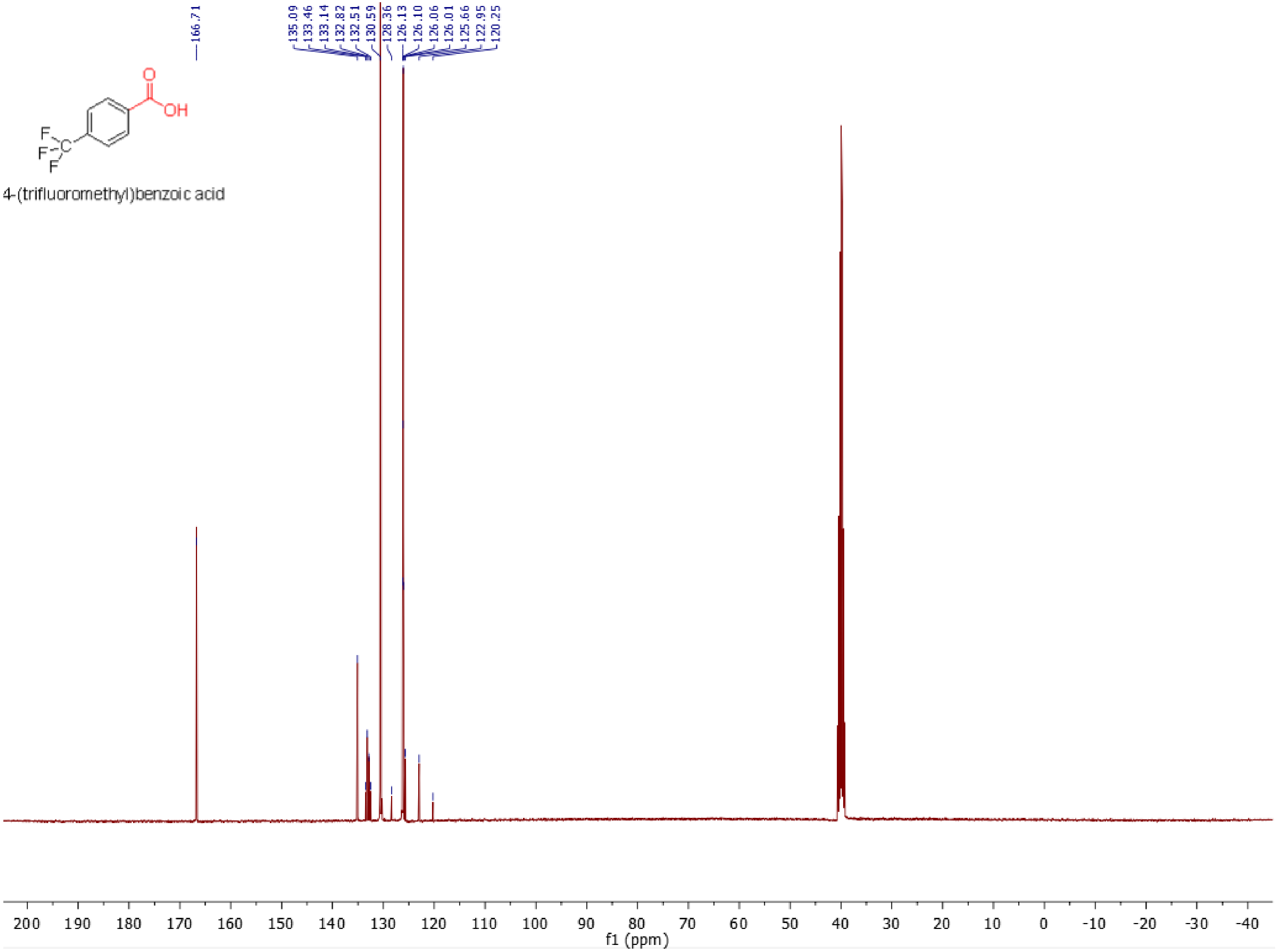
166.71
135.09
133.46
133.14
132.82
132.51
130.59
128.36
126.13
126.10
126.06
126.01
125.66
122.95
120.25
O
OH
F
F
C
F
4-(trifluoromethyl)benzoic acid
200 190 180 170 160 150 140 130 120 110 100 90 80 70 60 50 40 30 20 10 0 -10 -20 -30 -40
f1 (ppm)

^{19}F NMR

2e

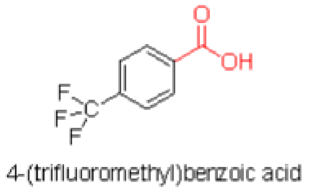
O
OH
F
F
C
F
4-(trifluoromethyl)benzoic acid

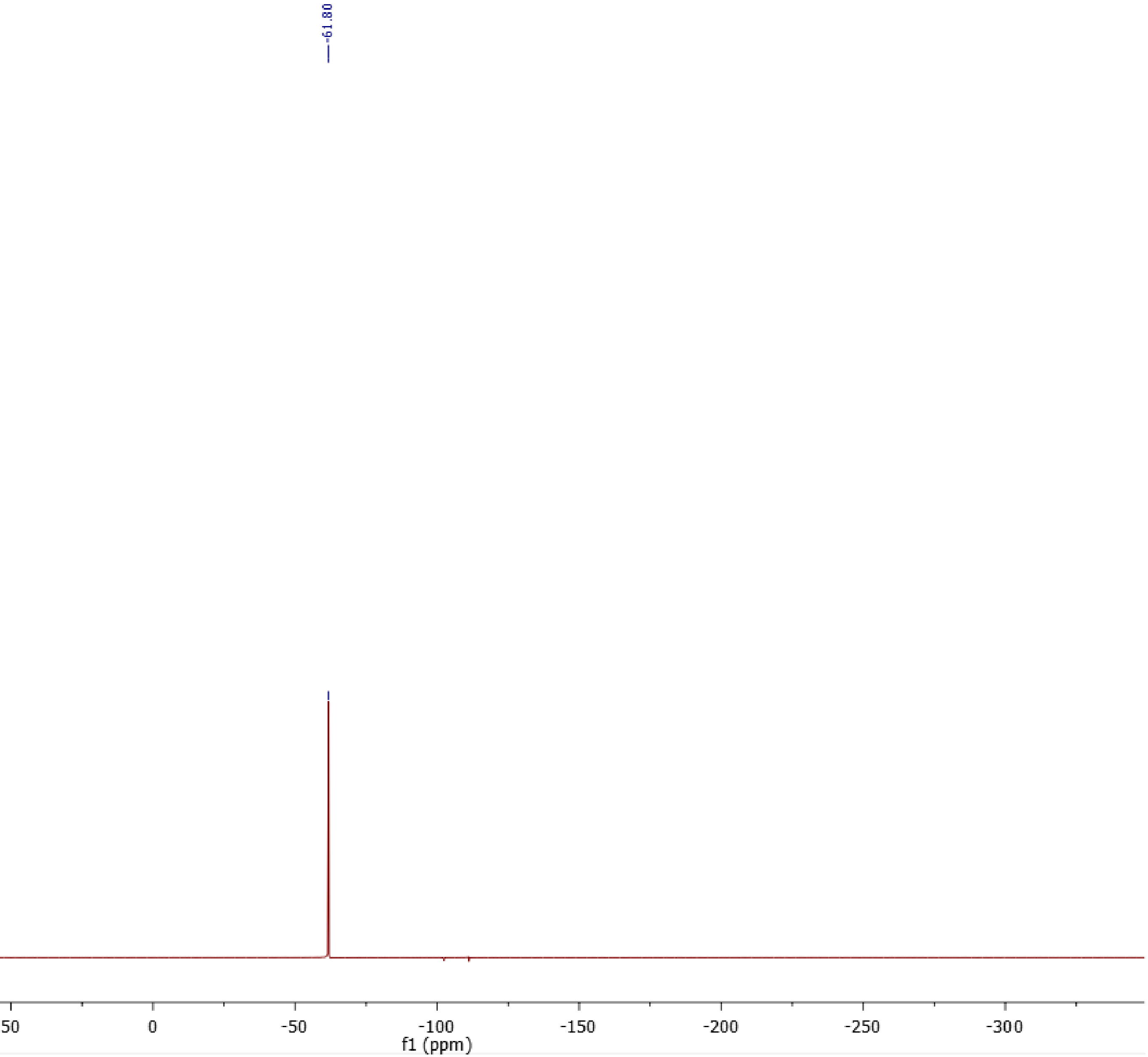
-61.80
100
50
0
-50
-100
-150
-200
-250
-300
f1 (ppm)

^{1}H NMR

2f

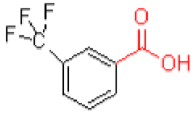
OH
3-(trifluoromethyl)benzoic acid

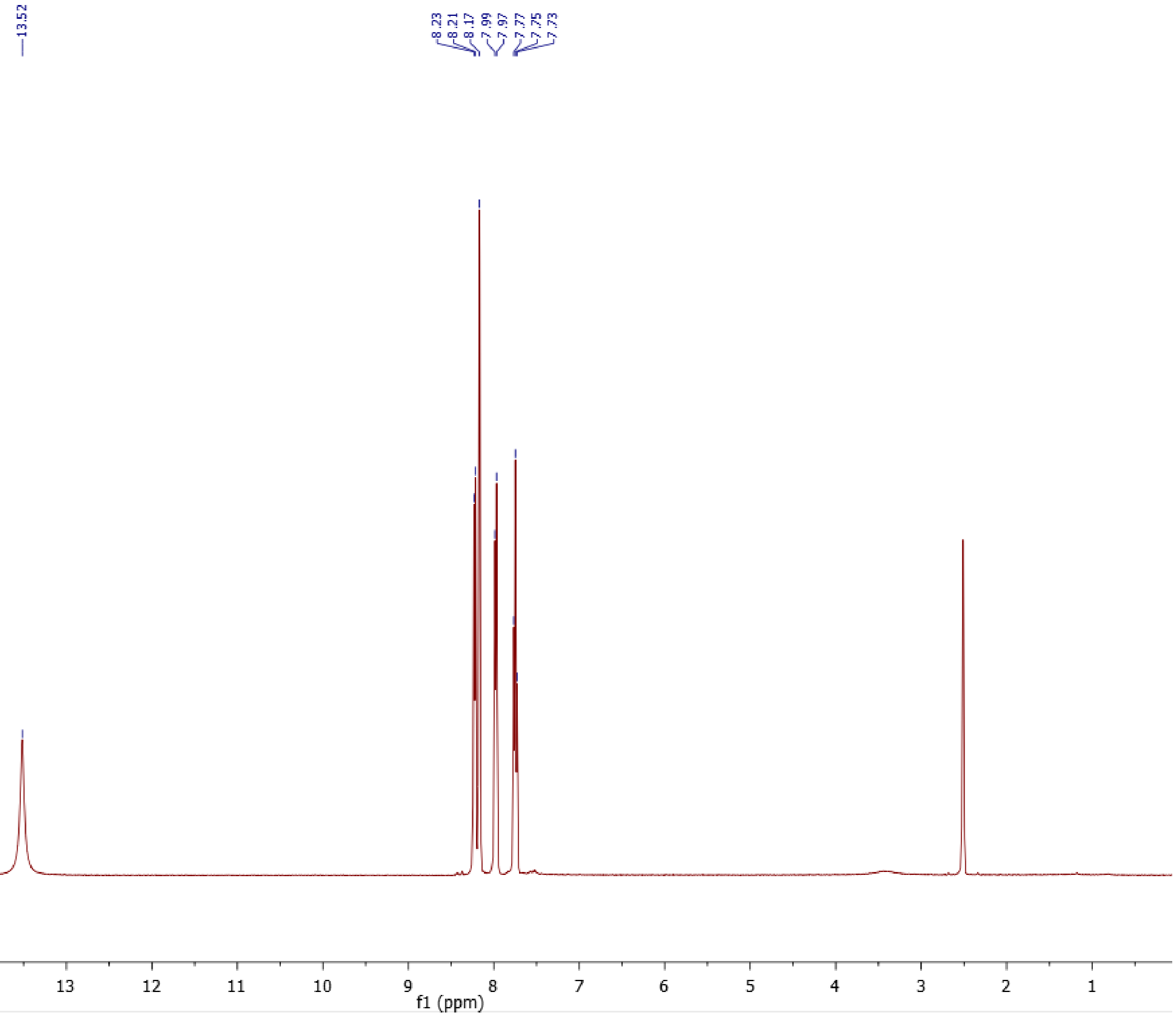
13.52
8.23
8.21
8.17
7.99
7.97
7.77
7.75
7.73
16
15
14
13
12
11
10
9
8
7
6
5
4
3
2
1
f1 (ppm)

^{13}C NMR

2f

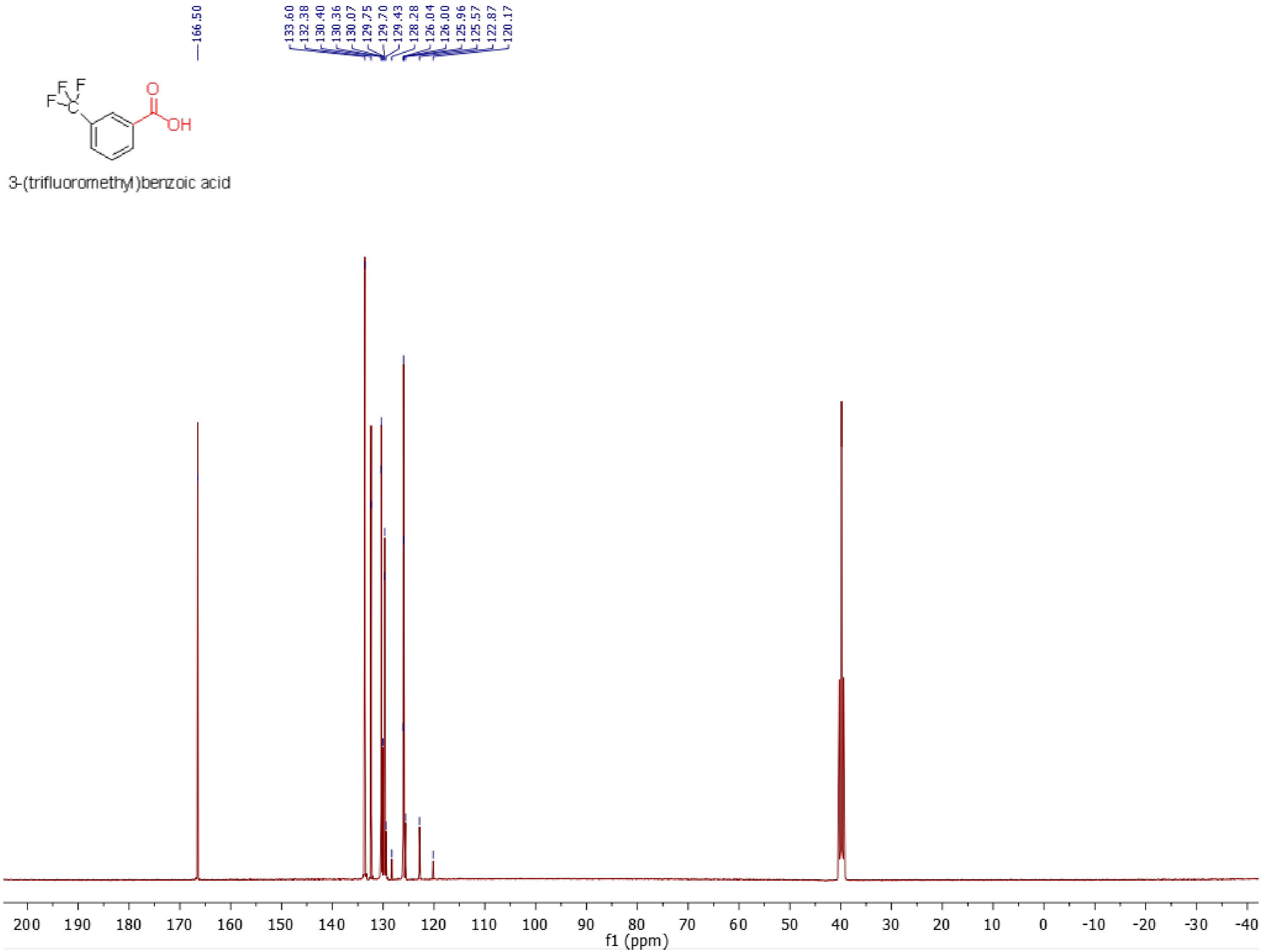

166.50
133.60
132.38
130.40
130.36
130.07
129.75
129.70
129.43
128.28
126.04
126.00
125.96
125.57
122.87
120.17
3-(trifluoromethyl)benzoic acid
200 190 180 170 160 150 140 130 120 110 100 90 80 70 60 50 40 30 20 10 0 -10 -20 -30 -40
f1 (ppm)

^{19}F NMR

2f

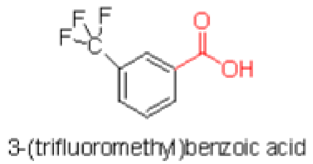

3-(trifluoromethyl)benzoic acid

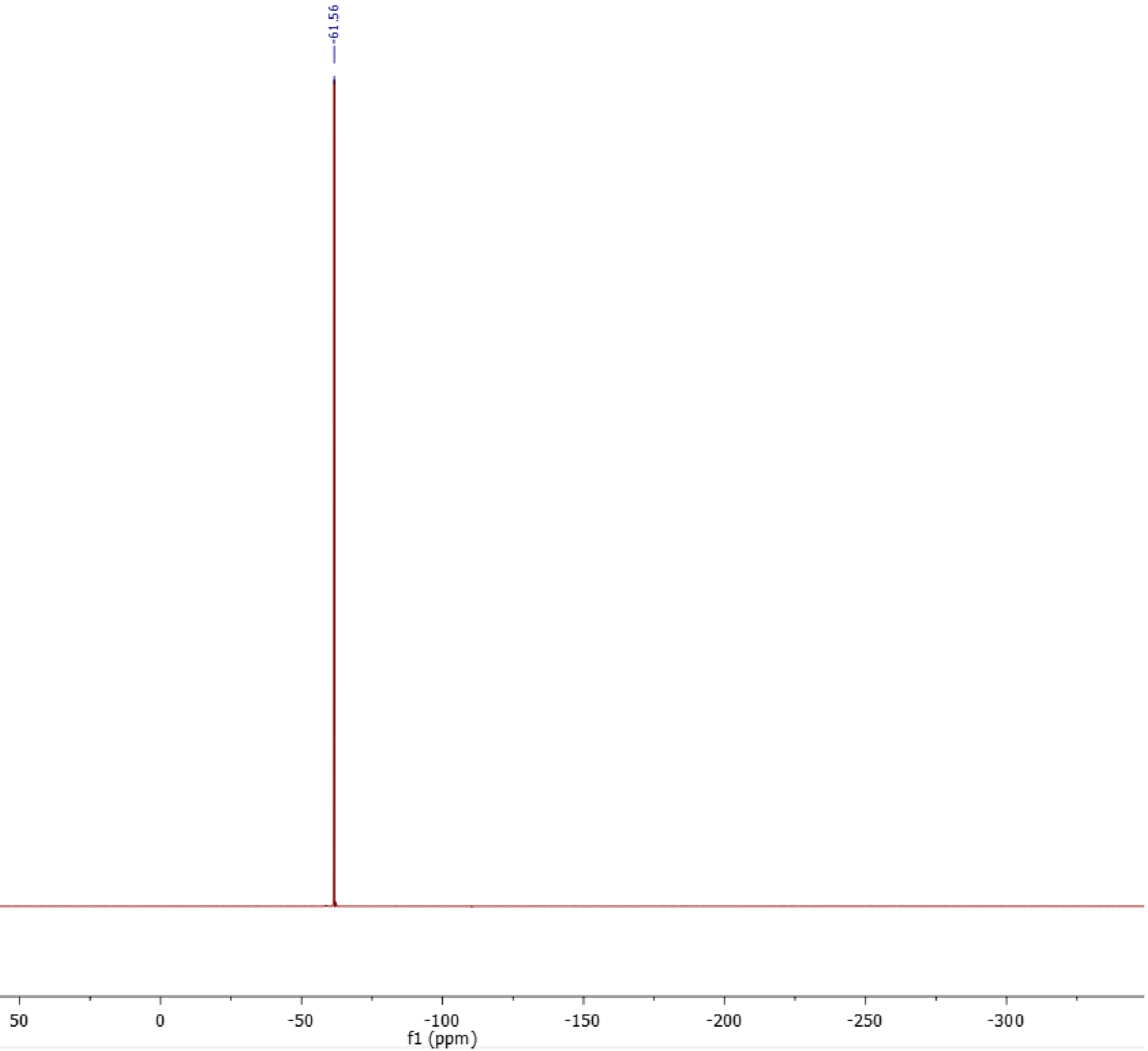

-61.56
100
50
0
-50
-100
-150
-200
-250
-300
f1 (ppm)

^{1}H NMR

2g

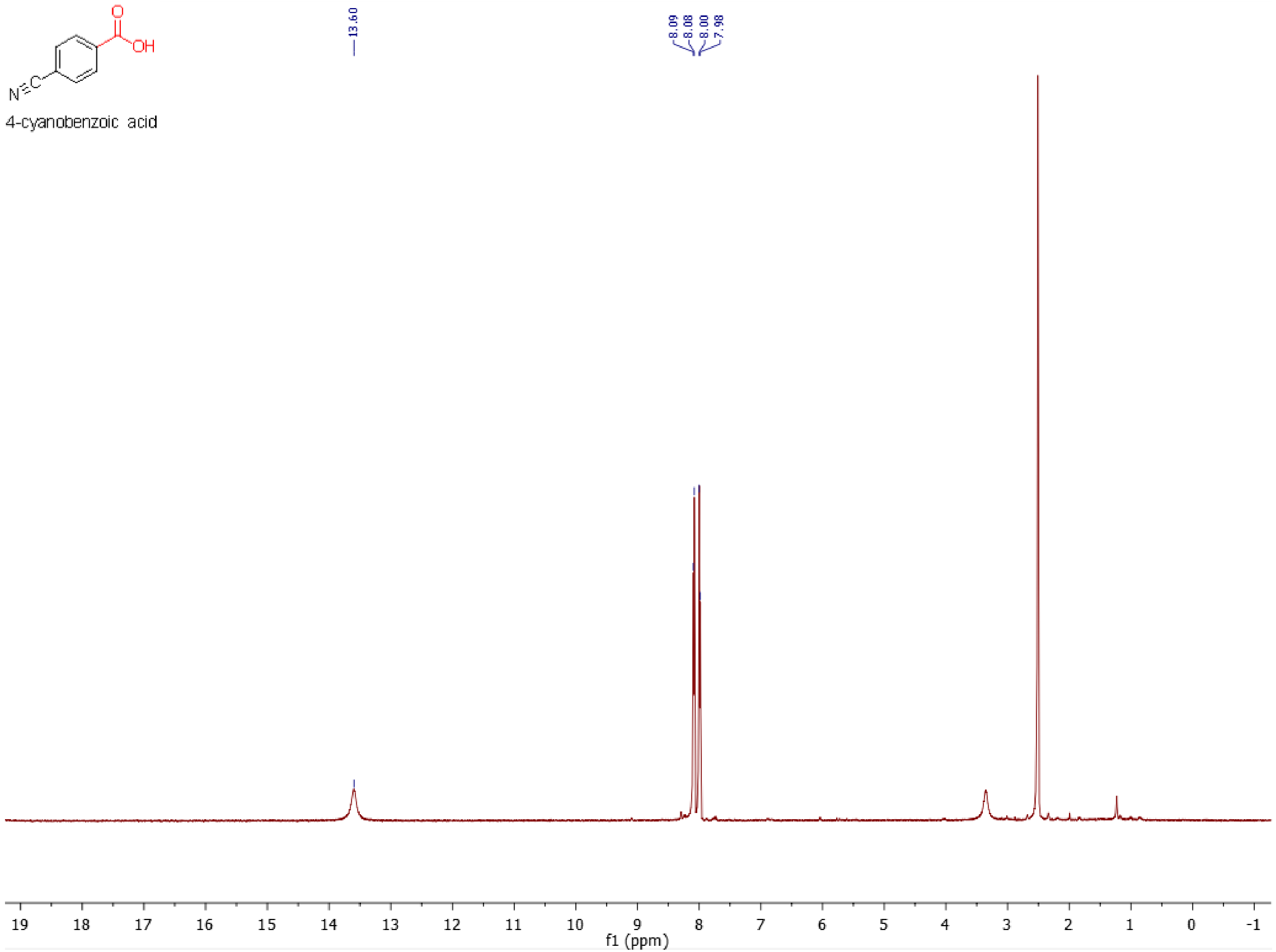

4-cyanobenzoic acid
13.60
8.09
8.08
8.00
7.98
19 18 17 16 15 14 13 12 11 10 9 8 7 6 5 4 3 2 1 0 -1
f1 (ppm)

^{13}C NMR

2g

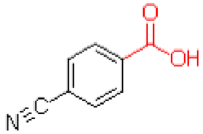

4-cyanobenzoic acid

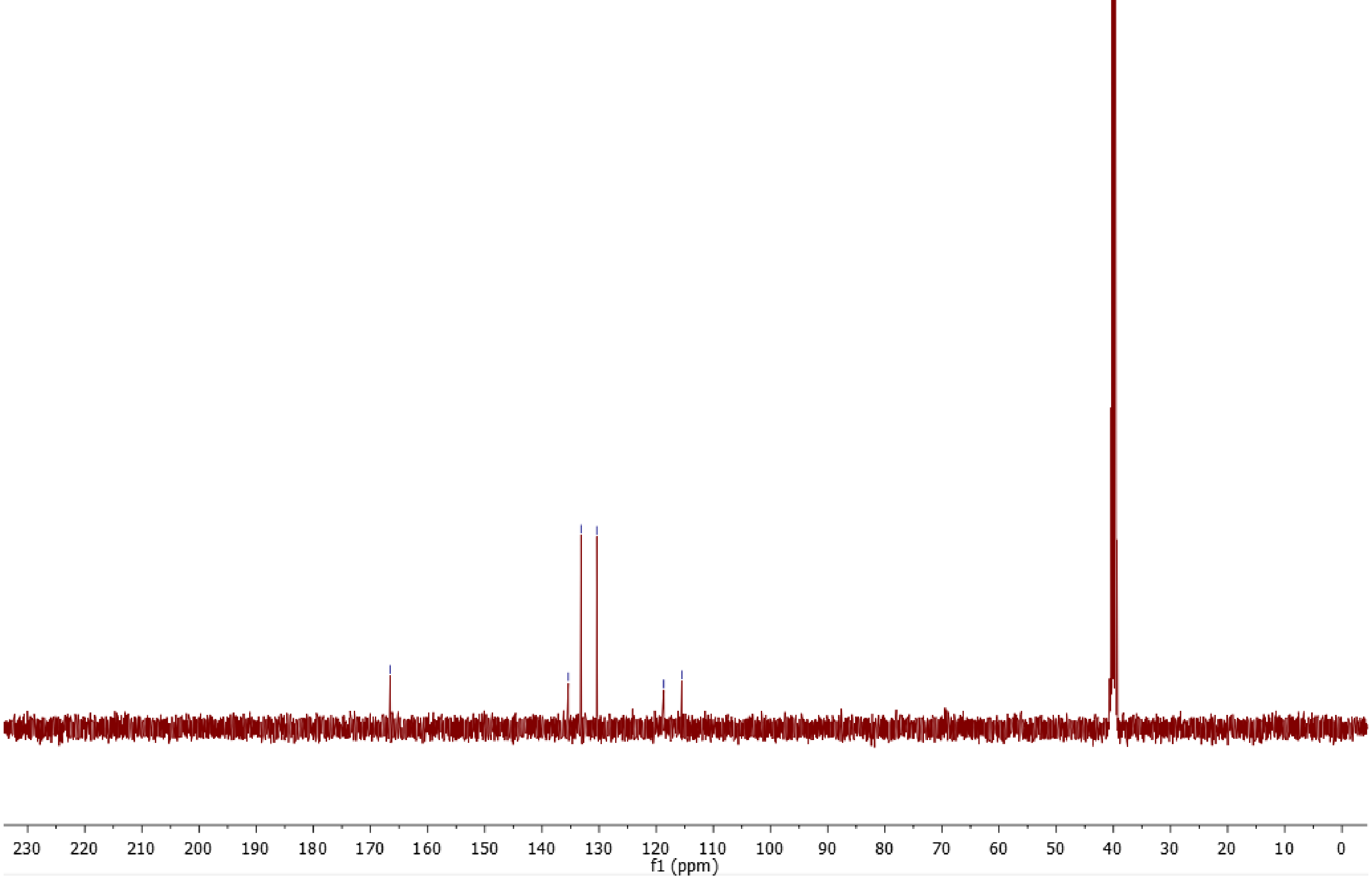

^{1}H NMR

2h

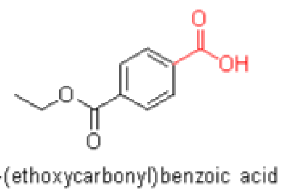

O
OH
O
O
4-(ethoxycarbonyl)benzoic acid

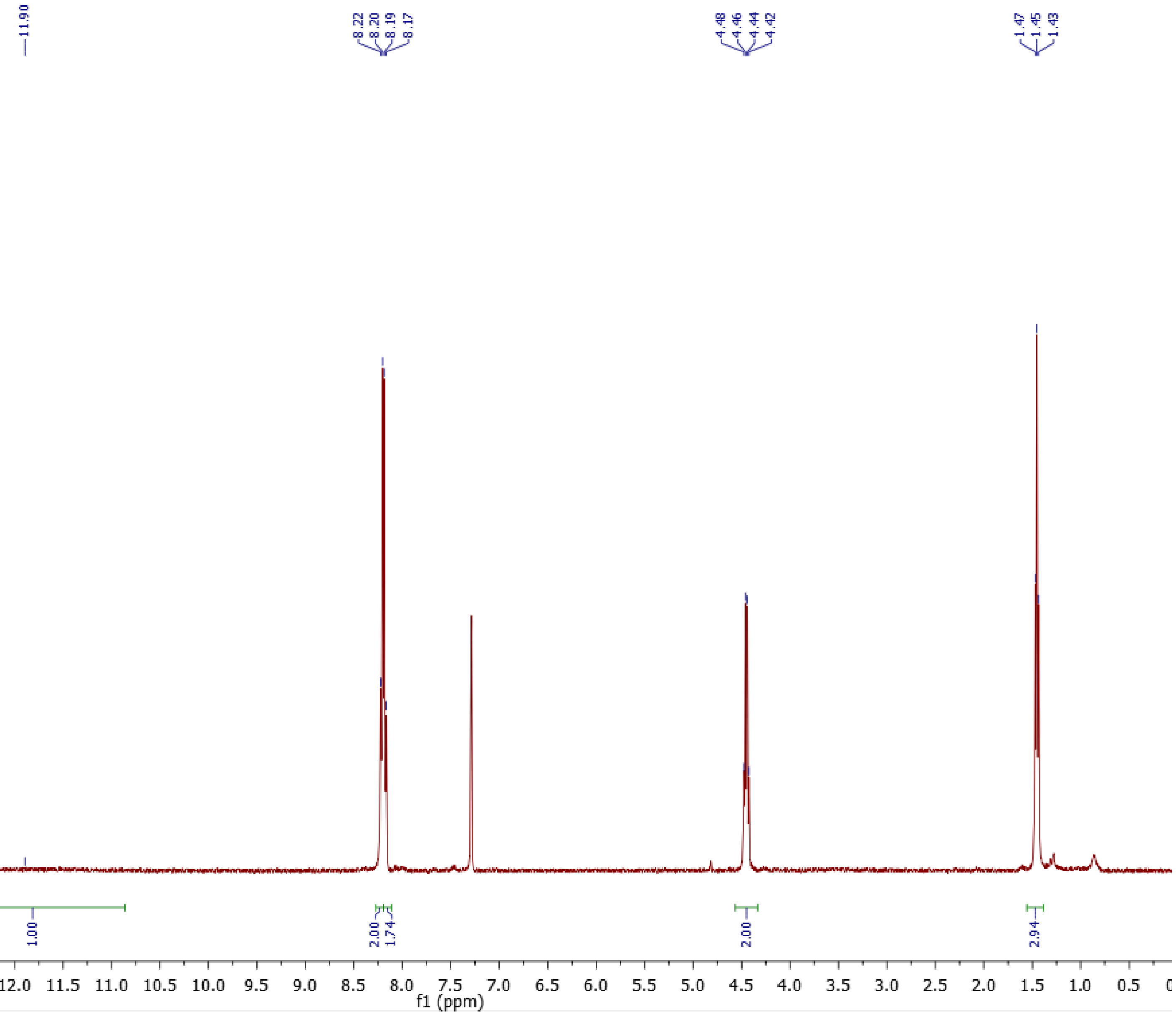

11.90
8.22
8.20
8.19
8.17
4.48
4.46
4.44
4.42
1.47
1.45
1.43
1.00
2.00
1.74
2.00
2.94
0 14.5 14.0 13.5 13.0 12.5 12.0 11.5 11.0 10.5 10.0 9.5 9.0 8.5 8.0 7.5 7.0 6.5 6.0 5.5 5.0 4.5 4.0 3.5 3.0 2.5 2.0 1.5 1.0 0.5 0
f1 (ppm)

^{13}C NMR

2h

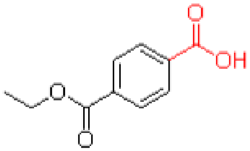

4-(ethoxycarbonyl)benzoic acid

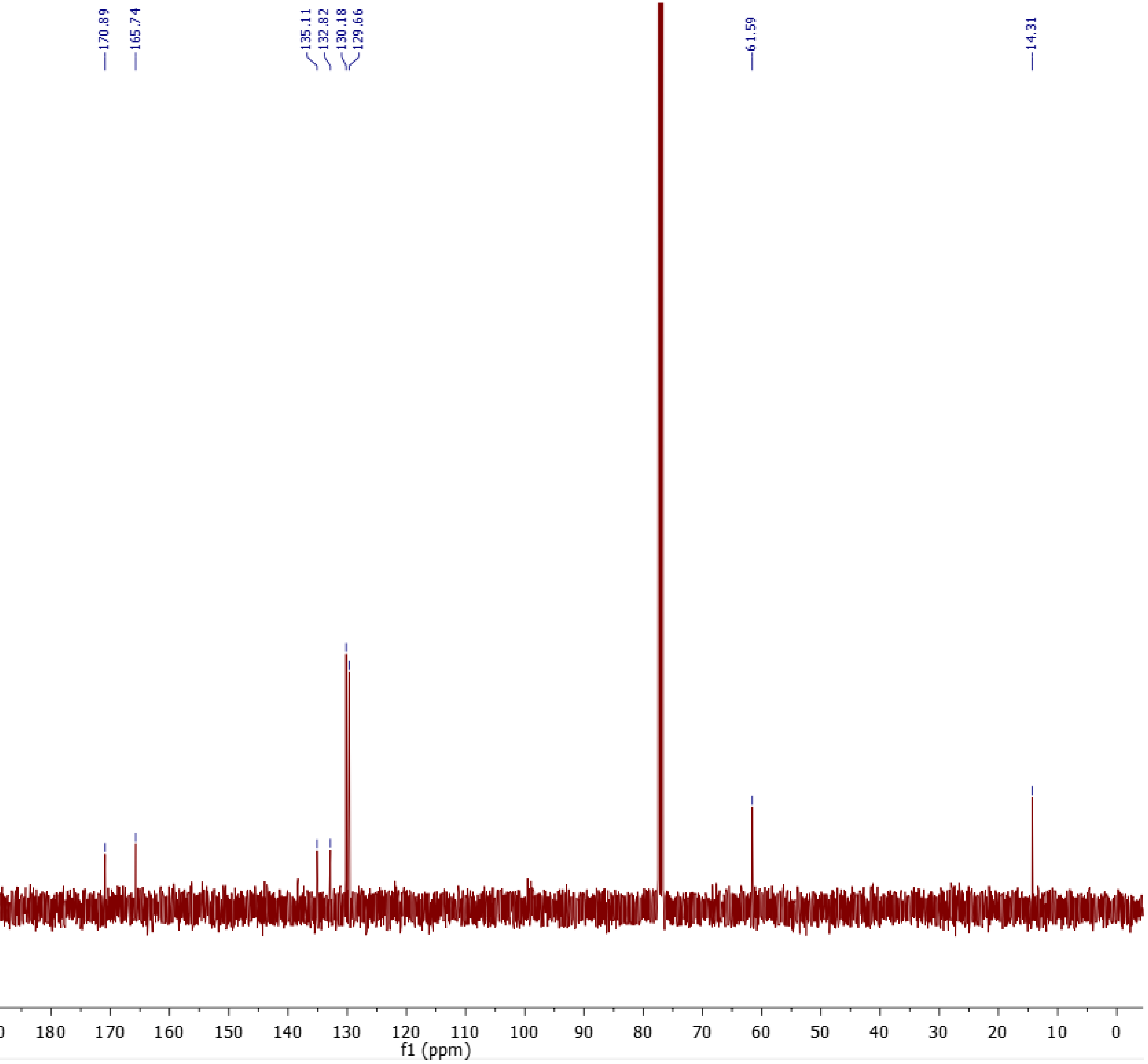

^{1}H NMR

2i

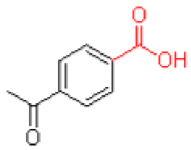

4-acetylbenzoic acid

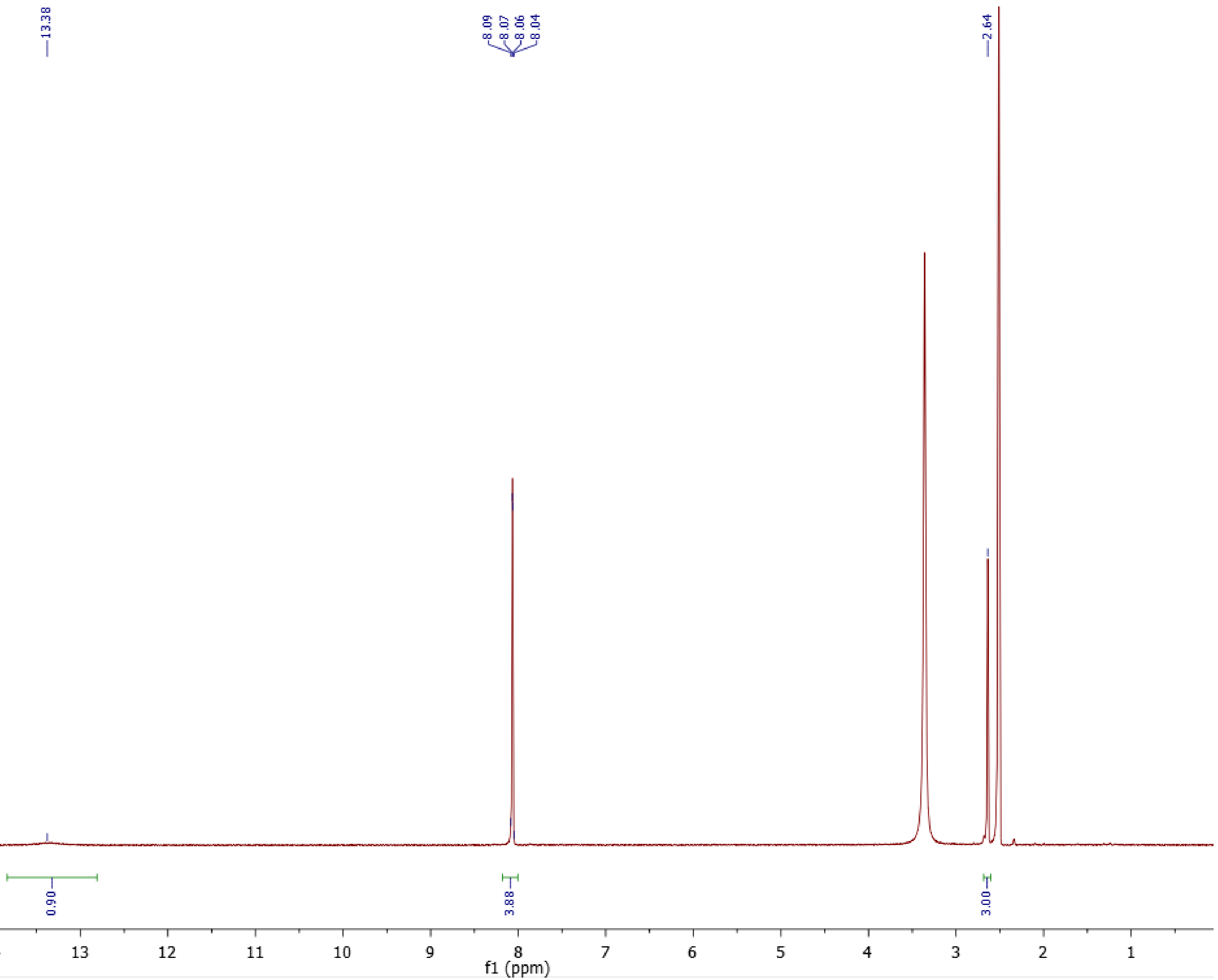

^{13}C NMR

2i

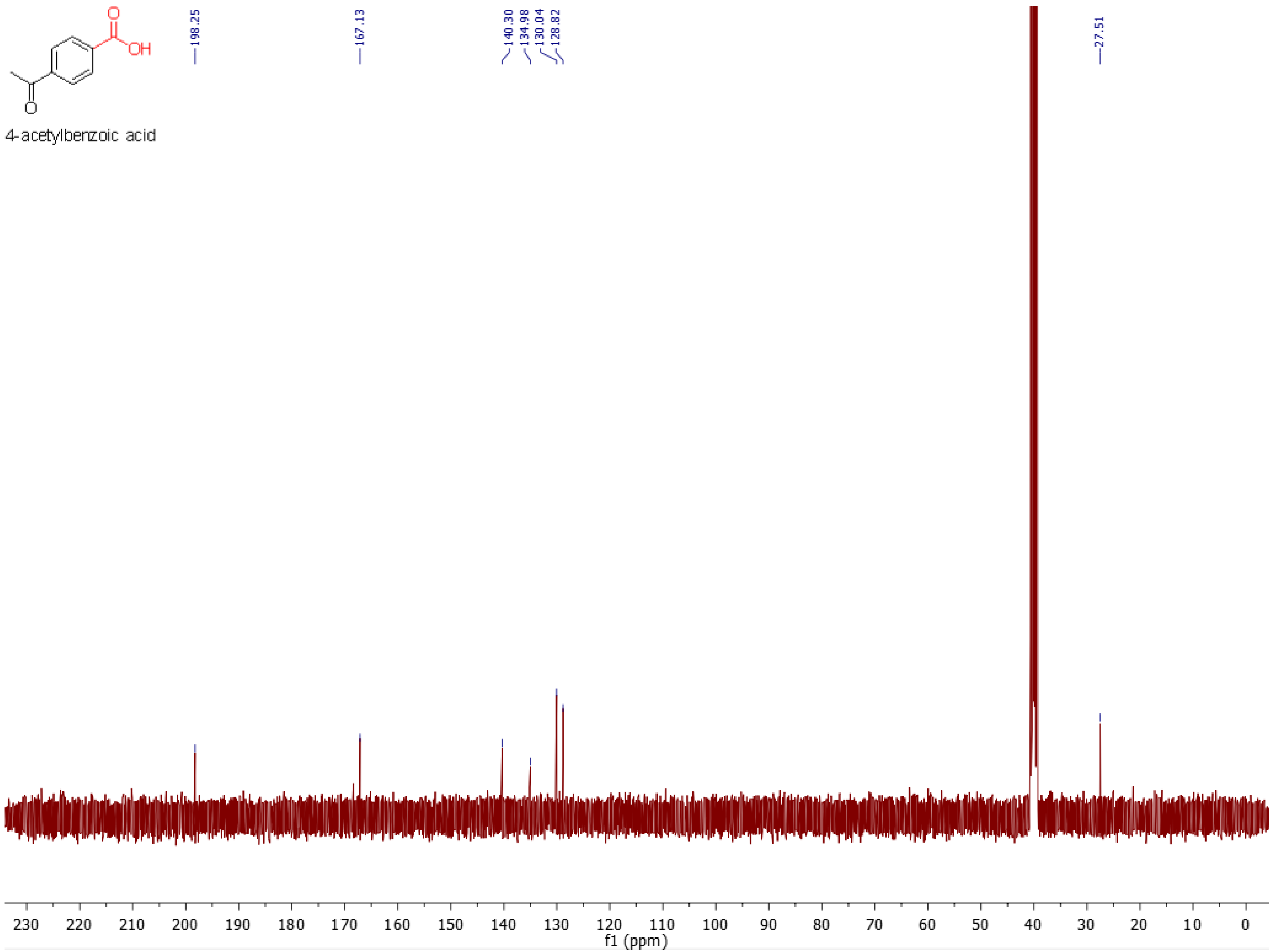

4-acetylbenzoic acid
198.25
167.13
140.30
134.98
130.04
128.82
27.51
230 220 210 200 190 180 170 160 150 140 130 120 110 100 90 80 70 60 50 40 30 20 10 0
f1 (ppm)

^{1}H NMR

2j

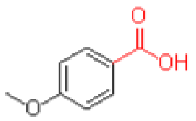

4-methoxybenzoic acid

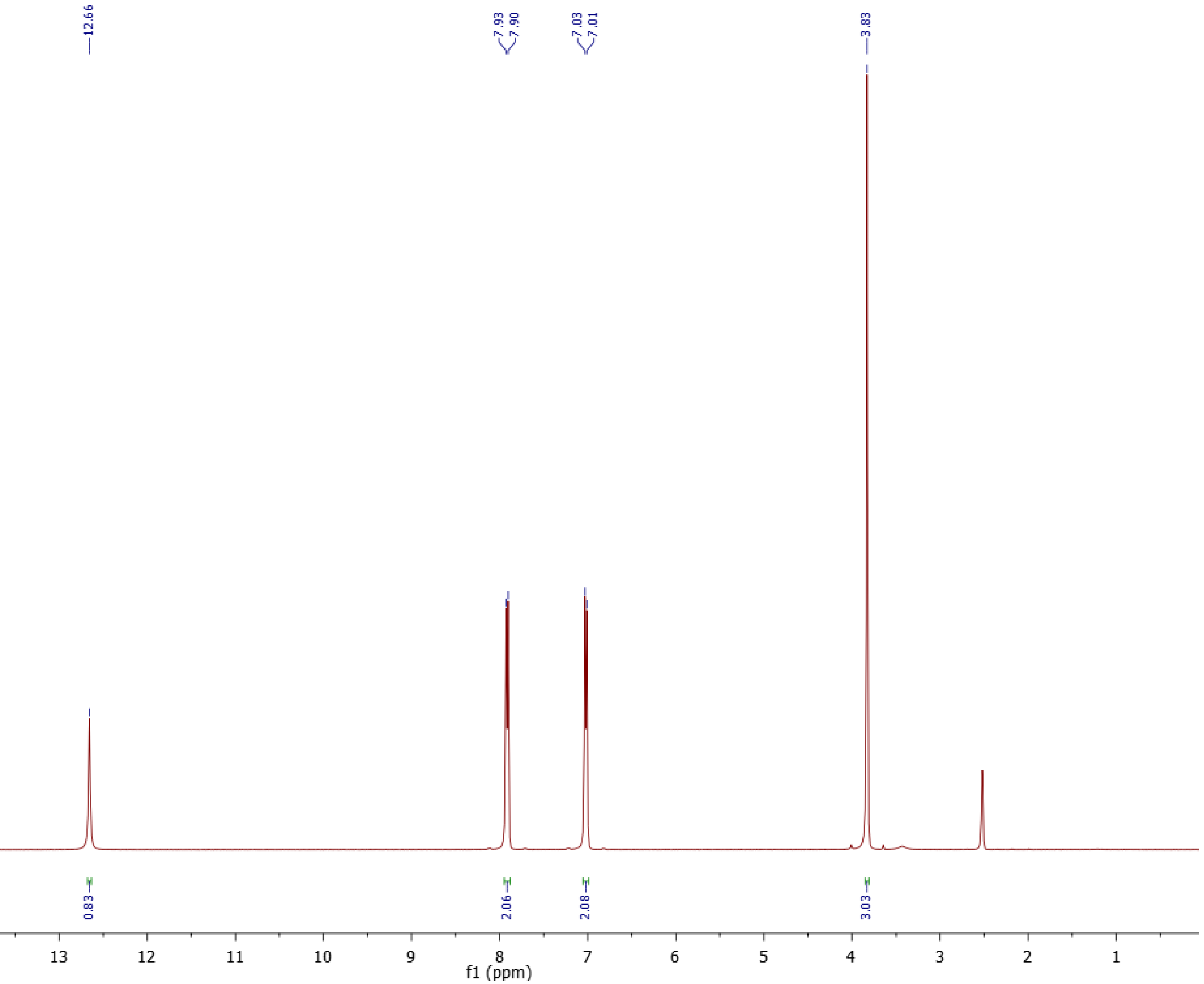

^{13}C NMR

2j

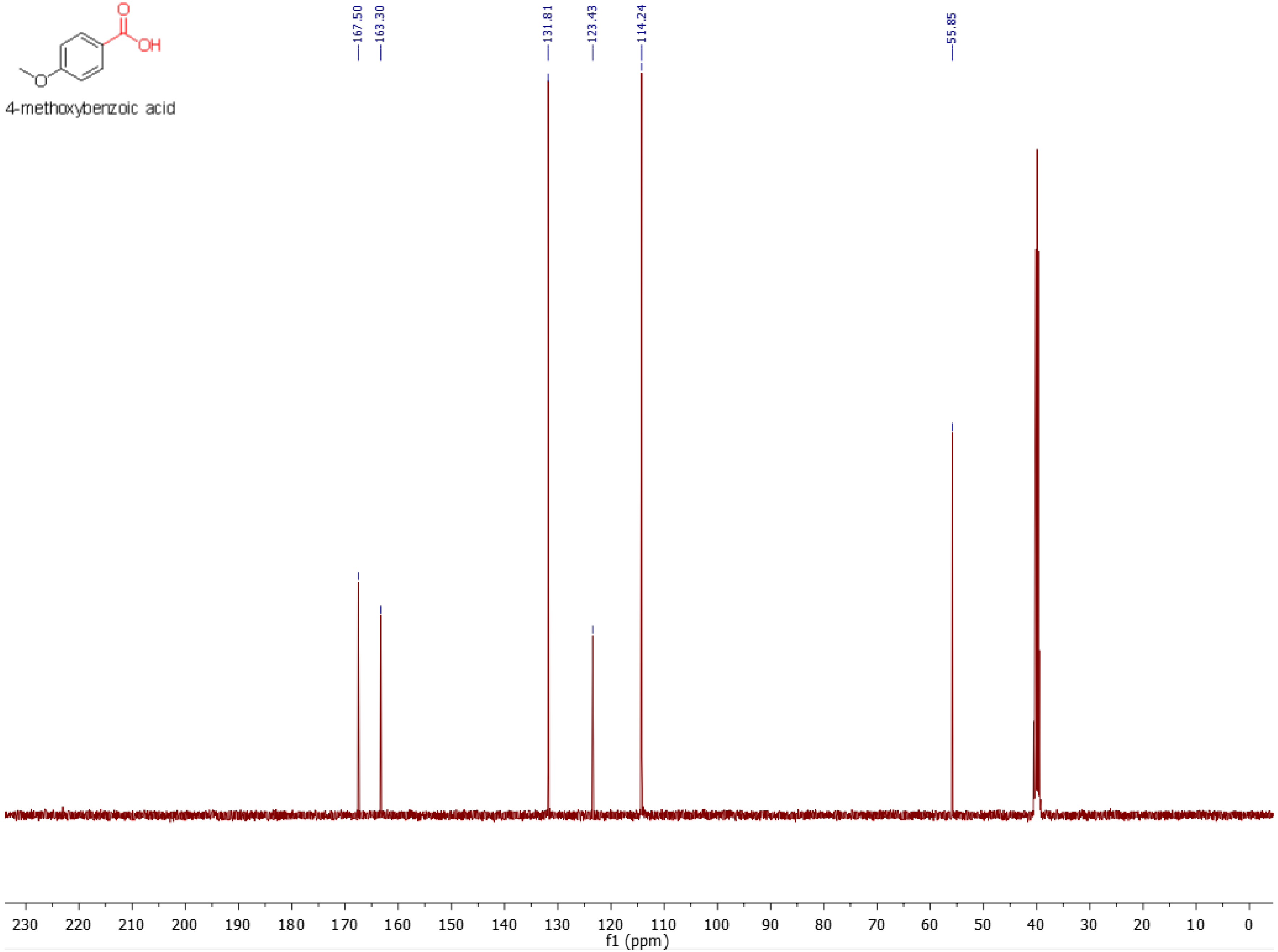

O
OH
O
4-methoxybenzoic acid
167.50
163.30
131.81
123.43
114.24
55.85
230 220 210 200 190 180 170 160 150 140 130 120 110 100 90 80 70 60 50 40 30 20 10 0
f1 (ppm)

^{1}H NMR

2k

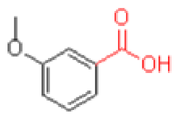

3-methoxybenzoic acid

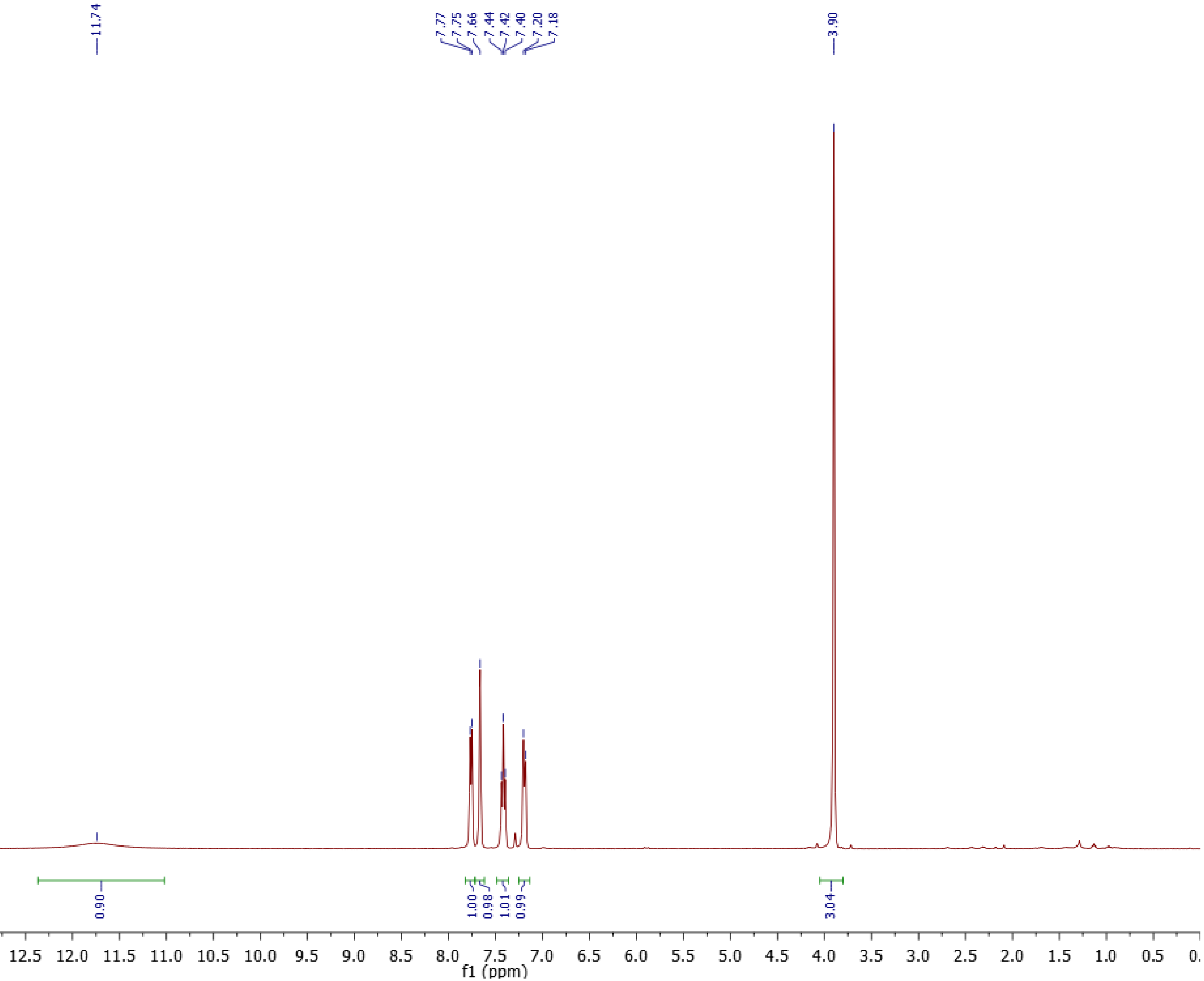

^{13}C NMR

2k

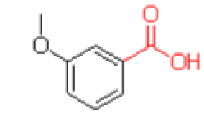

3-methoxybenzoic acid

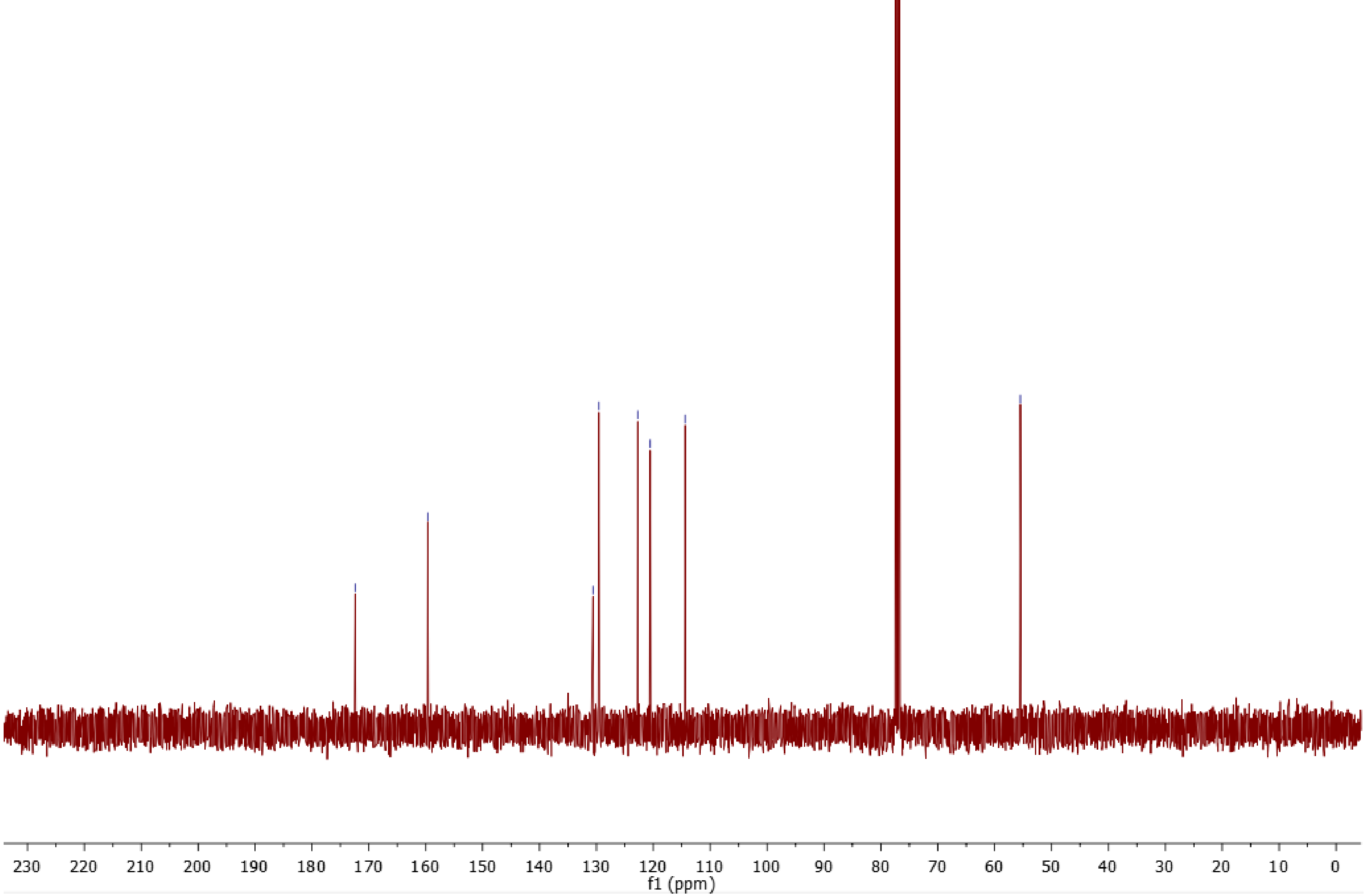

^{1}H NMR

2l

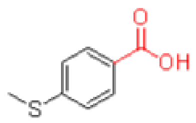
4-(methylthio)benzoic acid

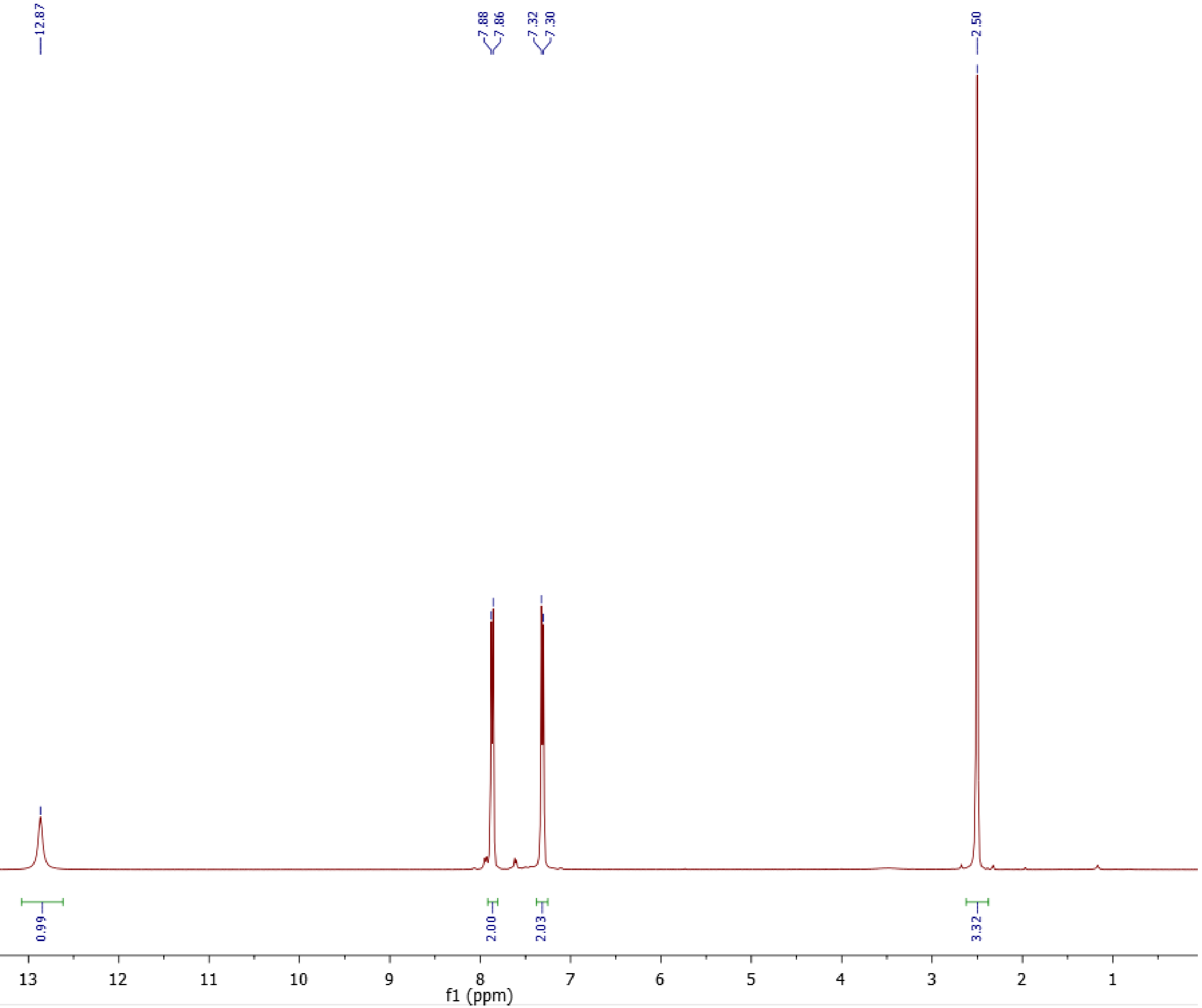

^{13}C NMR

1l

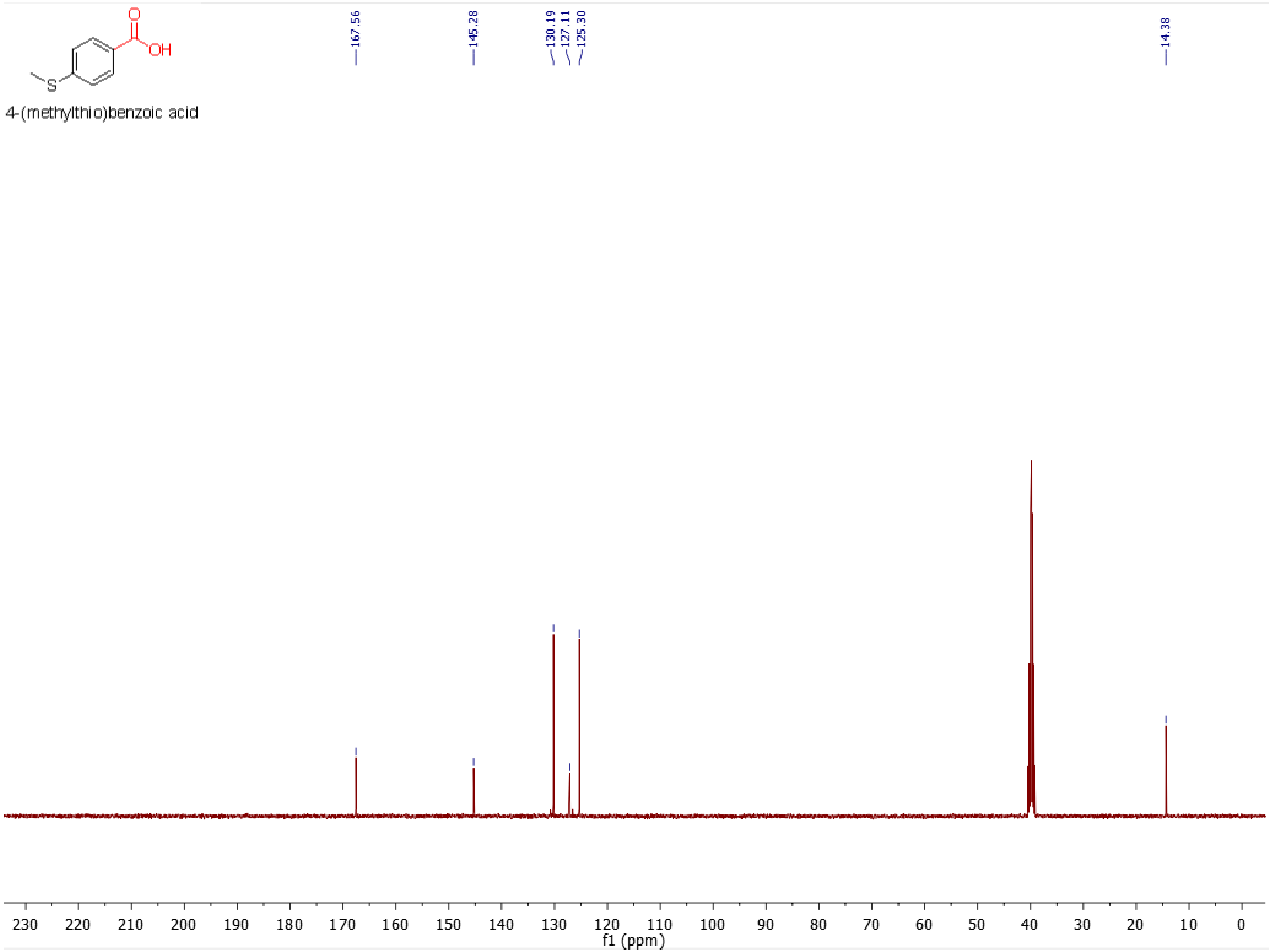

OH
O
S
4-(methylthio)benzoic acid
167.56
145.28
130.19
127.11
125.30
14.38
230 220 210 200 190 180 170 160 150 140 130 120 110 100 90 80 70 60 50 40 30 20 10 0
f1 (ppm)

N CAS 99-94-5

^{1}H NMR

2m

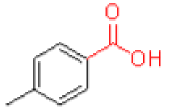

4-methylbenzoic acid

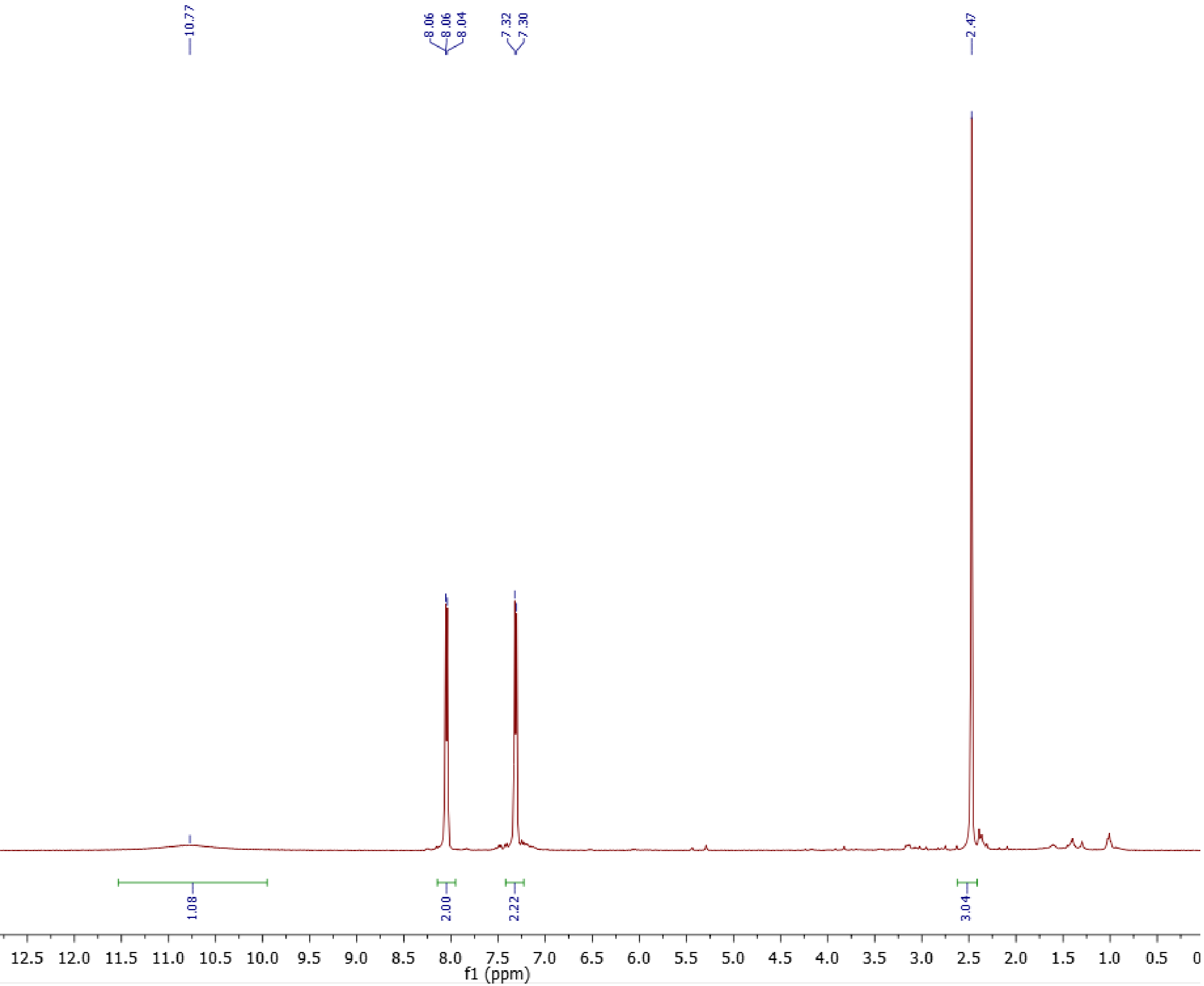

^{13}C NMR

2m

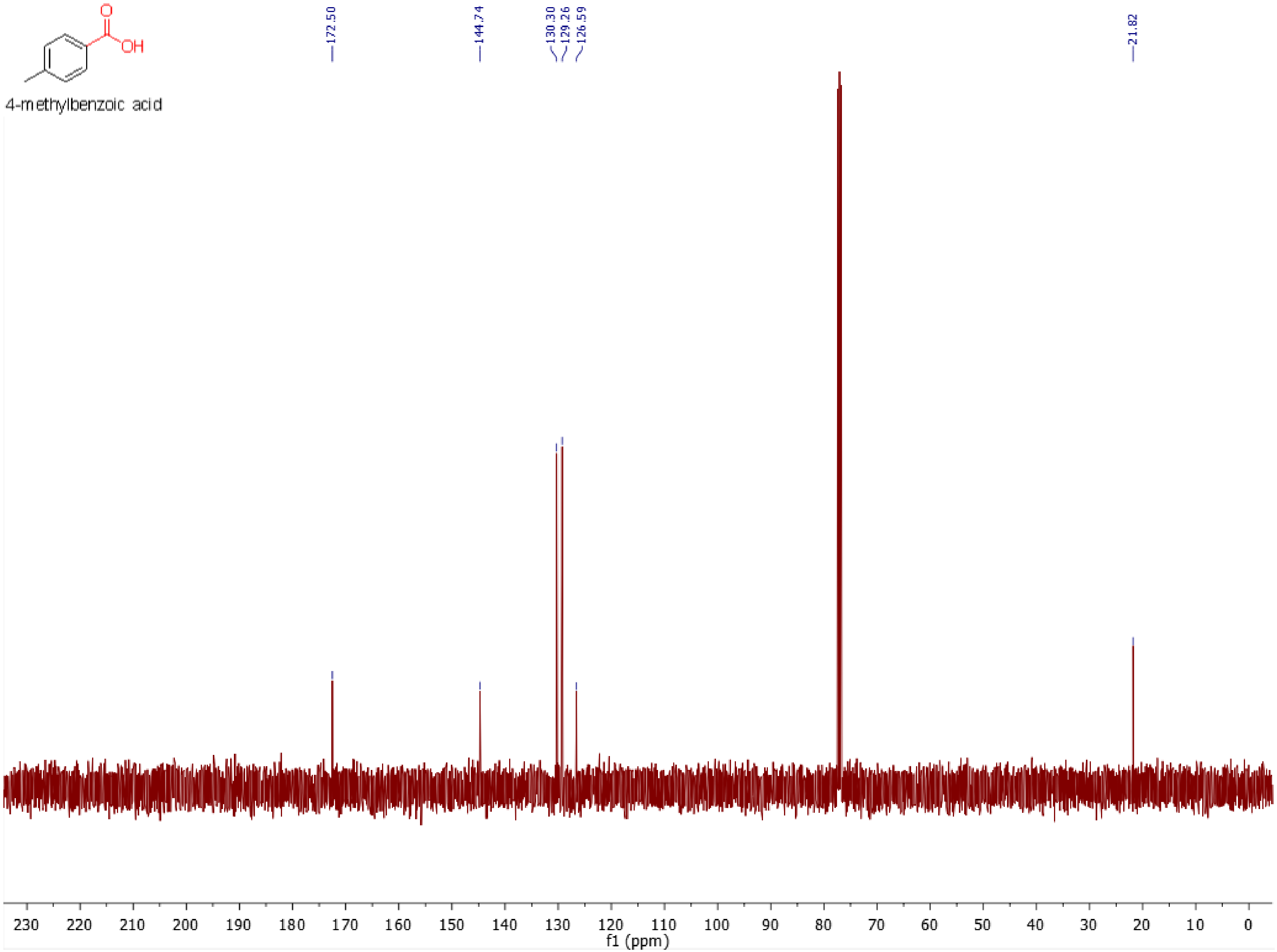

OH
O
4-methylbenzoic acid
172.50
144.74
130.30
129.26
126.59
21.82
230
220
210
200
190
180
170
160
150
140
130
120
110
100
90
80
70
60
50
40
30
20
10
0
f1 (ppm)

^{1}H NMR

2n

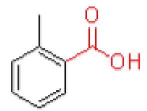

2-methylbenzoic acid

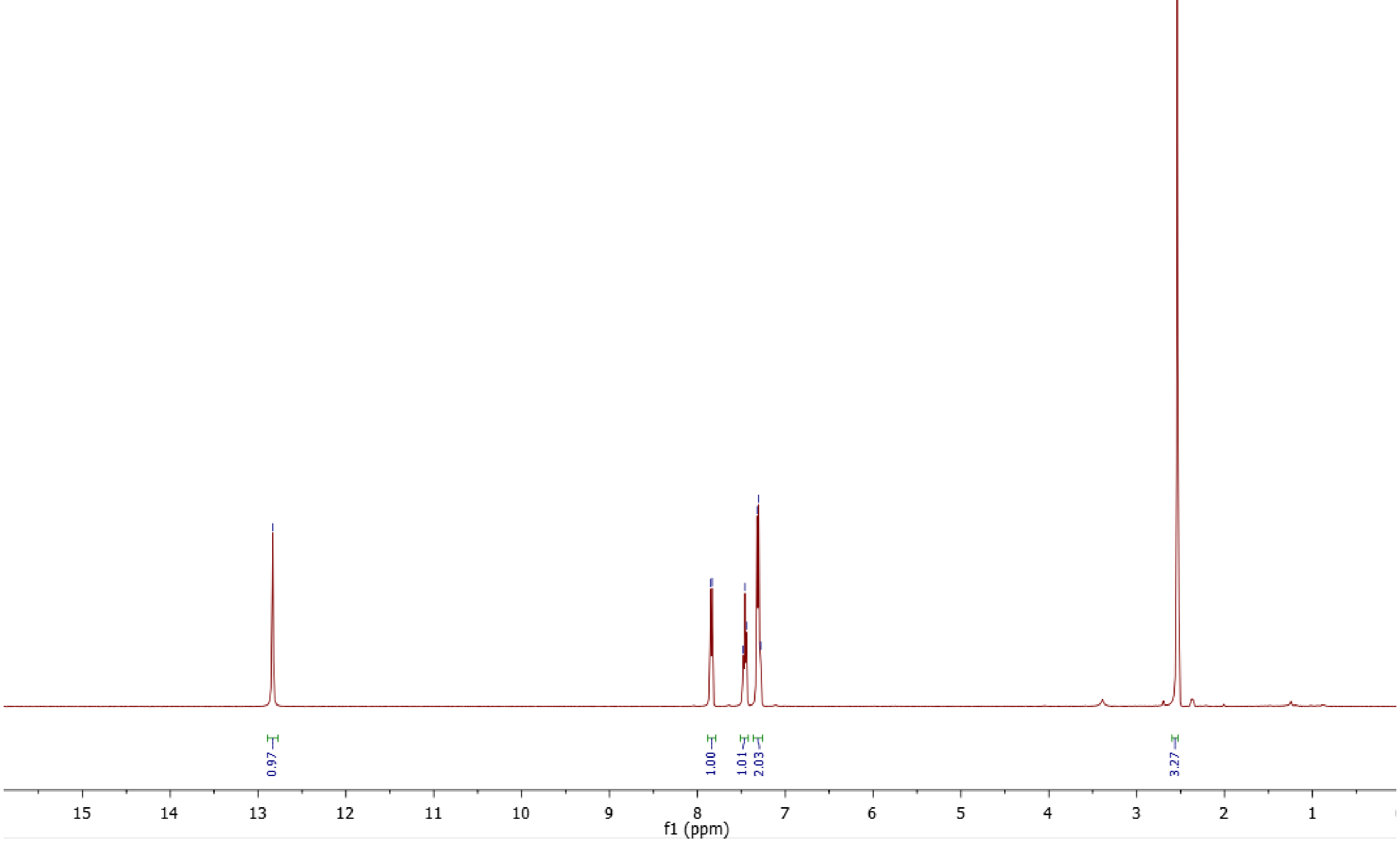

^{13}C NMR

2n

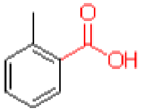

2-methylbenzoic acid

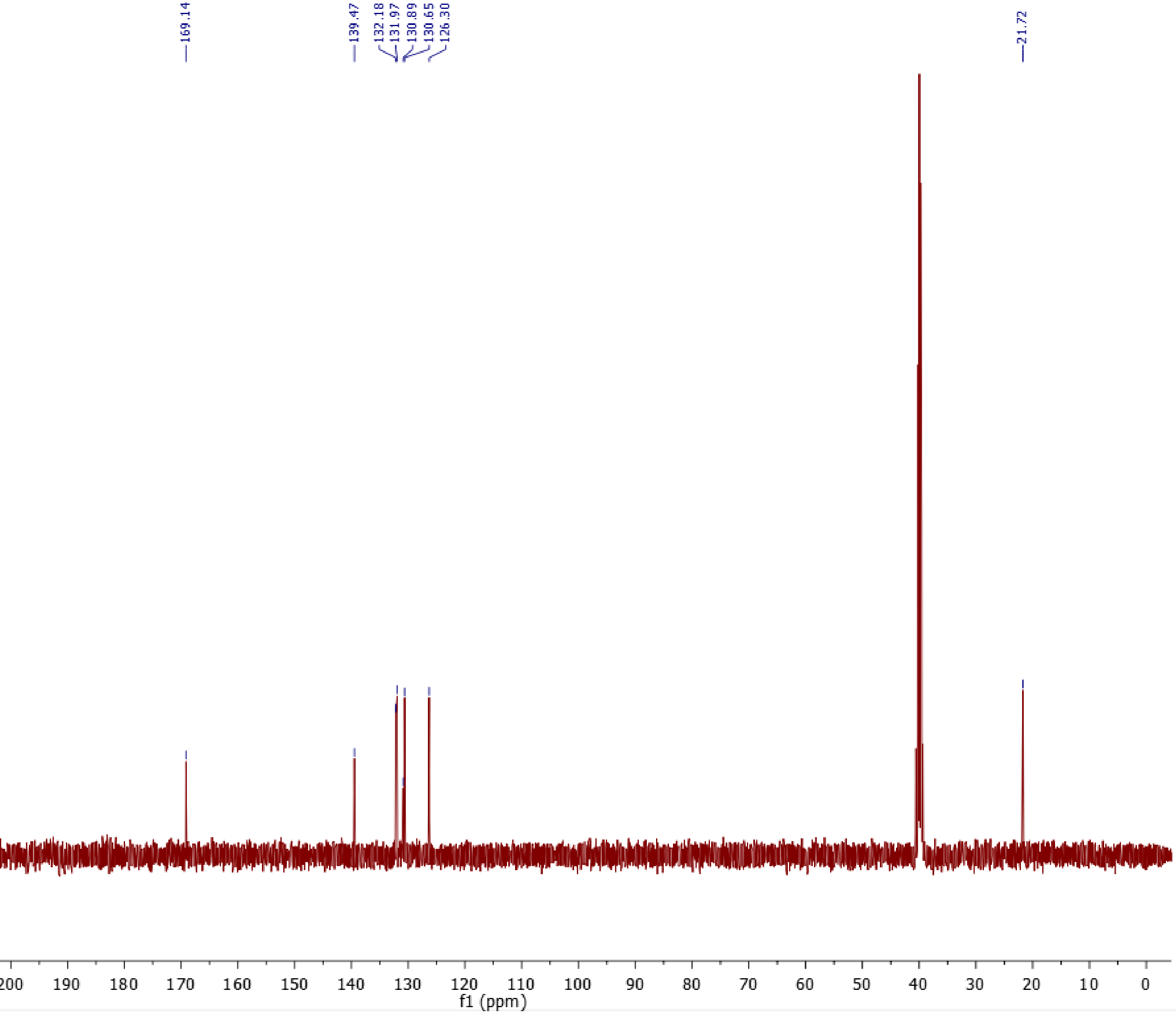

^{1}H NMR

2o

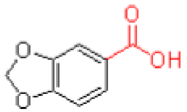

benzo[*d*][1,3]dioxole-5-carboxylic acid

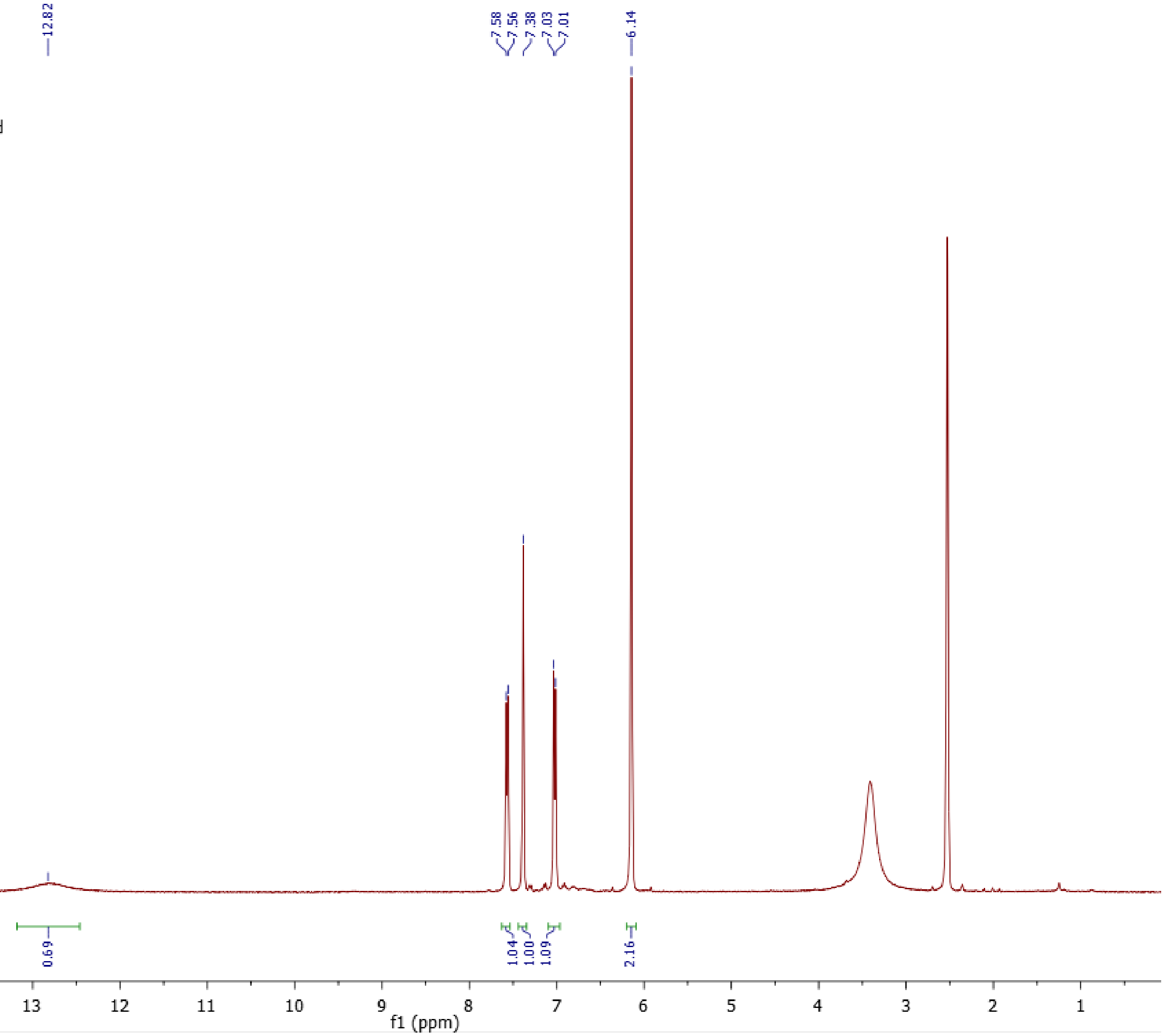

^{13}C NMR

2o

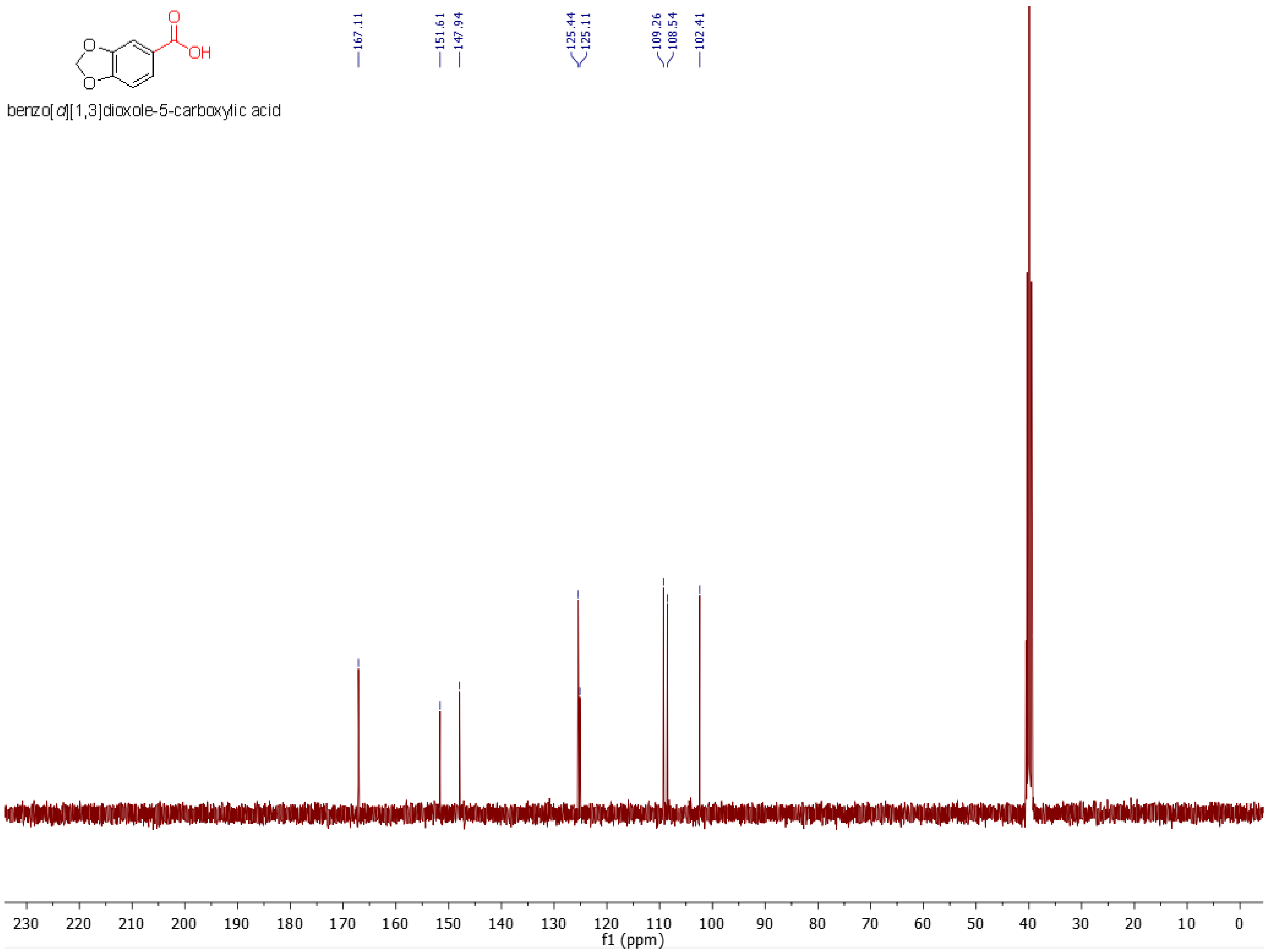
O
OH
O
O
benzo[d][1,3]dioxole-5-carboxylic acid
167.11
151.61
147.94
125.44
125.11
109.26
108.54
102.41
230
220
210
200
190
180
170
160
150
140
130
120
110
100
90
80
70
60
50
40
30
20
10
0
f1 (ppm)

^{1}H NMR

2p

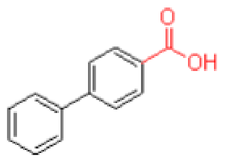

[1,1'-biphenyl]-4-carboxylic acid

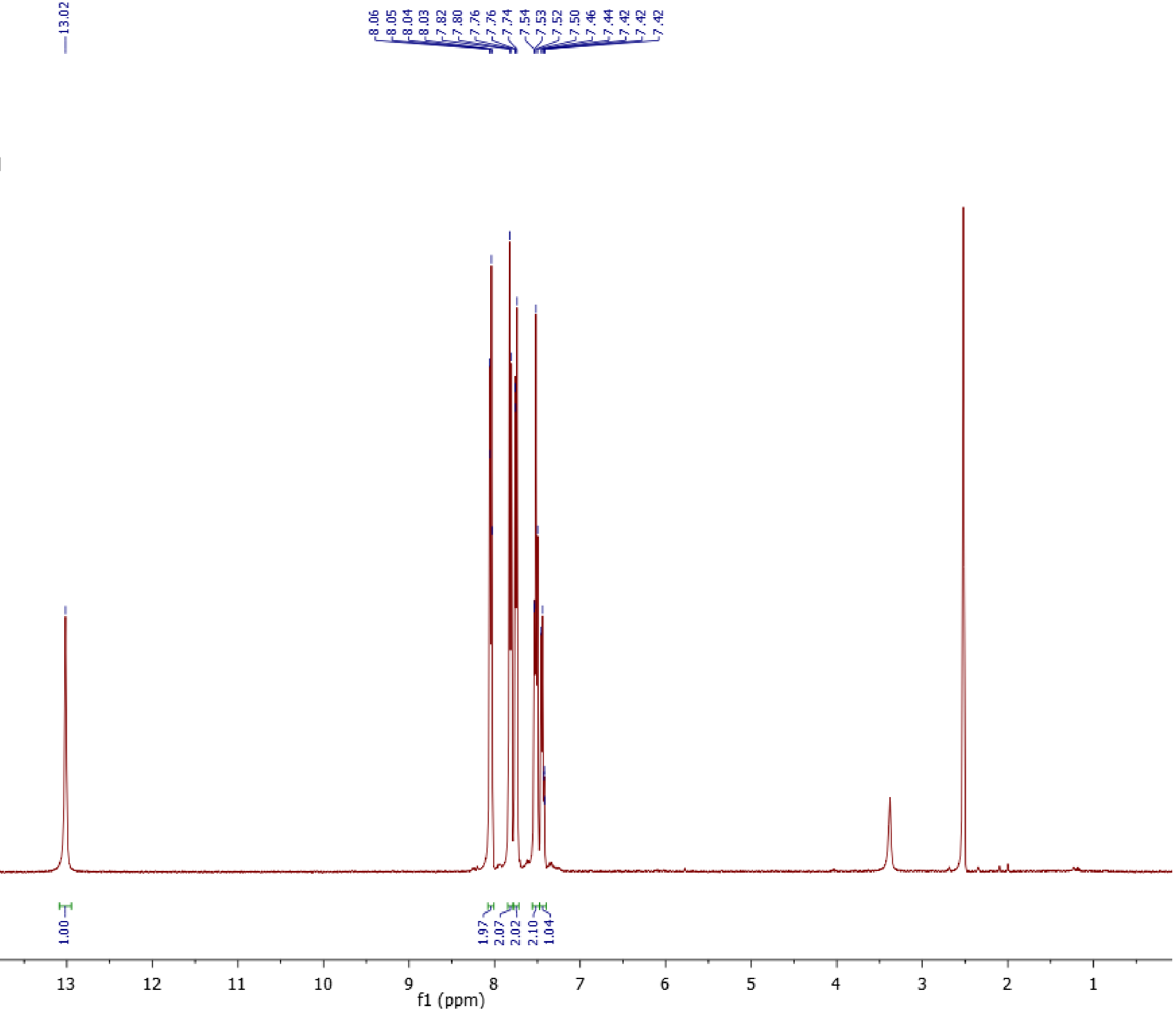

^{13}C NMR

2p

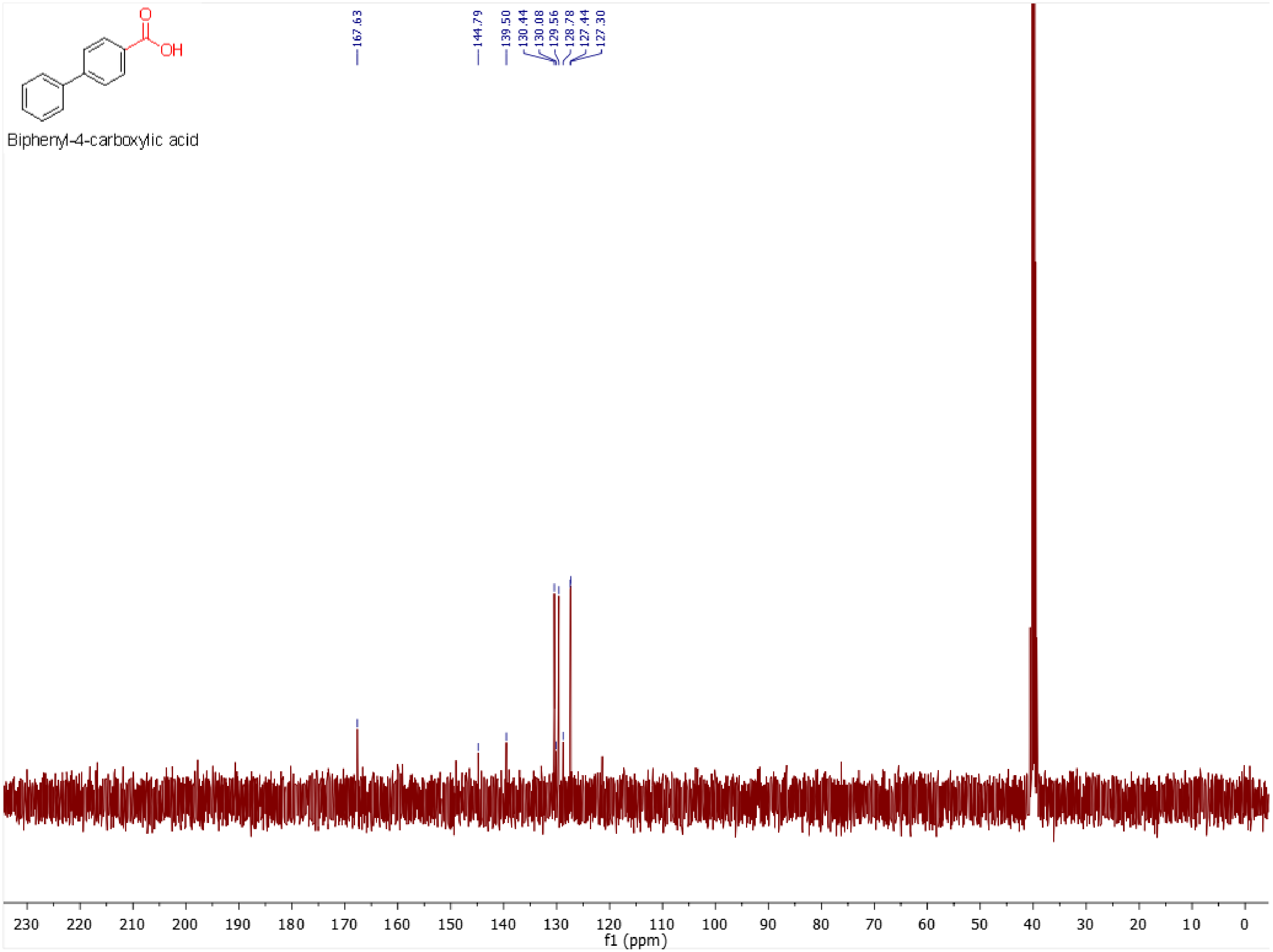

OH
O
Biphenyl-4-carboxylic acid
167.63
144.79
139.50
130.44
130.08
129.56
128.78
127.44
127.30
230 220 210 200 190 180 170 160 150 140 130 120 110 100 90 80 70 60 50 40 30 20 10 0
f1 (ppm)

•

^{1}H NMR

2q

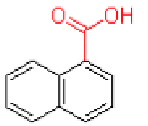

1-naphthoic acid

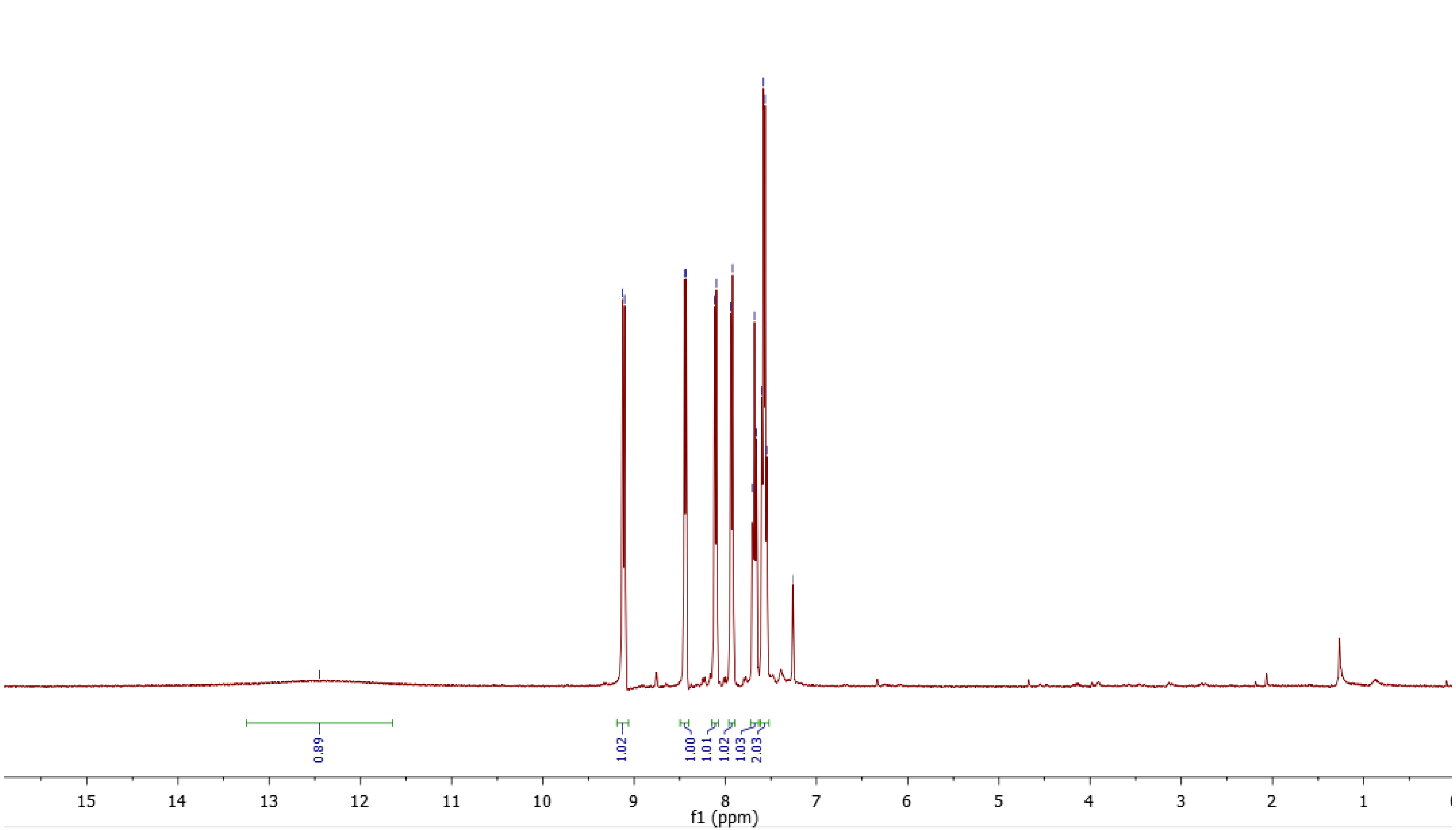

^{13}C NMR

2q

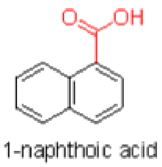
O
OH
1-naphthoic acid

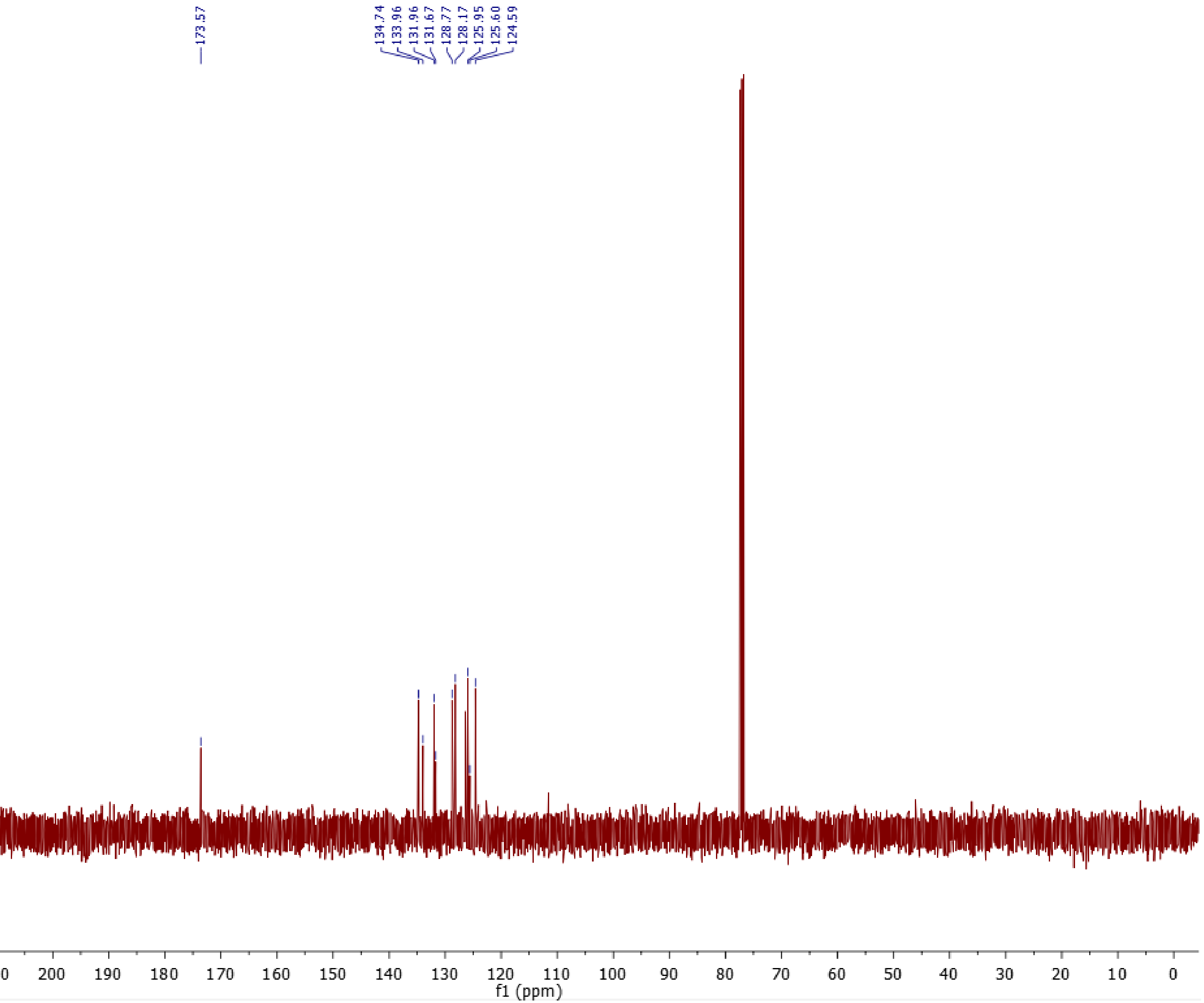
173.57
134.74
133.96
131.96
131.67
128.77
128.17
125.95
125.60
124.59
230
220
210
200
190
180
170
160
150
140
130
120
110
100
90
80
70
60
50
40
30
20
10
0
f1 (ppm)

^{1}H NMR

2r

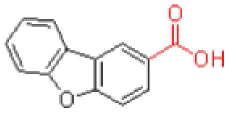
OH
O
O
dibenzo[b,d]furan-2-carboxylic acid

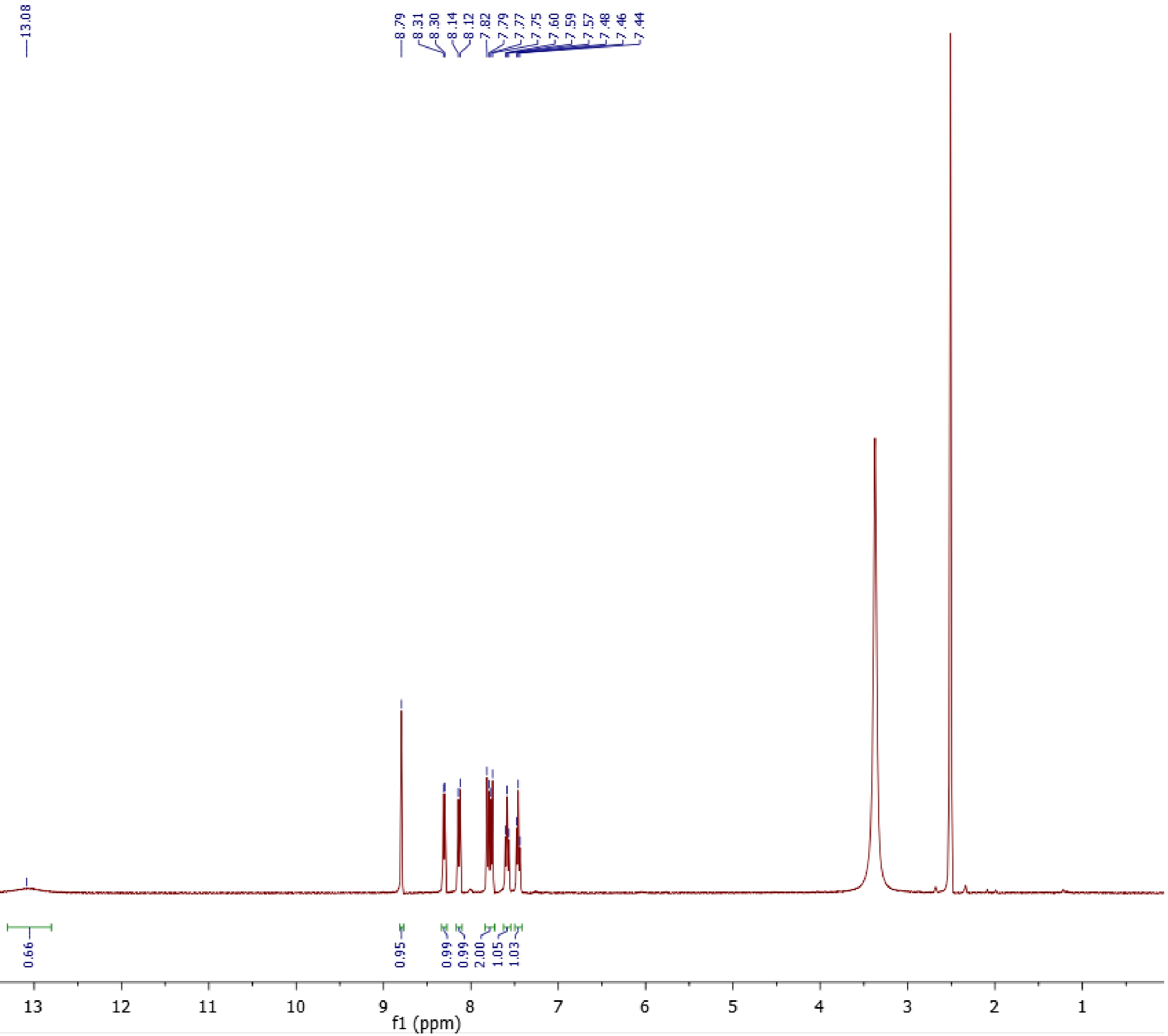
13.08
8.79
8.31
8.30
8.14
8.12
7.82
7.79
7.77
7.75
7.60
7.59
7.57
7.48
7.46
7.44
0.66
0.95
0.99
0.99
2.00
1.05
1.03
16
15
14
13
12
11
10
9
8
7
6
5
4
3
2
1
f1 (ppm)

^{13}C NMR

2r

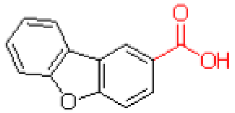
dibenzo[*b,d*]furan-2-carboxylic acid

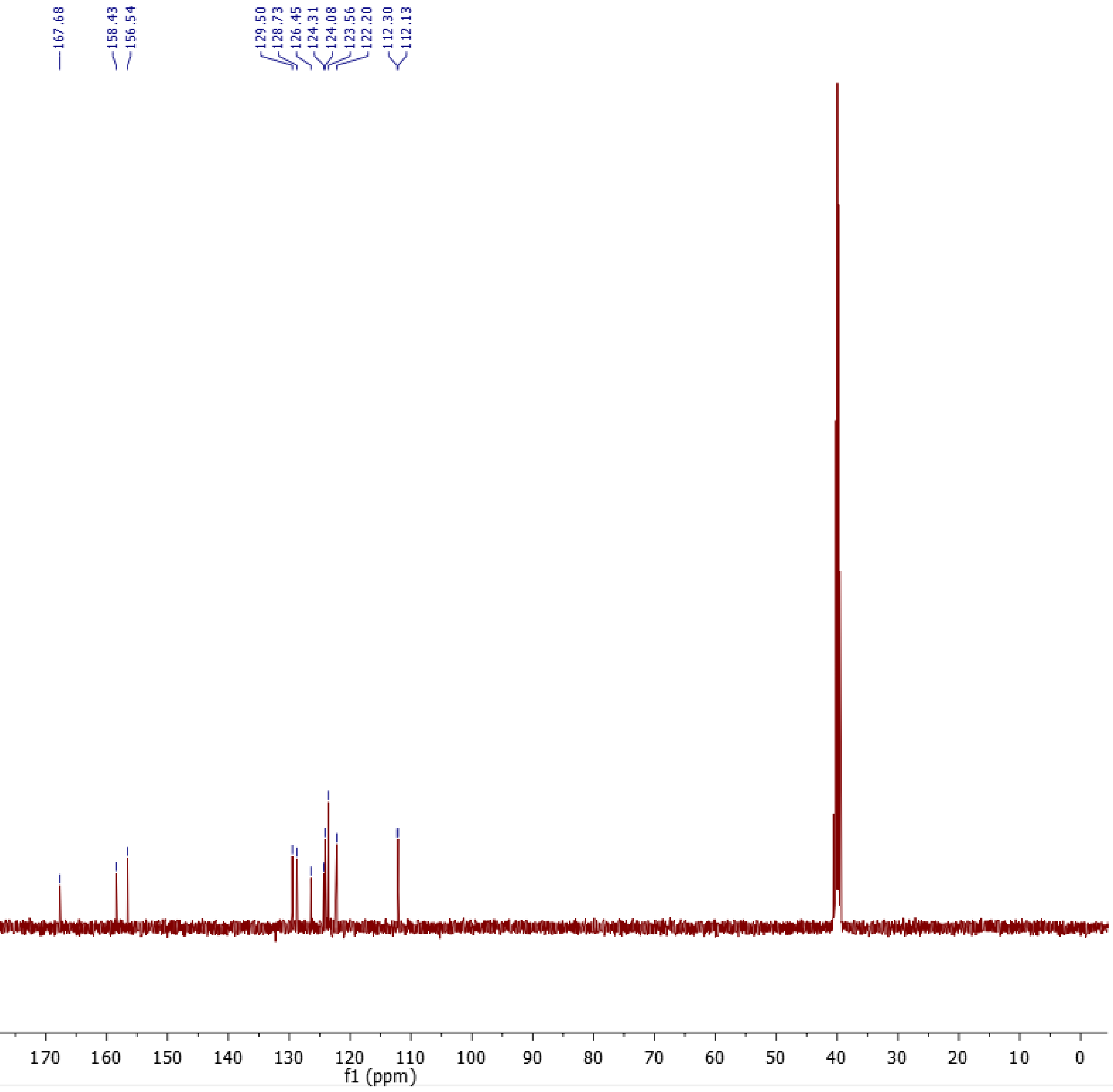

^{1}H NMR

2s

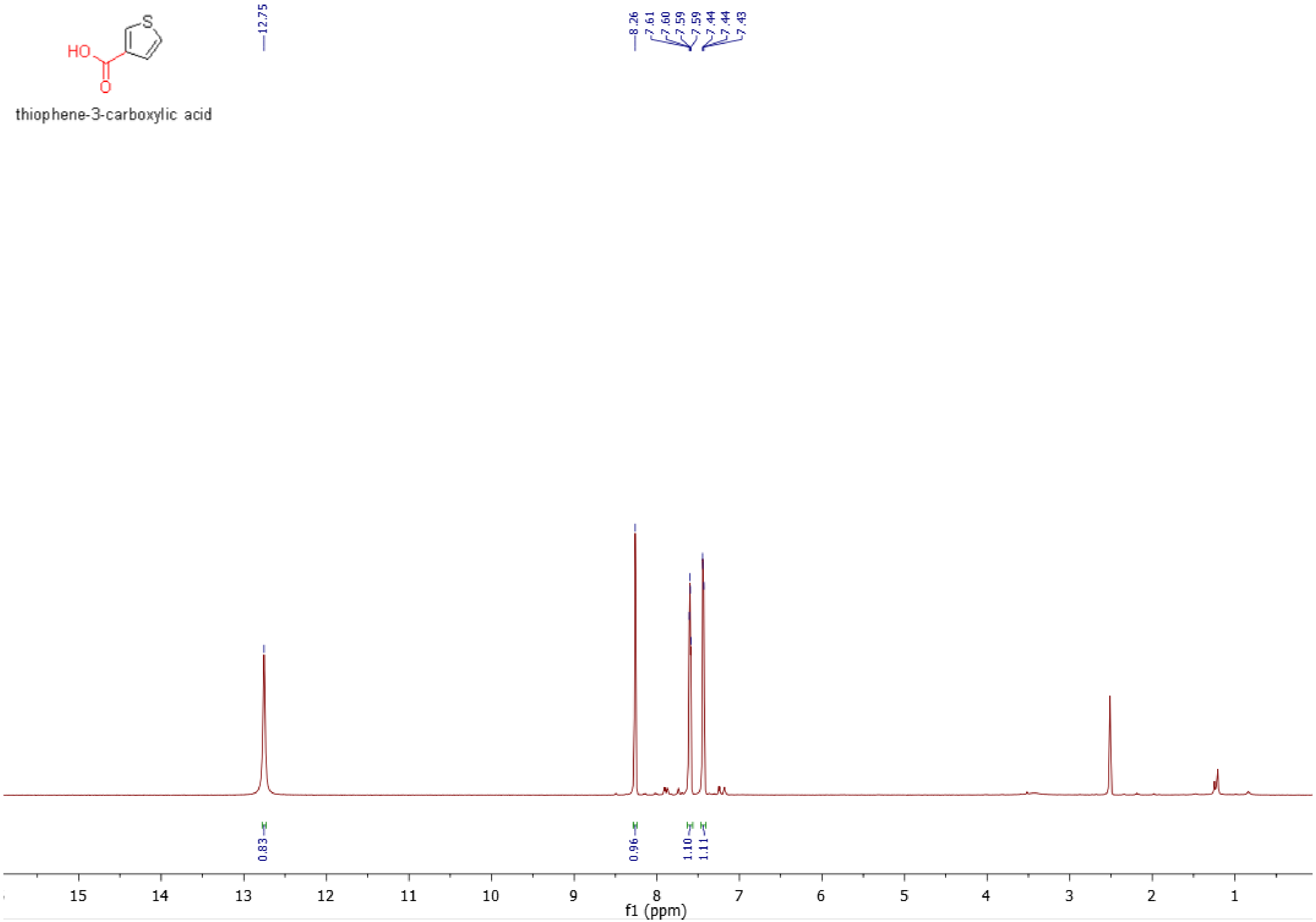
HO
S
O
thiophene-3-carboxylic acid
12.75
8.26
7.61
7.60
7.59
7.59
7.44
7.44
7.43
0.83
0.96
1.10
1.11
15
14
13
12
11
10
9
8
7
6
5
4
3
2
1
f1 (ppm)

^{13}C NMR

2s

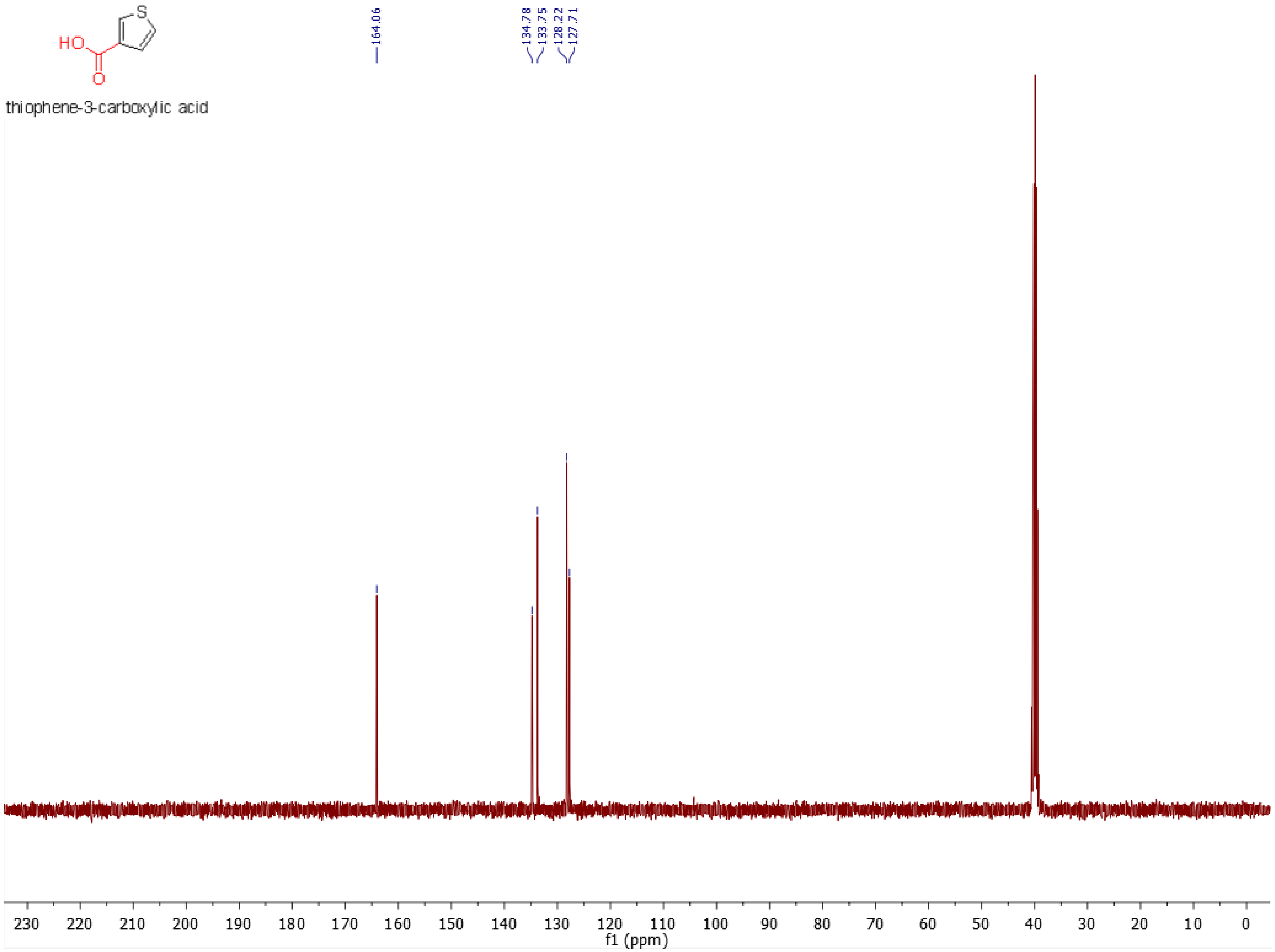
HO
O
S
thiophene-3-carboxylic acid
164.06
134.78
133.75
128.22
127.71
230 220 210 200 190 180 170 160 150 140 130 120 110 100 90 80 70 60 50 40 30 20 10 0
f1 (ppm)

N CAS 488-93-7

^{1}H NMR

2t

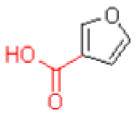

furan-3-carboxylic acid

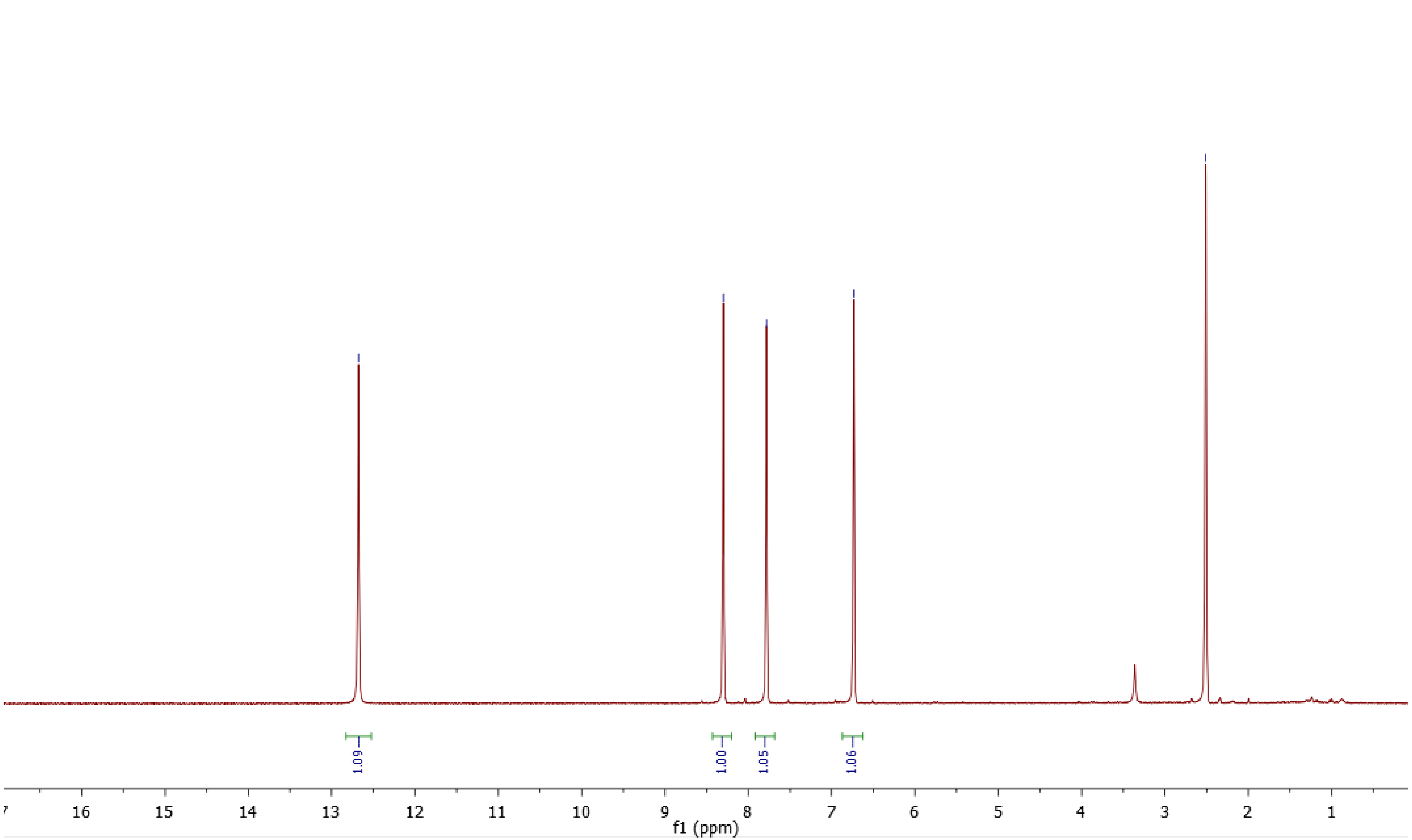

^{13}C NMR

2t

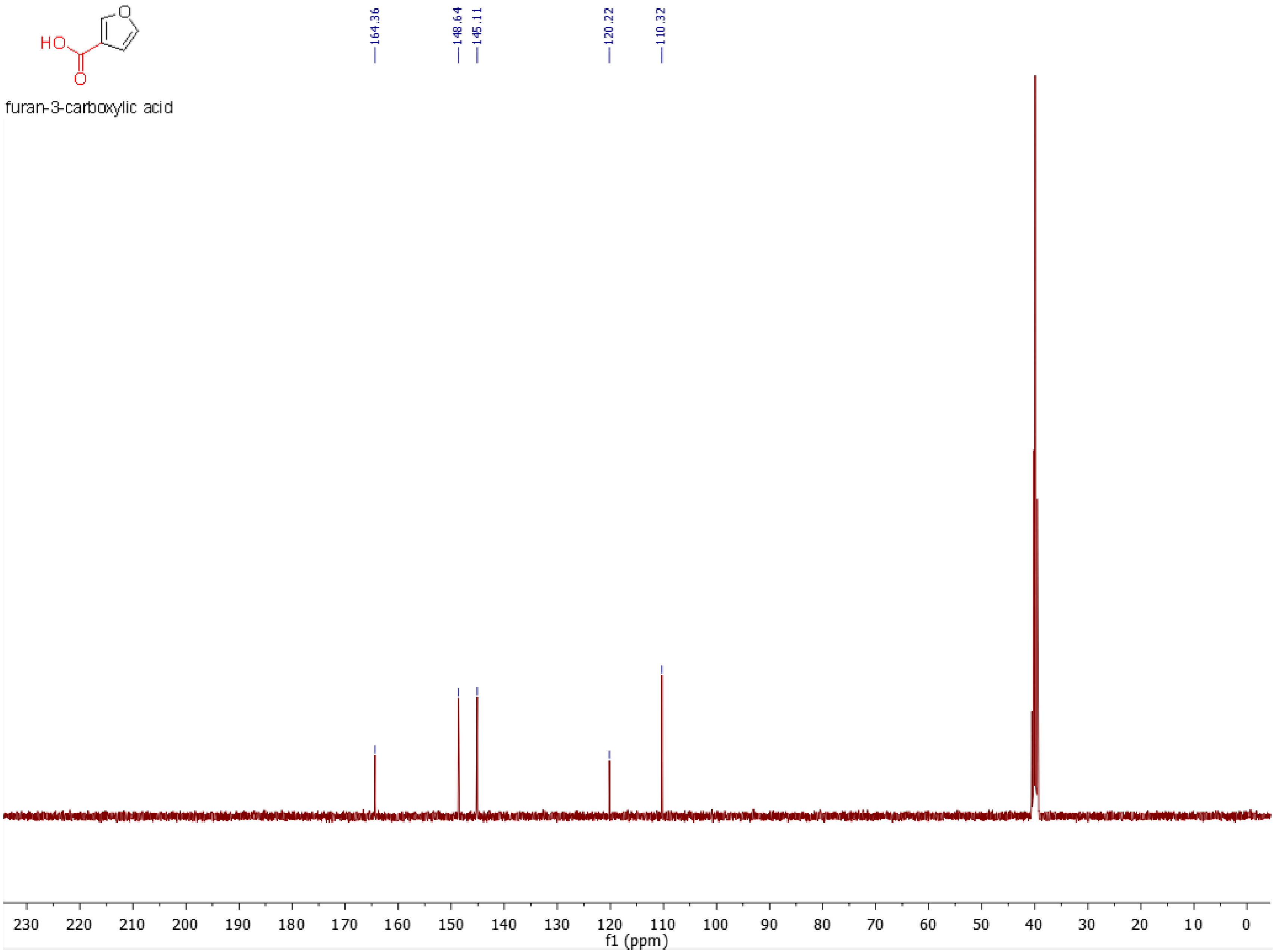
HO
O
O
furan-3-carboxylic acid
164.36
148.64
145.11
120.22
110.32
230
220
210
200
190
180
170
160
150
140
130
120
110
100
90
80
70
60
50
40
30
20
10
0
f1 (ppm)

^{1}H NMR

2u

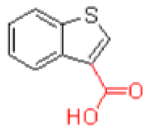
benzo[b]thiophene-3-carboxylic acid

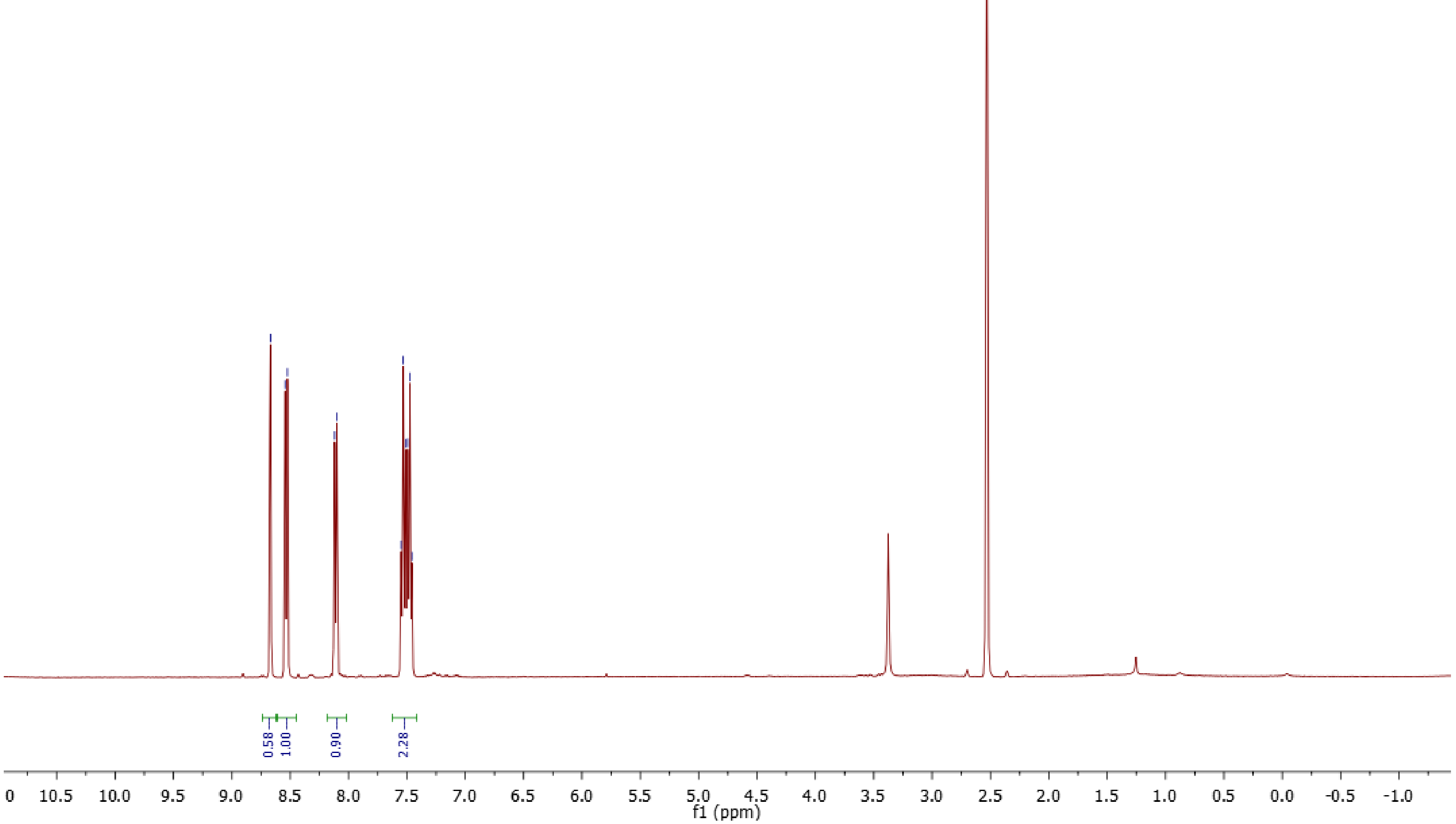
8.67
8.54
8.52
8.12
8.10
7.55
7.53
7.51
7.49
7.47
7.46
0.58
1.00
0.90
2.28
f1 (ppm)

^{13}C NMR

2u

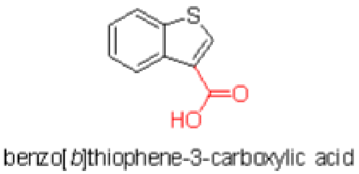
S
HO
O
benzo[b]thiophene-3-carboxylic acid

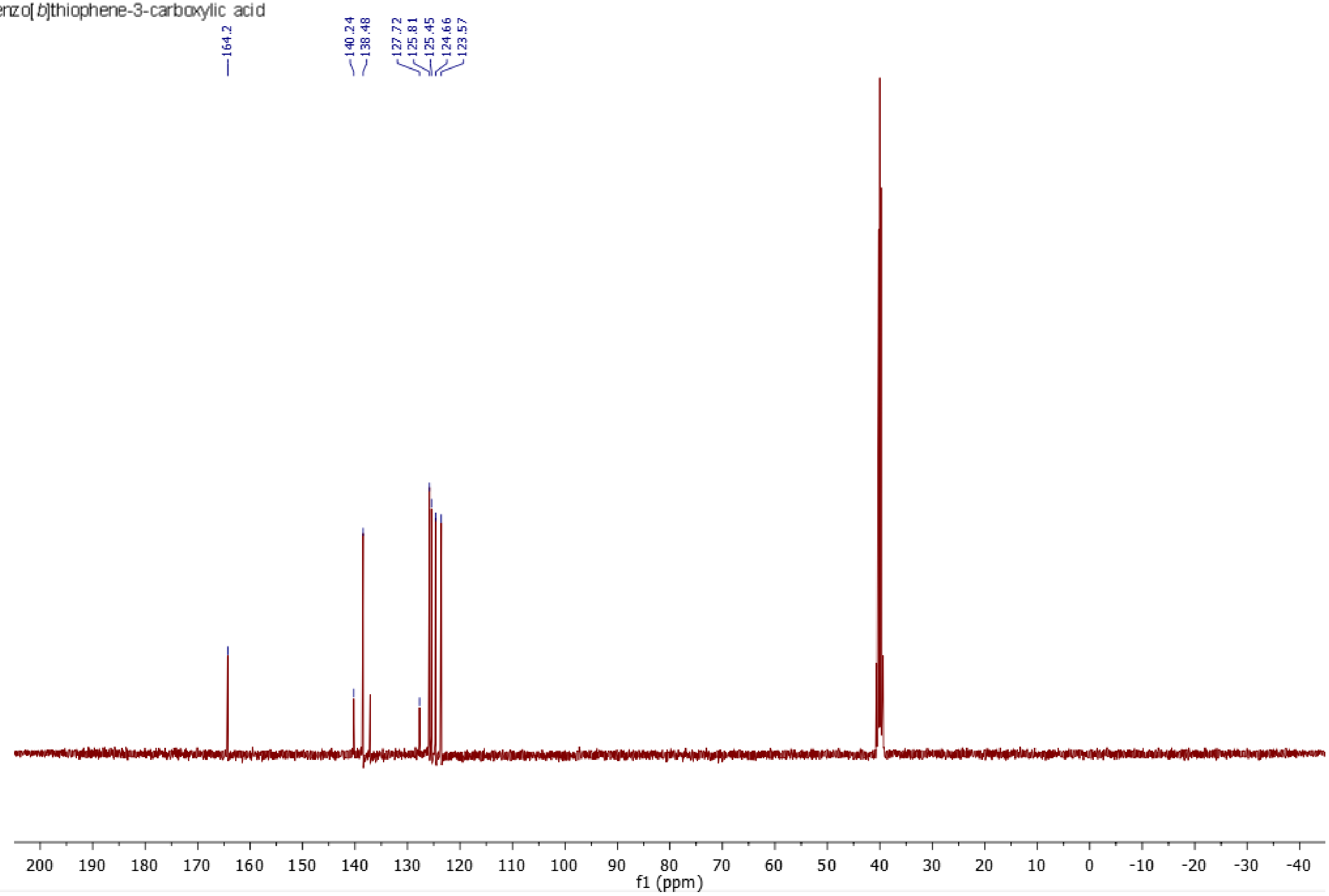
164.2
140.24
138.48
127.72
125.81
125.45
124.66
123.57
200 190 180 170 160 150 140 130 120 110 100 90 80 70 60 50 40 30 20 10 0 -10 -20 -30 -40
f1 (ppm)

^{1}H NMR

2v

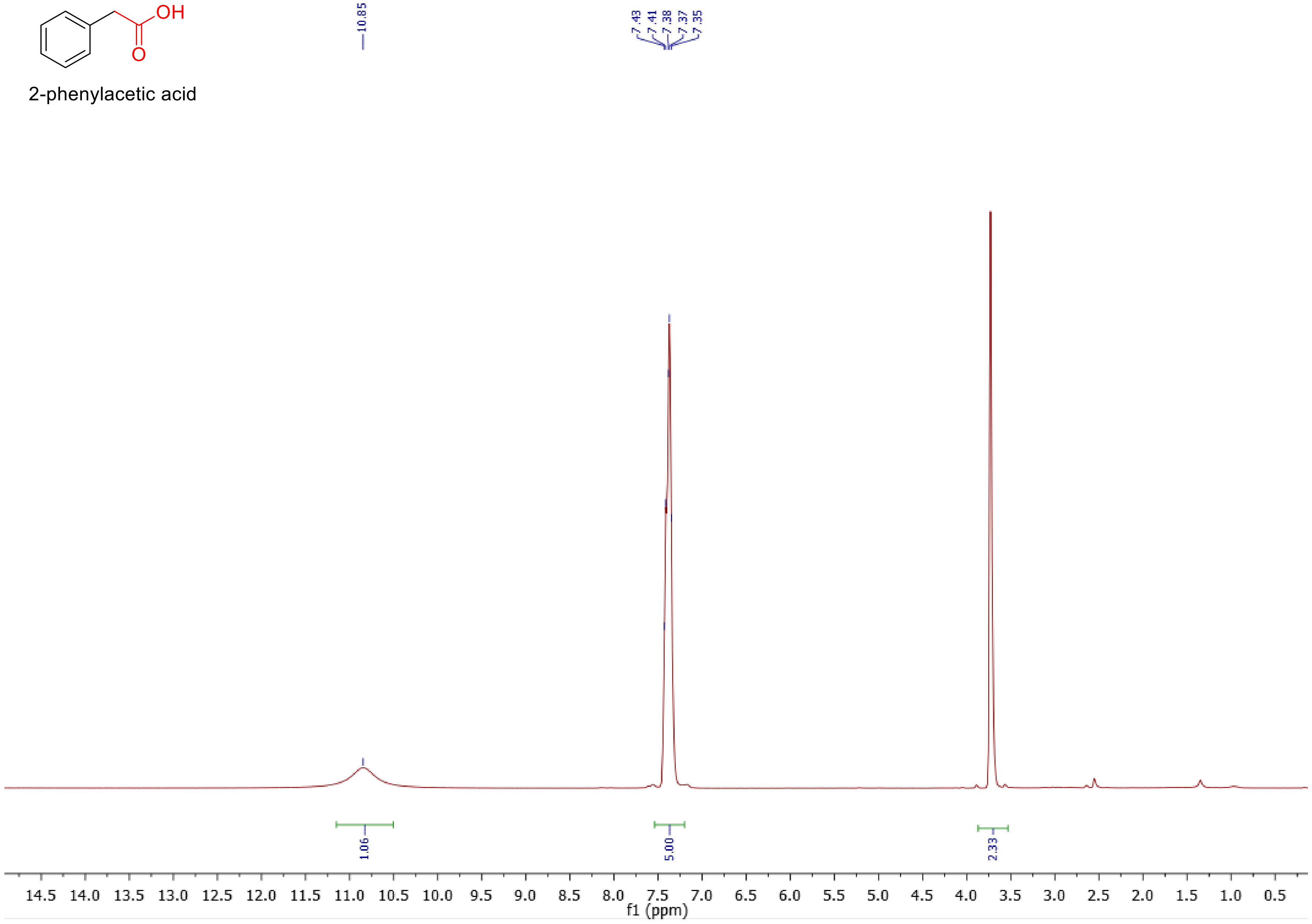
OH
O
2-phenylacetic acid
10.85
7.43
7.41
7.38
7.37
7.35
1.06
5.00
2.33
14.5 14.0 13.5 13.0 12.5 12.0 11.5 11.0 10.5 10.0 9.5 9.0 8.5 8.0 7.5 7.0 6.5 6.0 5.5 5.0 4.5 4.0 3.5 3.0 2.5 2.0 1.5 1.0 0.5
f1 (ppm)

^{13}C NMR

2v

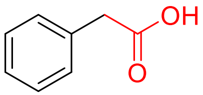

2-phenylacetic acid

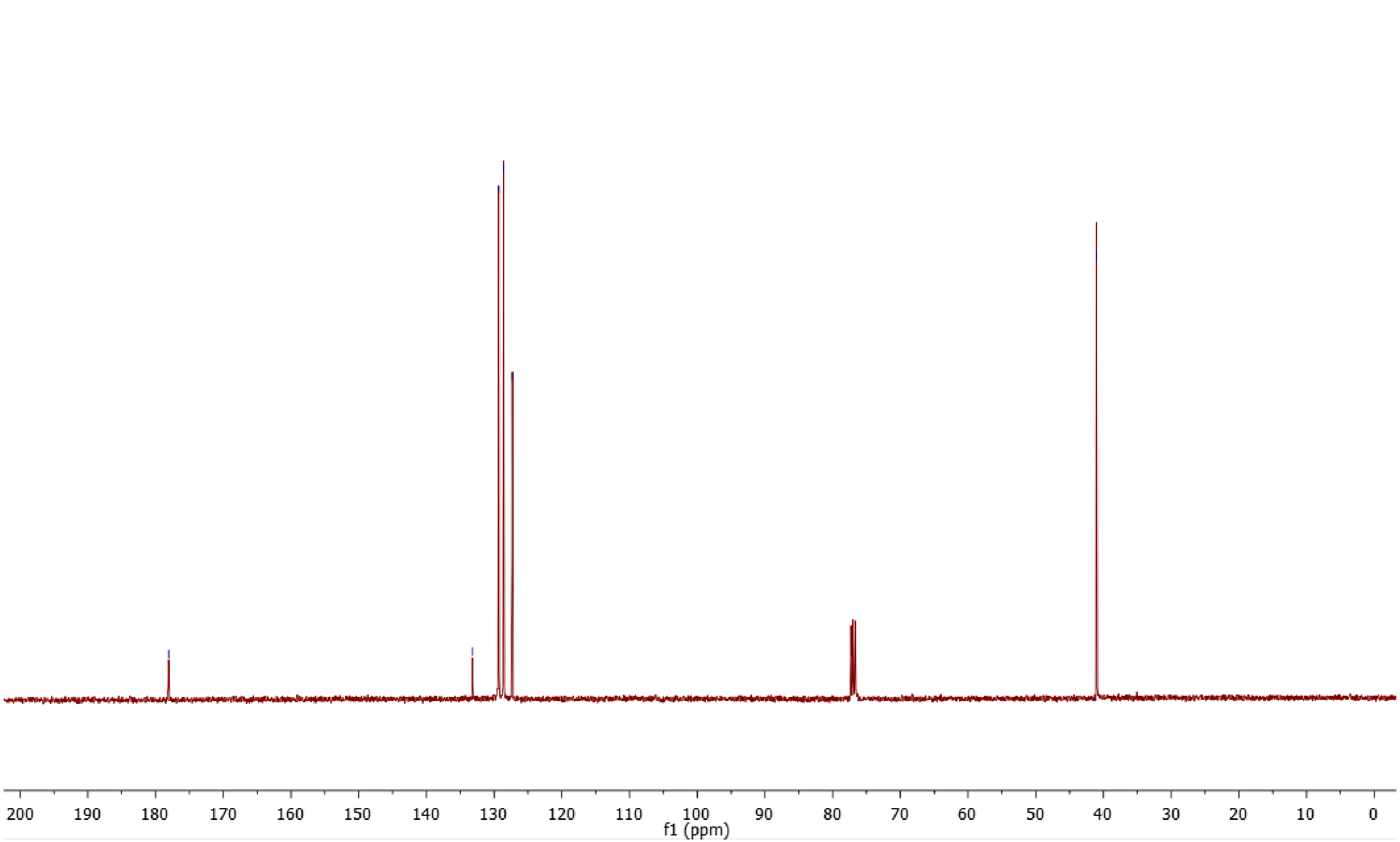

^{1}H NMR

2w

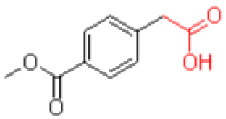

2-(4-(methoxycarbonyl)phenyl)acetic acid

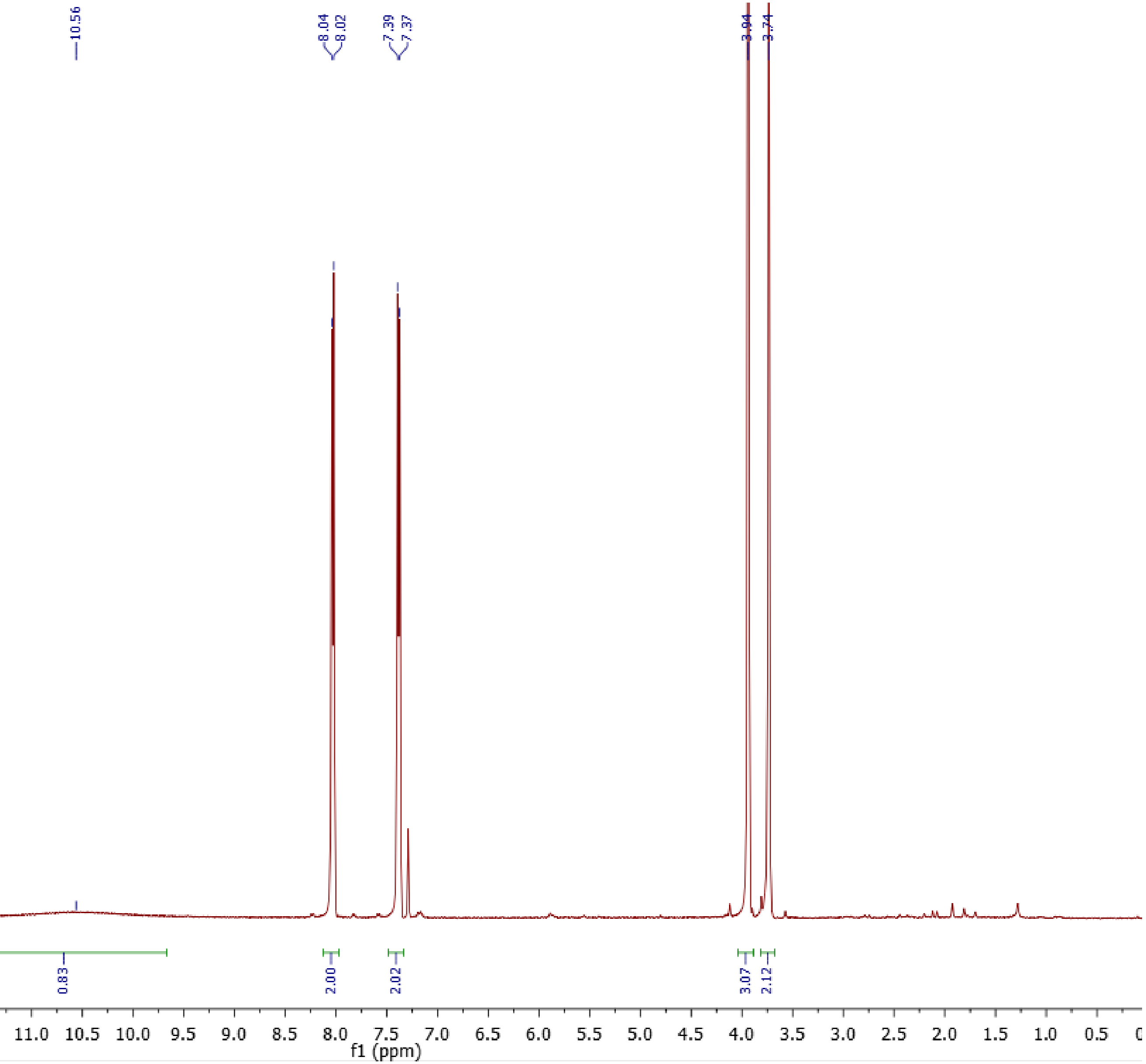

^{13}C NMR

2w

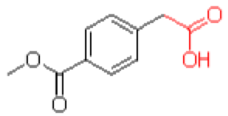

2-(4-(methoxycarbonyl)phenyl)acetic acid

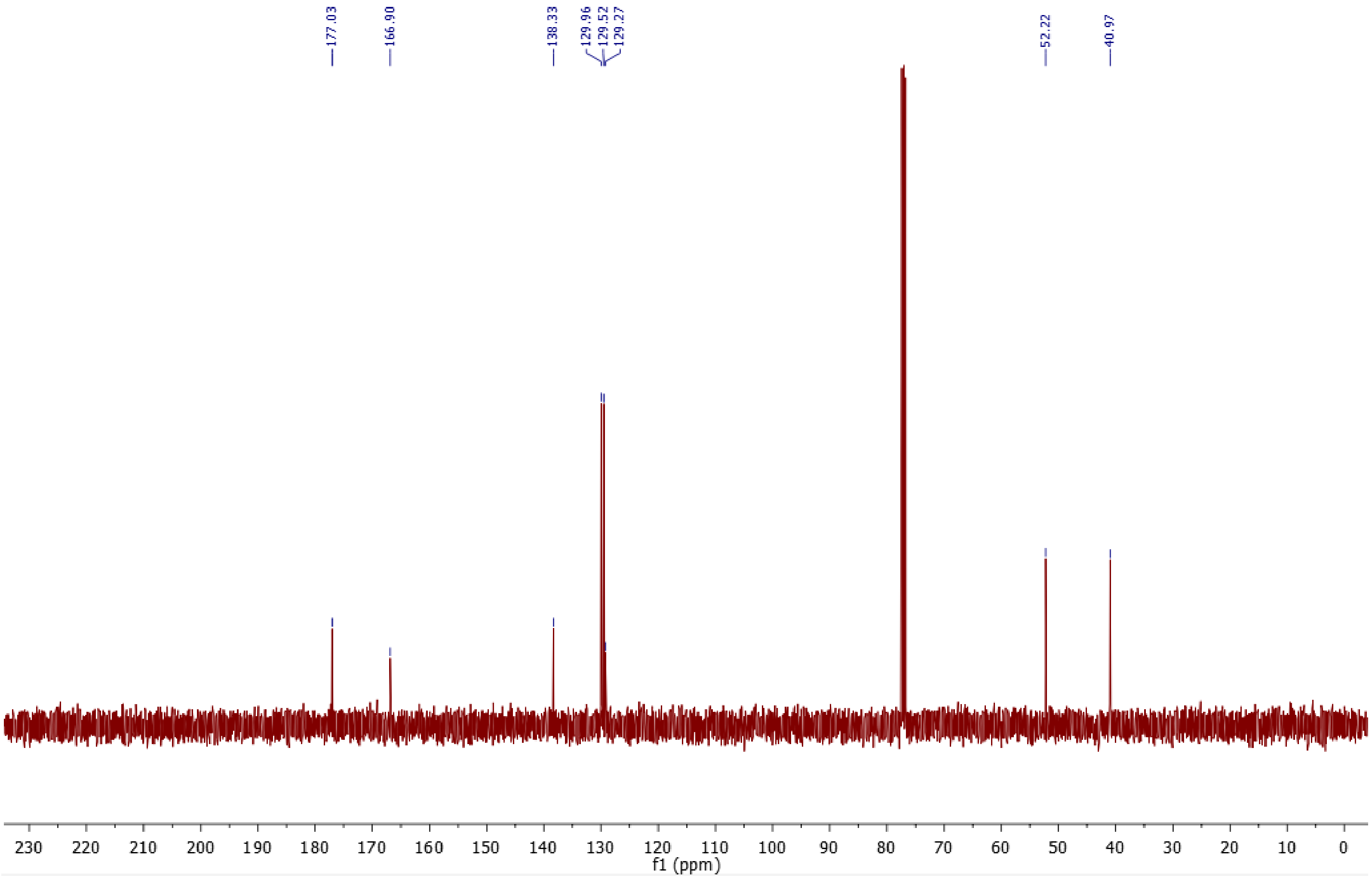

^{1}H NMR

2x

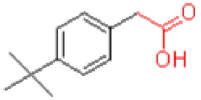
OH
O
2-(4-(tert-butyl)phenyl)acetic acid

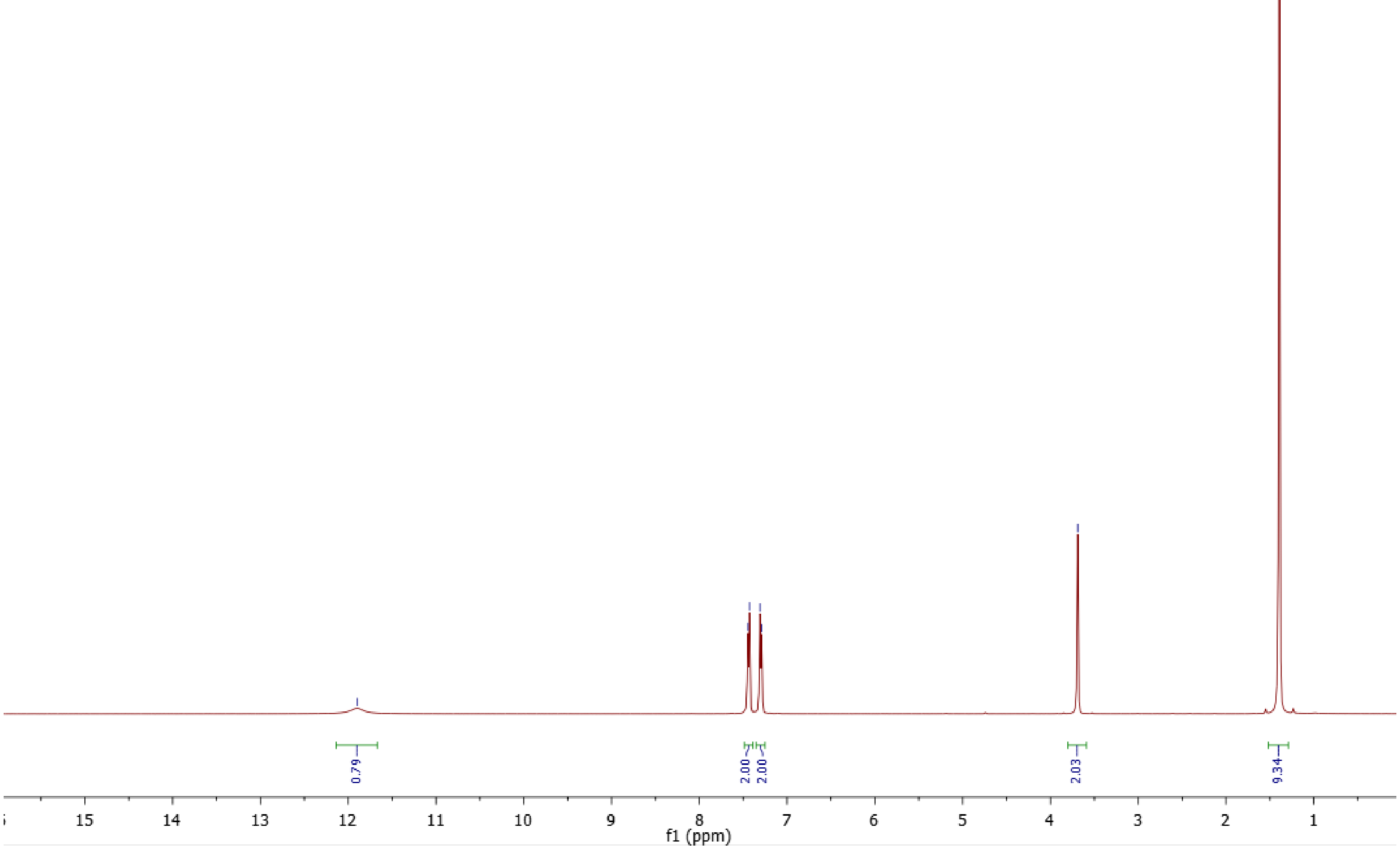
11.90
7.45
7.43
7.31
7.29
3.69
1.39
0.79
2.00
2.00
2.03
9.34
15
14
13
12
11
10
9
8
7
6
5
4
3
2
1
f1 (ppm)

^{13}C NMR

2x

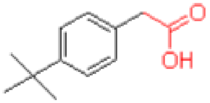

2-(4-(*tert*-butyl)phenyl)acetic acid

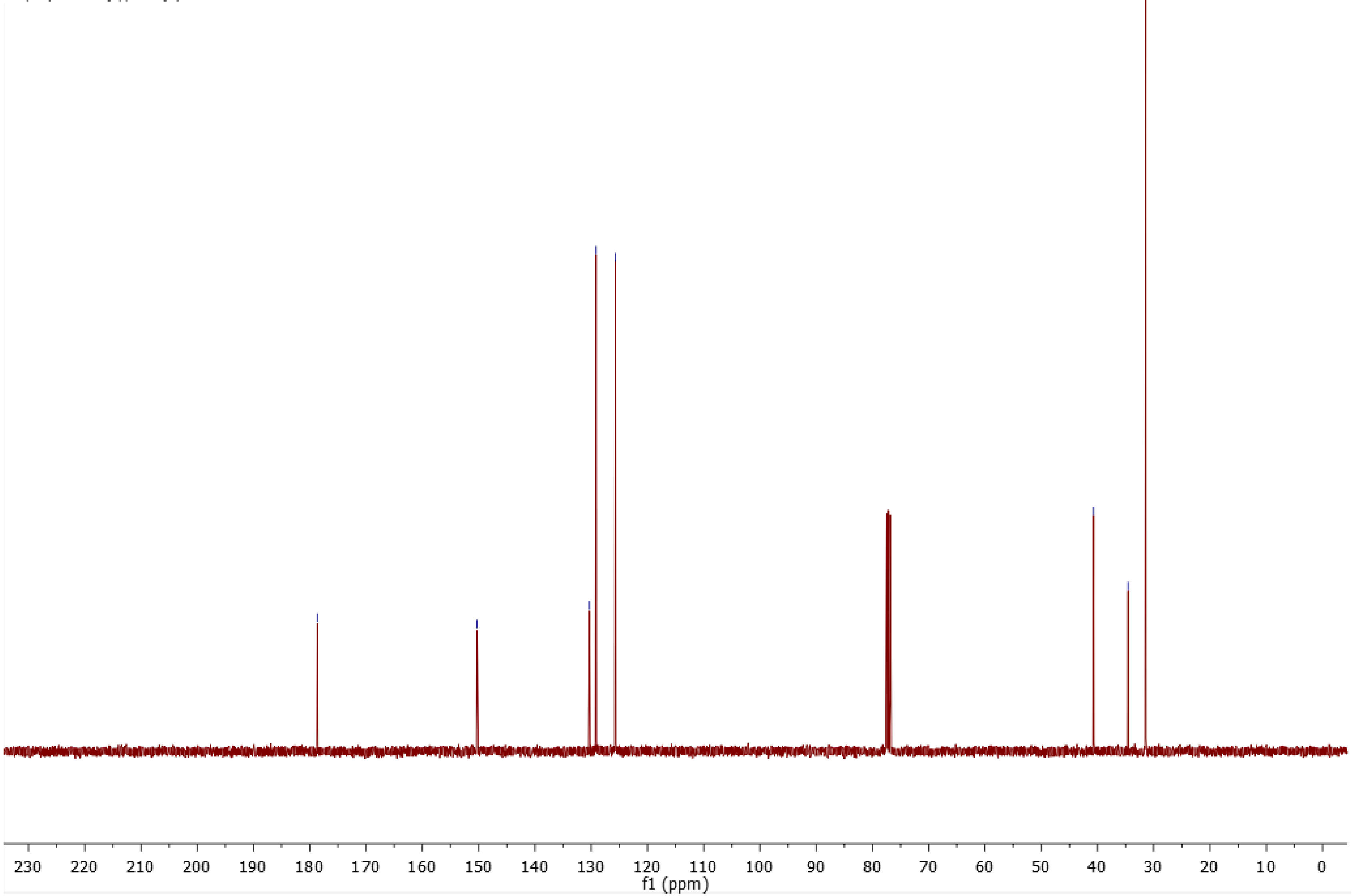

^{1}H NMR

2y

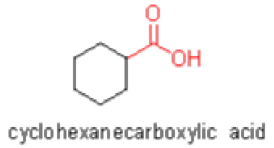
O
OH
cyclohexanecarboxylic acid

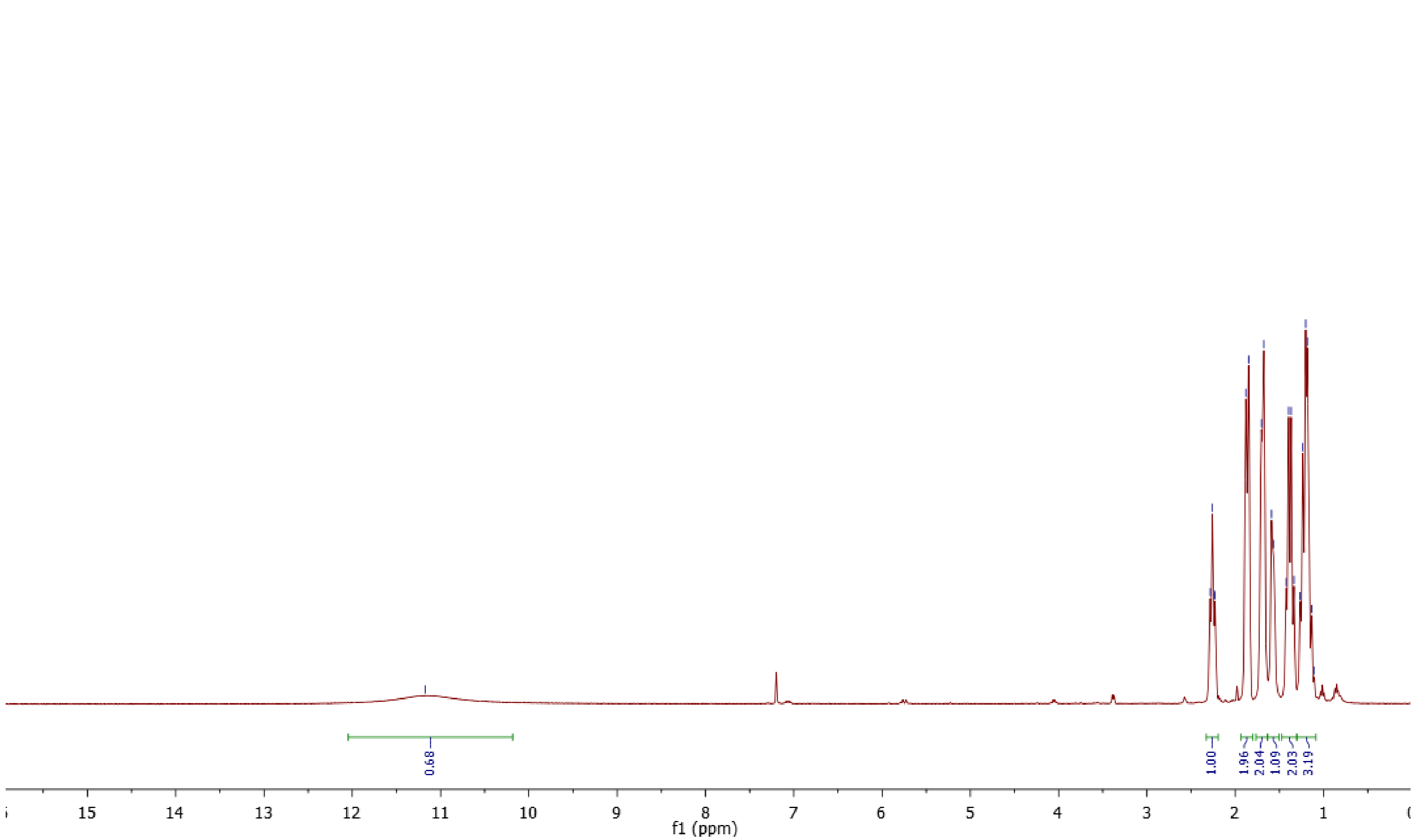
11.18
2.28
2.26
2.23
1.88
1.85
1.70
1.68
1.59
1.57
1.42
1.39
1.37
1.34
1.27
1.24
1.20
1.18
1.13
1.11
0.68
1.00
1.96
2.04
1.09
2.03
3.19
15
14
13
12
11
10
9
8
7
6
5
4
3
2
1
f1 (ppm)

^{13}C NMR

2y

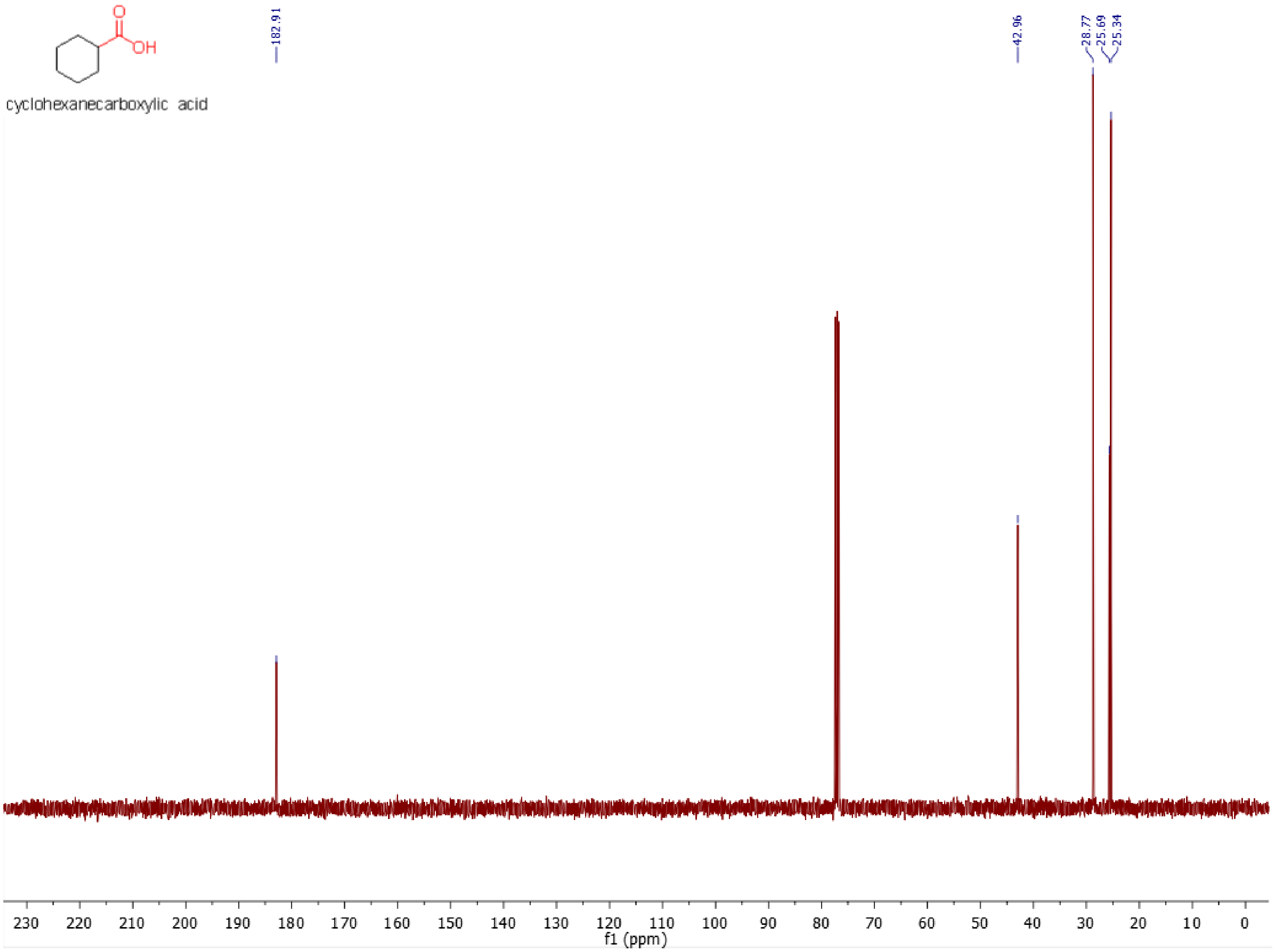

OH
cyclohexanecarboxylic acid
182.91
42.96
28.77
25.69
25.34
230 220 210 200 190 180 170 160 150 140 130 120 110 100 90 80 70 60 50 40 30 20 10 0
f1 (ppm)

^{1}H NMR

2z

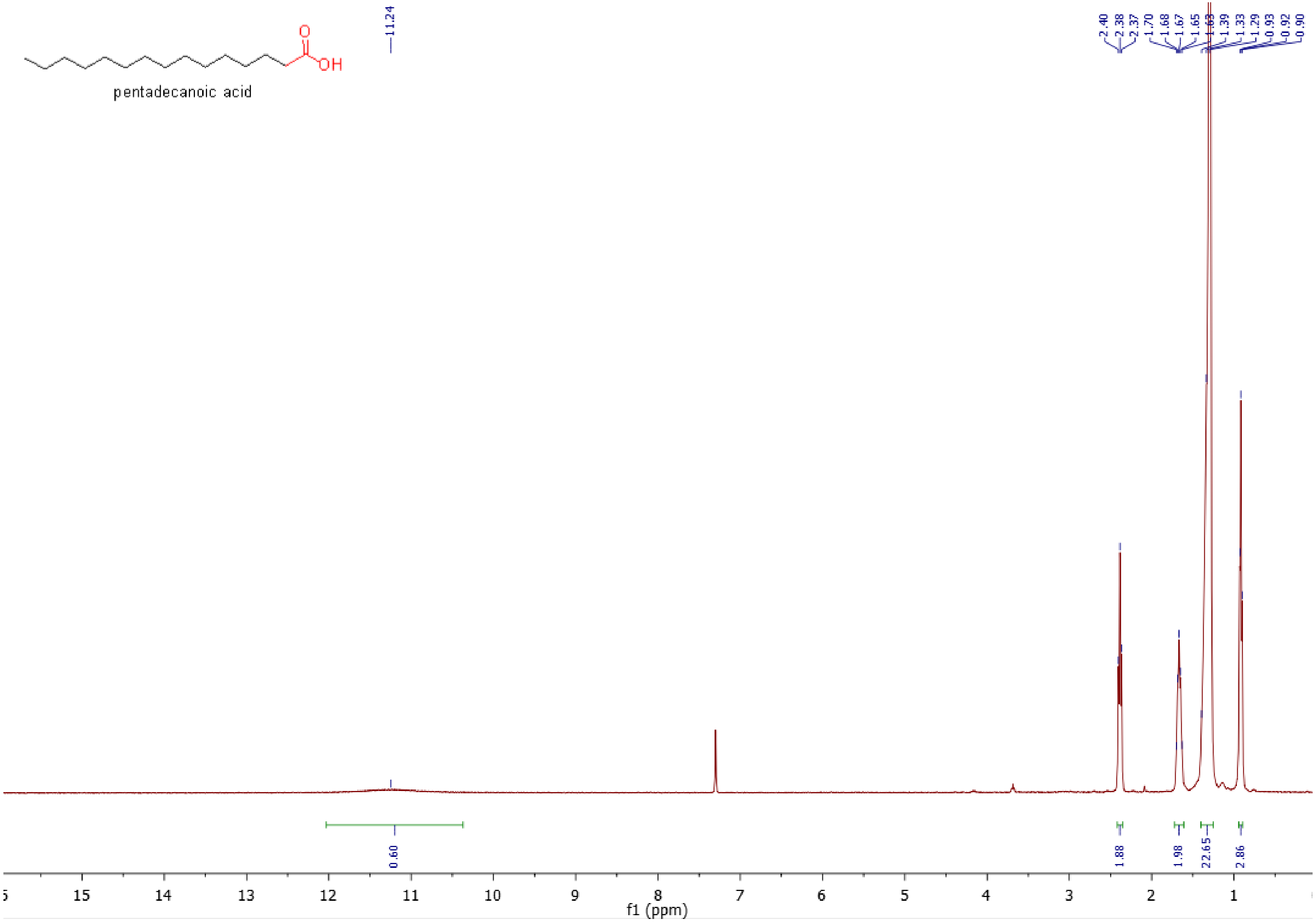
O
OH
pentadecanoic acid
11.24
2.40
2.38
2.37
1.70
1.68
1.67
1.65
1.63
1.39
1.33
1.29
0.93
0.92
0.90
0.60
1.88
1.98
22.65
2.86
15
14
13
12
11
10
9
8
7
6
5
4
3
2
1
f1 (ppm)

^{13}C NMR

2z

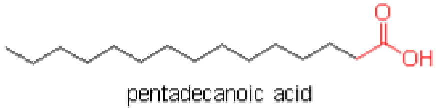
O
OH
pentadecanoic acid

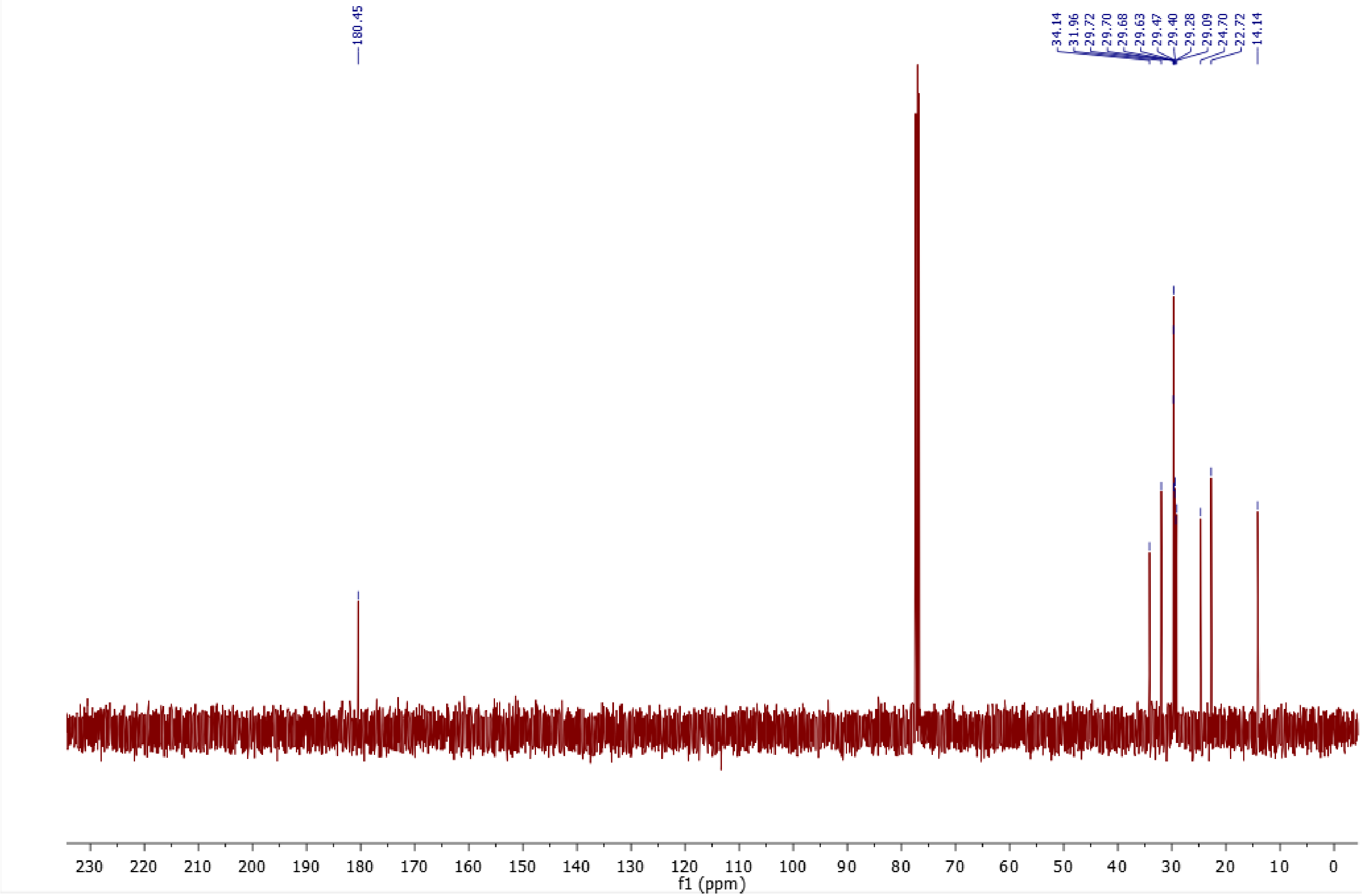
180.45
34.14
31.96
29.72
29.70
29.68
29.63
29.47
29.40
29.28
29.09
24.70
22.72
14.14
230 220 210 200 190 180 170 160 150 140 130 120 110 100 90 80 70 60 50 40 30 20 10 0
f1 (ppm)

^{1}H NMR

5

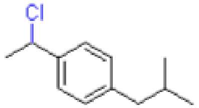

1-Chloro-1-(4-isobutylphenyl)ethane

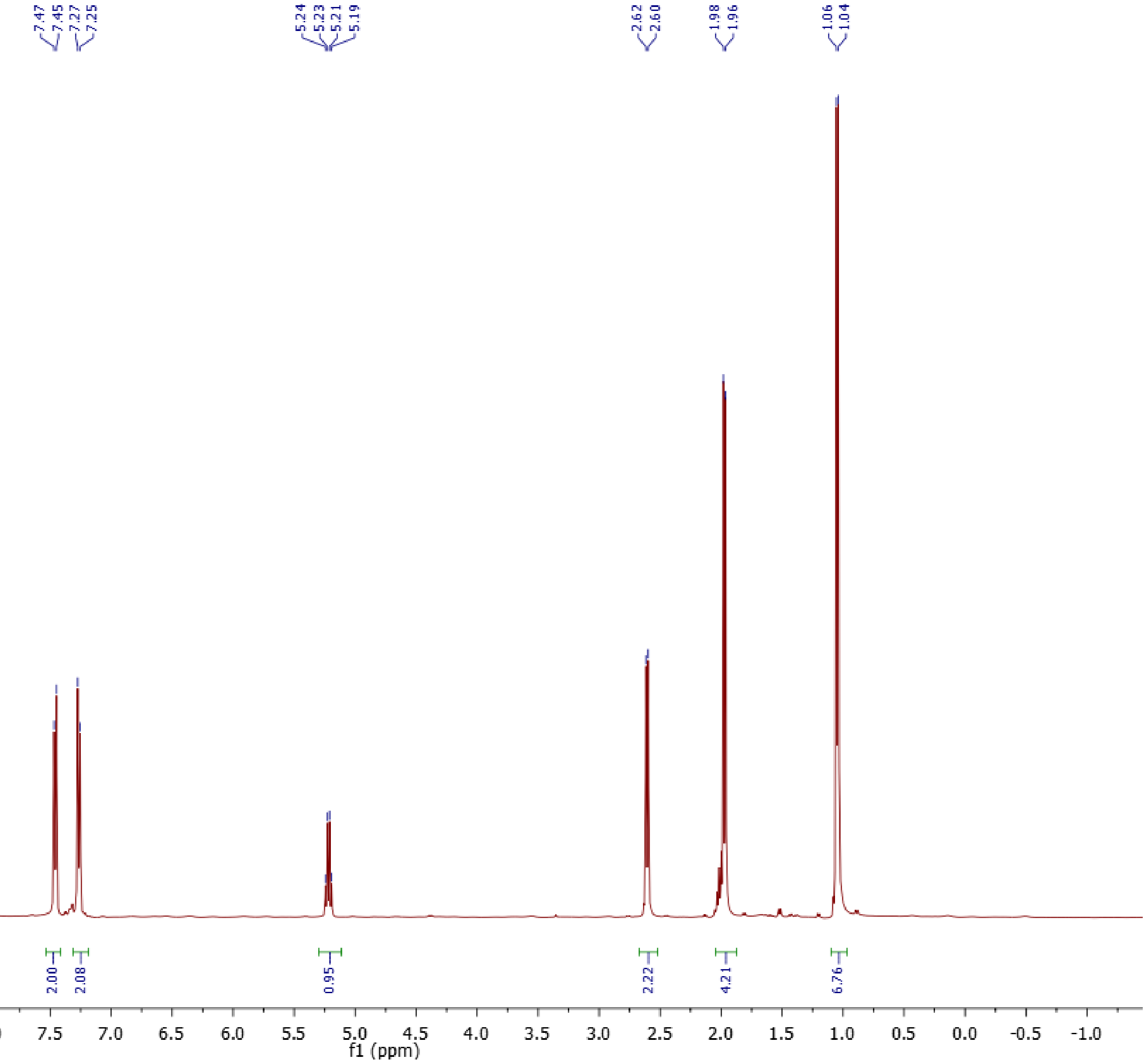

^{13}C NMR

5

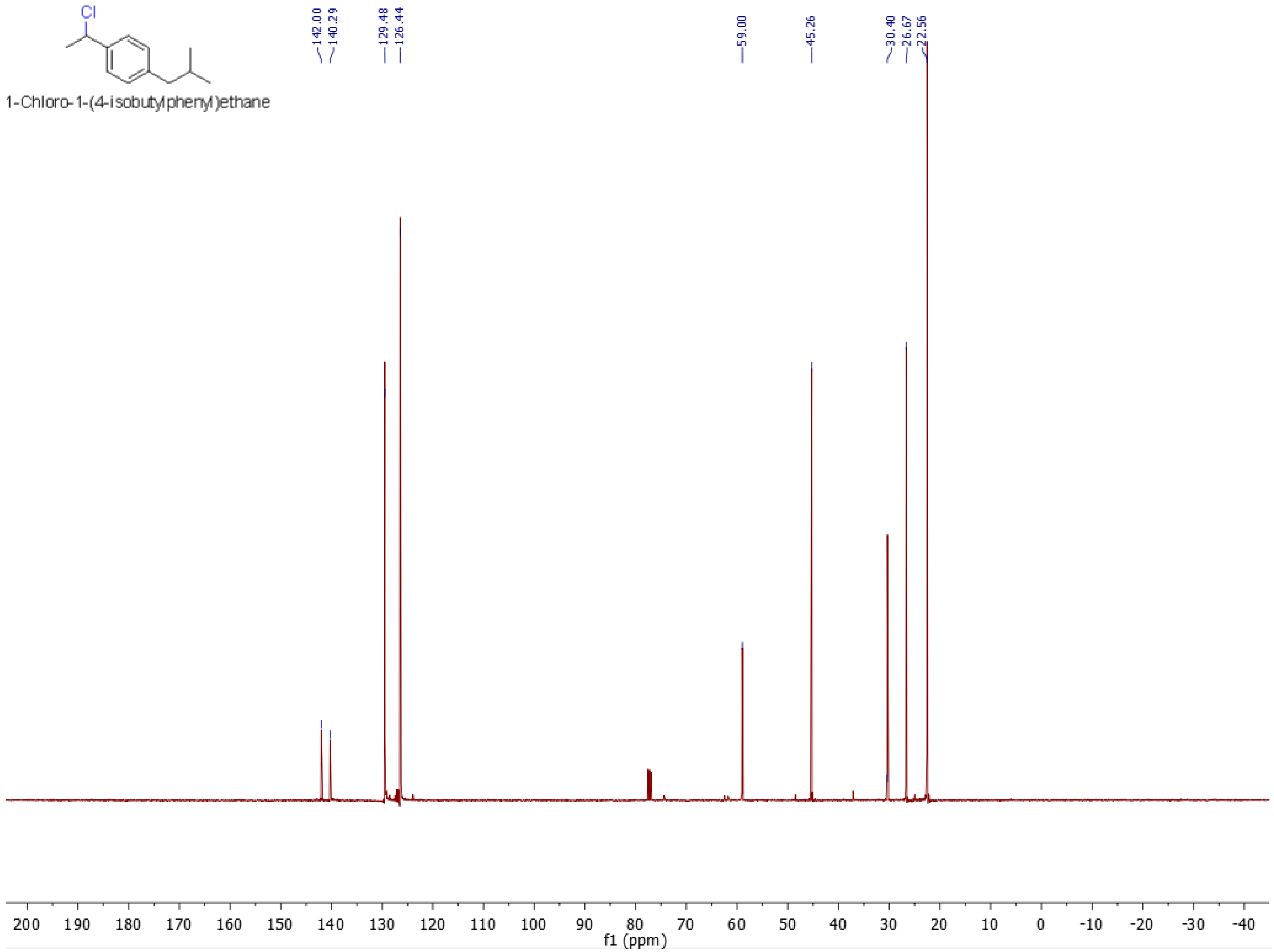
Cl
1-Chloro-1-(4-isobutylphenyl)ethane
142.00
140.29
129.48
126.44
59.00
45.26
30.40
26.67
22.56
200
190
180
170
160
150
140
130
120
110
100
90
80
70
60
50
40
30
20
10
0
-10
-20
-30
-40
f1 (ppm)

^{1}H NMR

6 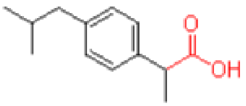

2-(4-isobutylphenyl)propanoic acid

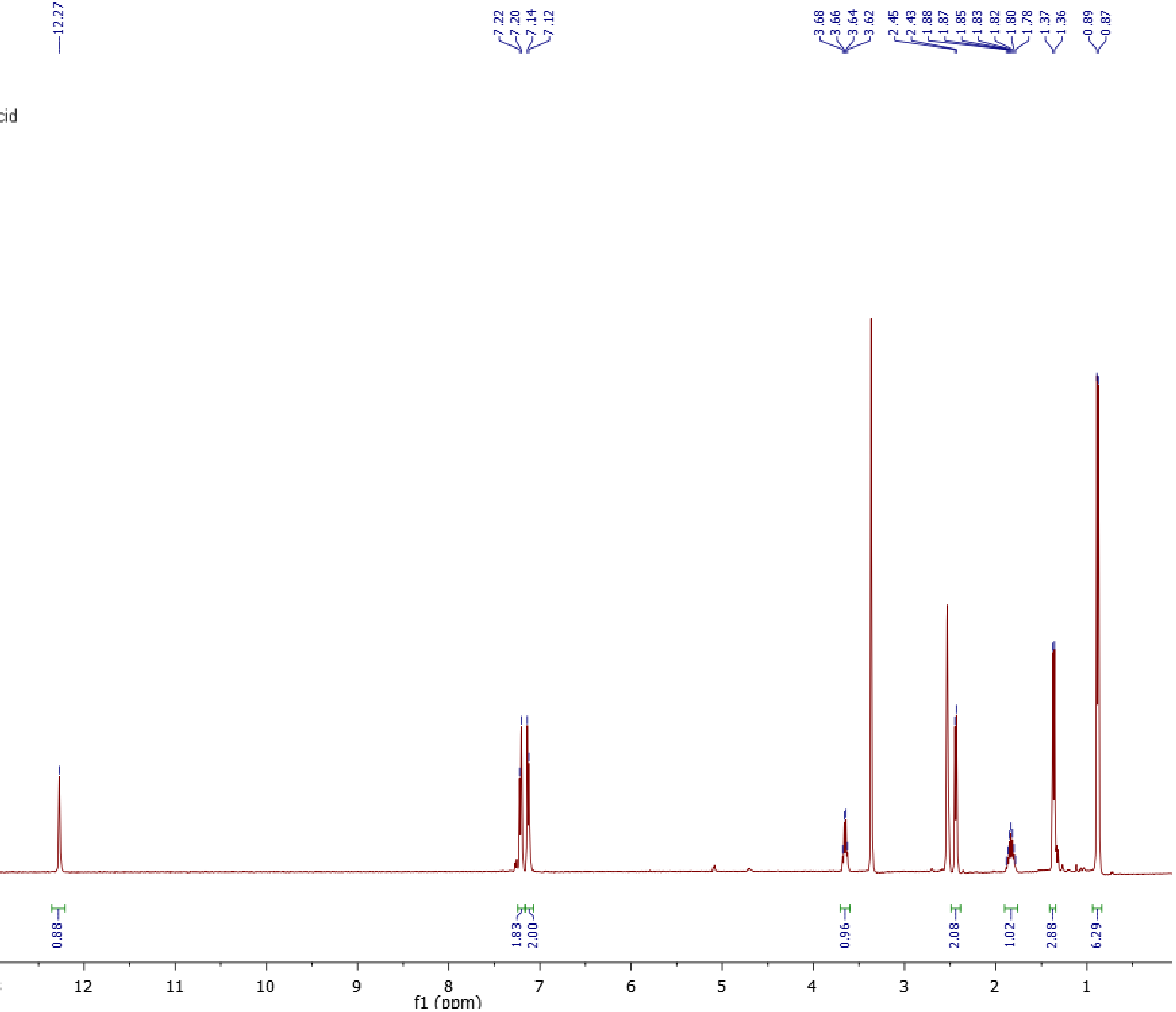

^{13}C NMR

6

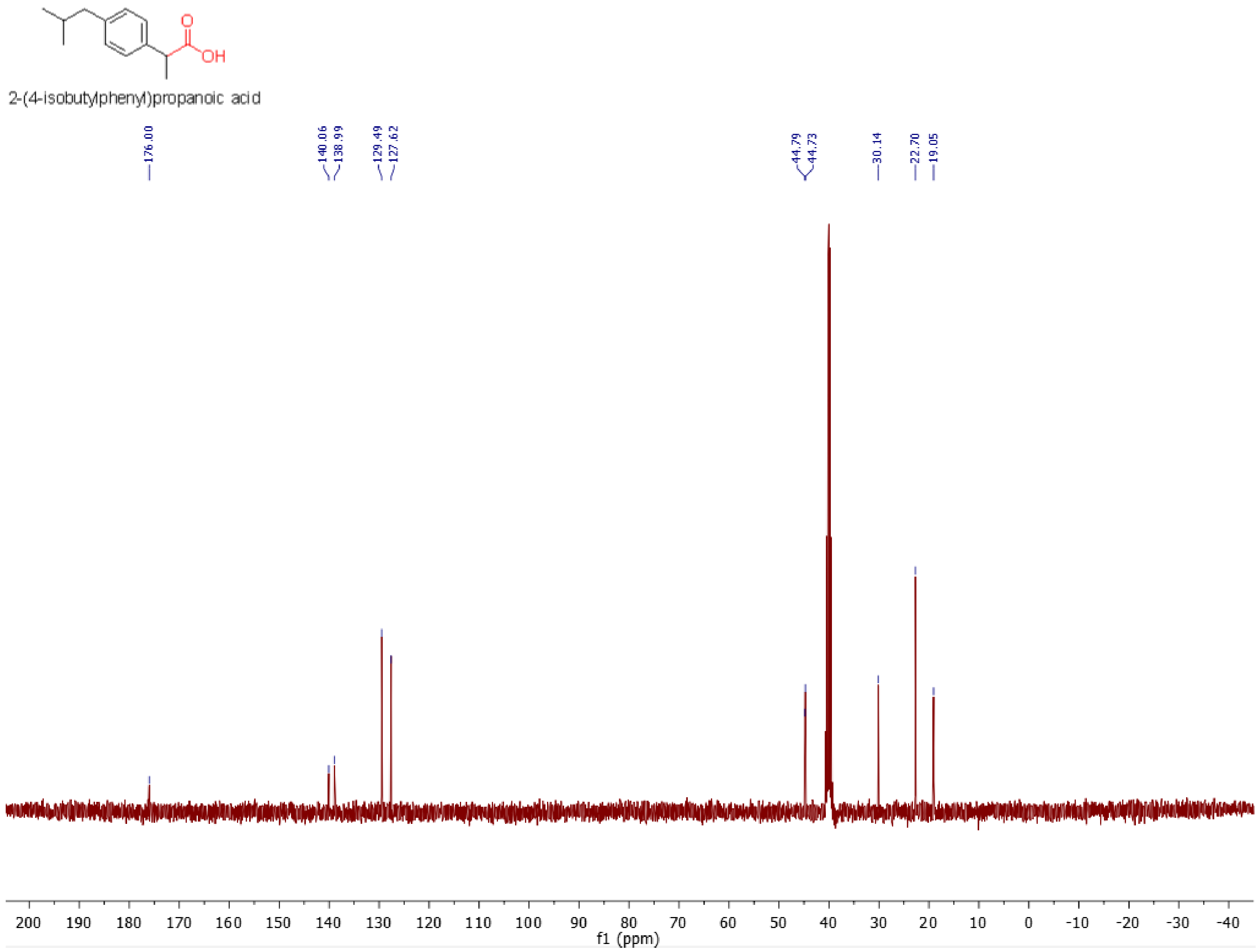

OH
2-(4-isobutylphenyl)propanoic acid
176.00
140.06
138.99
129.49
127.62
44.79
44.73
30.14
22.70
19.05
200 190 180 170 160 150 140 130 120 110 100 90 80 70 60 50 40 30 20 10 0 -10 -20 -30 -40
f1 (ppm)

Cyclic Voltamperometry

Cyclic voltammetries were carried out on a Metrohm Autolab PGSTAT204 workstation. In a 30 mL three-necked electrochemical cell fitted with a vitreous carbon (VC) disk working electrode (Ø = 3 mm), gold wire counter electrode and Ag/AgCl reference electrode were added DMF (20 mL) and NBu_4BF_4 (0.3 M) as supporting electrolyte. The solution was deoxygenated by bubbling with argon for 5 minutes under stirring before the addition of the substrates (0.05 M). Measurements were carried out at room temperature without stirring at a scan rate of 50 mV/s using an applied potential ranging from 0 to 2.5 V (oxidative scans) or from 0 to -3.5 V (reductive scans). Neutral atmosphere is maintained by letting argon above the solution during measurements. After each measurement, the working electrode was polished with an abrasive disk (type M, Ø = 200 mm, P2400).

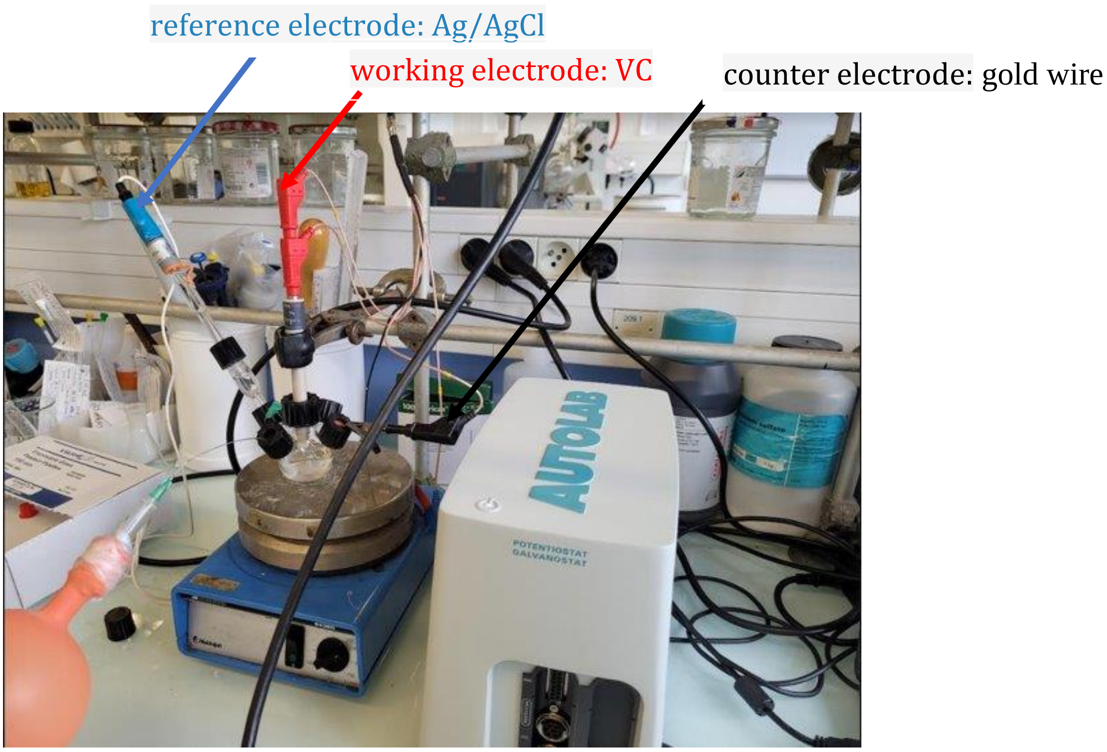

Figure 7: Cyclic voltammetry assembly

Blank: DMF (20 mL), NBu_4BF_4 (0.3 M)

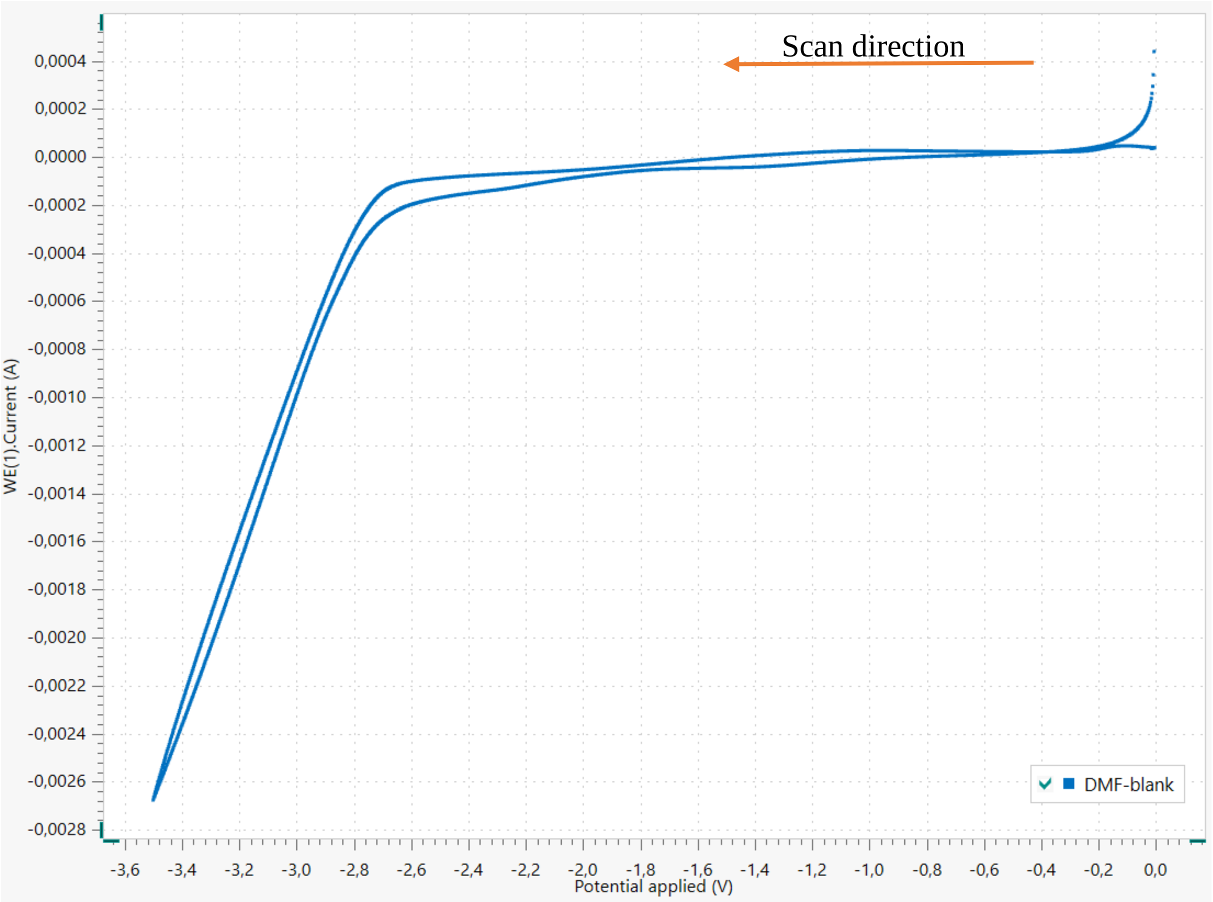

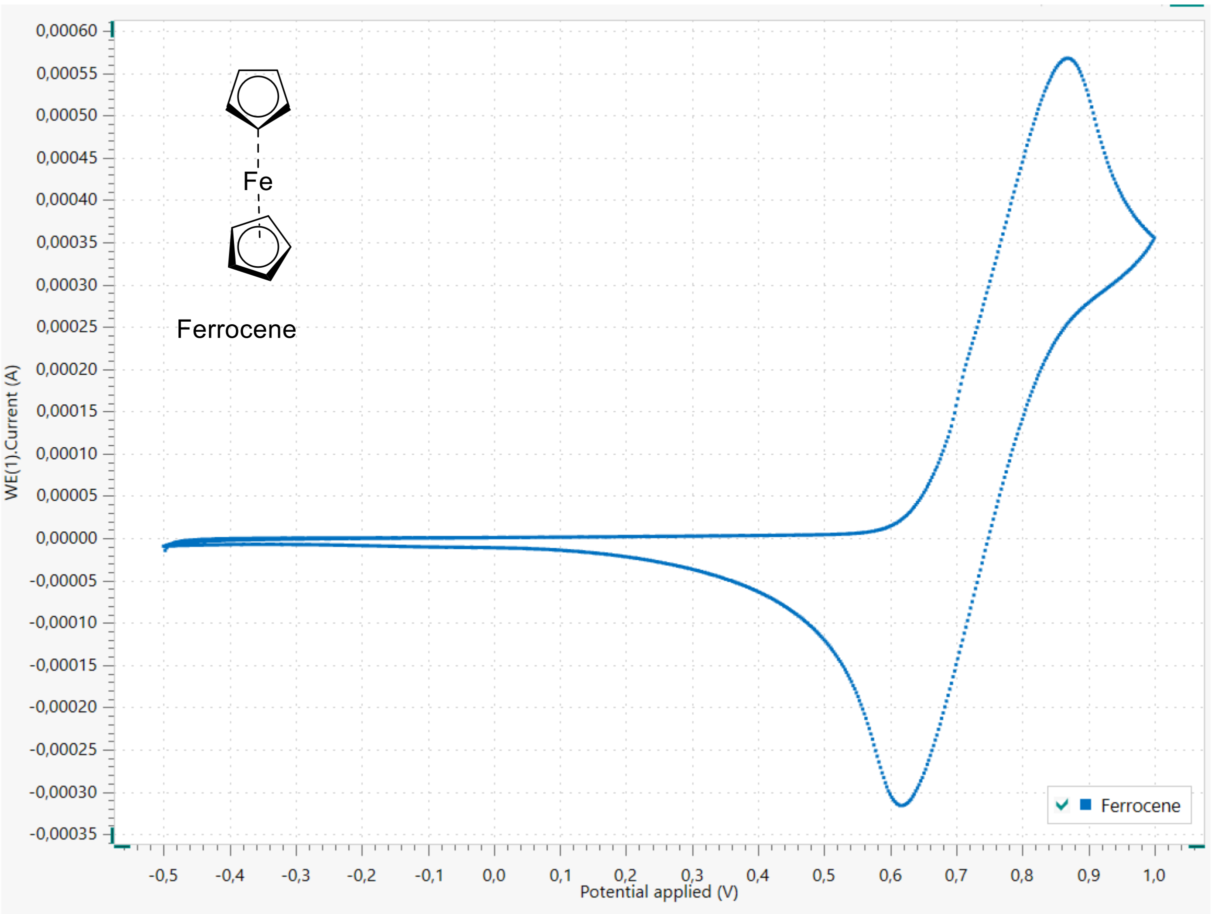

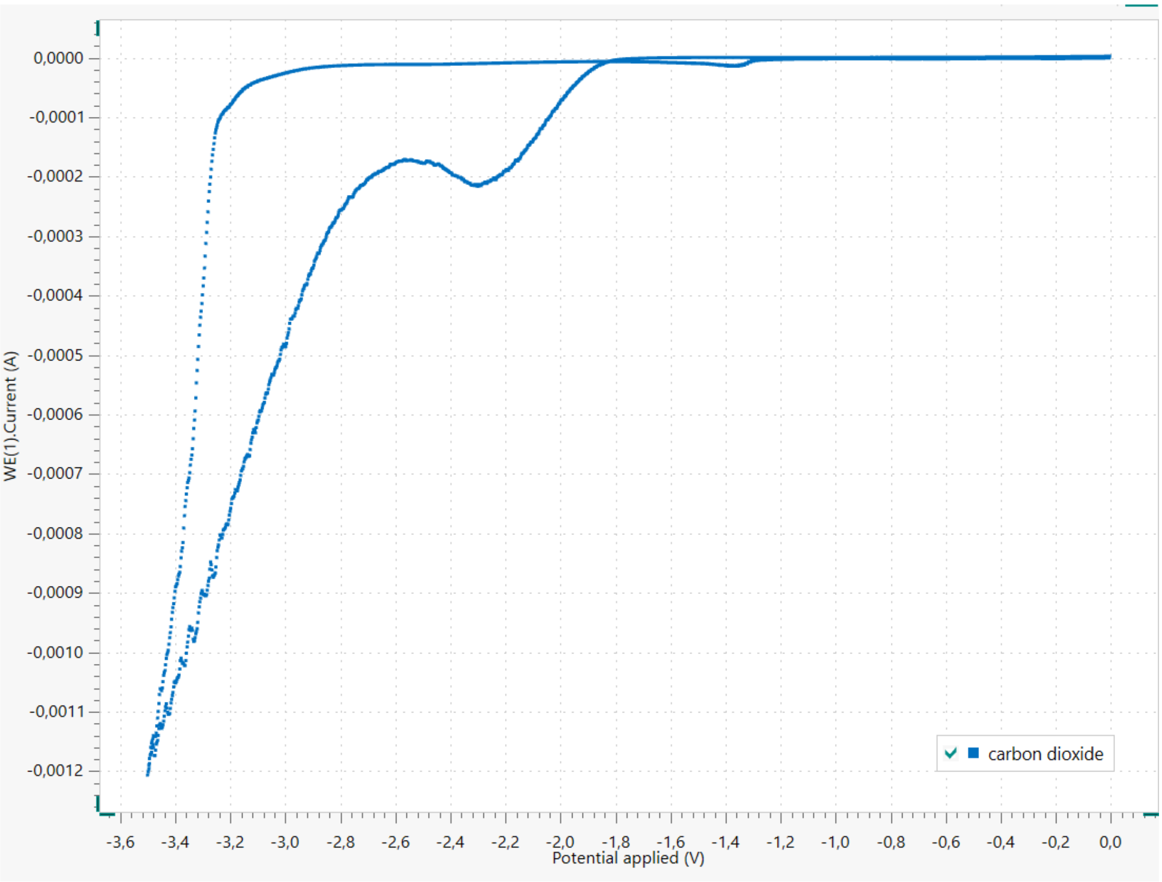

0,0000
-0,0001
-0,0002
-0,0003
-0,0004
-0,0005
-0,0006
-0,0007
-0,0008
-0,0009
-0,0010
-0,0011
-0,0012
WE(1).Current (A)
-3,6 -3,4 -3,2 -3,0 -2,8 -2,6 -2,4 -2,2 -2,0 -1,8 -1,6 -1,4 -1,2 -1,0 -0,8 -0,6 -0,4 -0,2 0,0
Potential applied (V)
carbon dioxide
